# Autoregulation of GPCR signalling through the third intracellular loop

**DOI:** 10.1038/s41586-023-05789-z

**Published:** 2023-03-08

**Authors:** Fredrik Sadler, Ning Ma, Michael Ritt, Yatharth Sharma, Nagarajan Vaidehi, Sivaraj Sivaramakrishnan

**Affiliations:** 1 Biochemistry, Molecular Biology and Biophysics Graduate Program, University of Minnesota, Minneapolis, MN USA; 2 Department of Genetics, Cell Biology and Development, University of Minnesota, Minneapolis, MN USA; 3 Irell and Manella Graduate School of Biological Sciences, Beckman Research Institute of the City of Hope, Duarte, CA USA; 4 Department of Computational and Quantitative Medicine, Beckman Research Institute of the City of Hope, Duarte, CA USA

**Keywords:** Cell signalling, Mechanism of action

## Abstract

The third intracellular loop (ICL3) of the G protein-coupled receptor (GPCR) fold is important for the signal transduction process downstream of receptor activation^1–3^. Despite this, the lack of a defined structure of ICL3, combined with its high sequence divergence among GPCRs, complicates characterization of its involvement in receptor signalling^4^. Previous studies focusing on the β_2_ adrenergic receptor (β_2_AR) suggest that ICL3 is involved in the structural process of receptor activation and signalling^5–7^. Here we derive mechanistic insights into the role of ICL3 in β_2_AR signalling, observing that ICL3 autoregulates receptor activity through a dynamic conformational equilibrium between states that block or expose the receptor’s G protein-binding site. We demonstrate the importance of this equilibrium for receptor pharmacology, showing that G protein-mimetic effectors bias the exposed states of ICL3 to allosterically activate the receptor. Our findings additionally reveal that ICL3 tunes signalling specificity by inhibiting receptor coupling to G protein subtypes that weakly couple to the receptor. Despite the sequence diversity of ICL3, we demonstrate that this negative G protein-selection mechanism through ICL3 extends to GPCRs across the superfamily, expanding the range of known mechanisms by which receptors mediate G protein subtype selective signalling. Furthermore, our collective findings suggest ICL3 as an allosteric site for receptor- and signalling pathway-specific ligands.

## Abstract (web summary)

Biochemical and molecular dynamics studies show that the third intracellular loop of G protein-coupled receptors autoregulates the receptor activity and tunes the signalling specificity by controlling access to the G protein-binding site.

## Main

Accumulating structural data are increasingly enabling atomic-resolution mapping of the activation mechanisms of GPCRs. This fine detail can be used to design therapeutic agents that target specific GPCRs implicated in diverse disease states^8^. Although GPCR activation is best understood through conformational changes in the seven transmembrane helices of the receptor, the termini and loop domains connecting these helices are also critical for receptor function and regulation in a cellular context^9^. Owing to the inaccessibility of these regions to traditional structural methods, there is a lack of insight into how they contribute to GPCR signalling mechanisms. Focused characterization of the dynamics of these regions would refine our understanding of their roles in GPCR signalling, with the potential to identify novel therapeutic strategies^4^.

**Extended Data Fig. 1:**
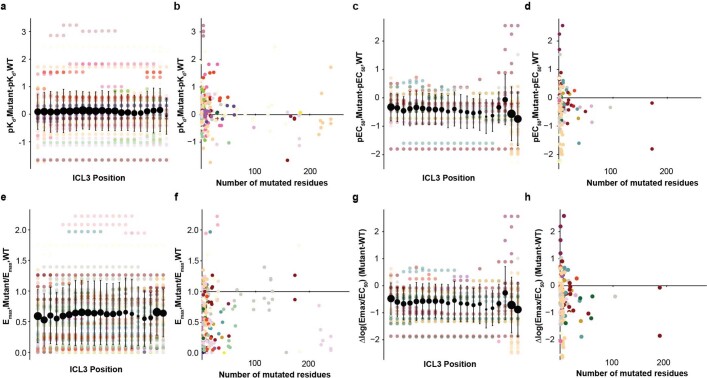
Meta-analysis of effects of ICL3 mutagenesis on receptor function (See Methods, Supplementary Table 1 for details). **a**. Influence of mutagenized ICL3 position and **b**. size of ICL3 mutation on agonist binding affinity (pK_d_) (N = 228 mutations, 32 receptors, 62 studies). **(c-d)** compare the same effects on signaling, potency (pEC_50_) (N = 146 mutations, 18 receptors, 30 studies), (**e-f)** on maximum signaling output (Emax) (N = 258 mutations, 27 receptors, 58 studies), and **(g-h)** on signaling efficacy (log(E_max_/EC_50_) (N = 145 mutations, 17 receptors, 29 studies). WT, wild-type receptor. Large points reflect mean ± standard deviation of mutagenizing each site in ICL3. Small, translucent points reflect individual experimental data points, colored by receptor identity.

Here we focus on ICL3, which is the largest of the three intracellular loops in many class A GPCRs, ranging from 10–240 amino acids in size. ICL3 connects transmembrane helices five and six, which are responsible for structural changes between the receptor’s inactive and active states, and is adjacent to the receptor’s signalling-effector-binding site^10^. The physical location of ICL3 corroborates a large body of mutagenesis studies that implicate this region in receptor activation and signalling (Supplementary Table 1). However, changes in receptor pharmacology upon mutagenesis of ICL3 vary widely between receptors, as well as between the locations of sites mutated on individual receptors (Extended Data Fig. 1). Given this lack of consensus, the mechanisms by which ICL3 influences receptor activation across receptors remain poorly understood. This is exacerbated by the sequence diversity of ICL3, even among closely related receptors^11^. Additionally, the predicted intrinsic disorder and lack of structural resolution of ICL3 in most published structures limit structure-to-function characterization^12^. In this study, we address this knowledge gap by advancing a fundamental conceptual framework for the role of ICL3 in GPCR signalling.

## A FRET-based approach to probe ICL3 conformation

We focused our initial mechanistic study on ICL3 of β_2_AR, a structural prototype for GPCR study^13^. Molecular modelling of β_2_AR suggests that its ICL3 can pack into the receptor’s intracellular cavity, potentially regulating the activation of signalling effectors downstream of the receptor^5^. This packed conformation of ICL3 has been proposed to communicate allosterically with the receptor’s extracellular domain, leading to tight coordination between the receptor’s activation state and ICL3 conformation^14^. In parallel, mutagenesis of ICL3 alters receptor conformational dynamics, as measured using ^19^F-NMR spectroscopy^6^. With these insights as a foundation, we aimed to build a mechanistic model for ICL3 function in β_2_AR activation and signalling by determining its conformational ensemble.

To track the conformational dynamics of the β_2_AR ICL3, we drew inspiration from previous efforts using organic fluorophores conjugated to different residues of the receptor^15,16^. These techniques provided key insights into conformational changes in the transmembrane helices that have subsequently been verified in high-resolution crystal structures^17^. Furthermore, the smaller footprint of these fluorophores (similar in molecular mass to 2–3 amino acids) compared with fluorescent protein variants is desirable from the perspective of discerning conformational changes within protein regions^18,19^. In translating this technique to ICL3, we modified the method to preserve the integrity of the receptor in a native cell membrane environment.

**Extended Data Fig. 2:**
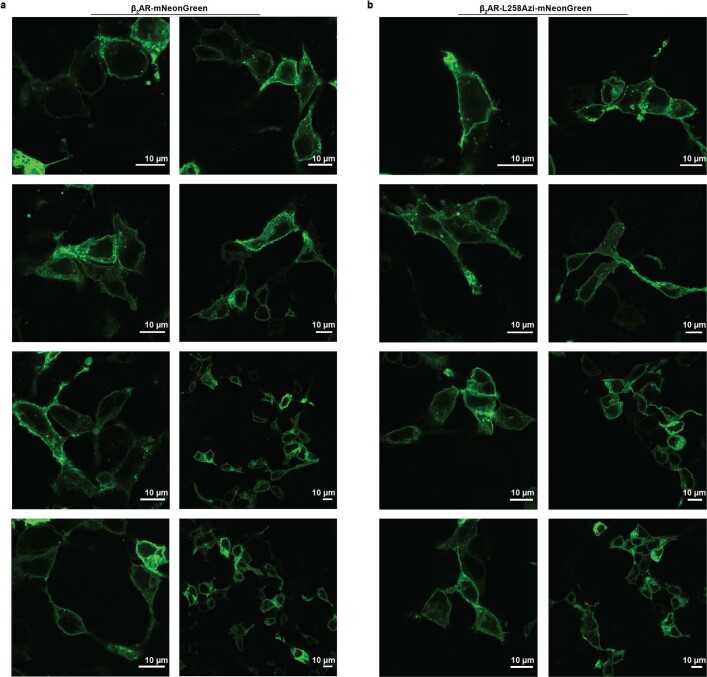
Representative confocal scanning images of mNeonGreen fluorescence for (**a**) β_2_AR wild-type (WT) (Δ350-413)-mNeonGreen and (**b**) β_2_AR L258Azi (Δ350-413)-mNeonGreen. Scale bar indicates 10 µm. Experiments for which images were collected were performed three times.

**Extended Data Fig. 3:**
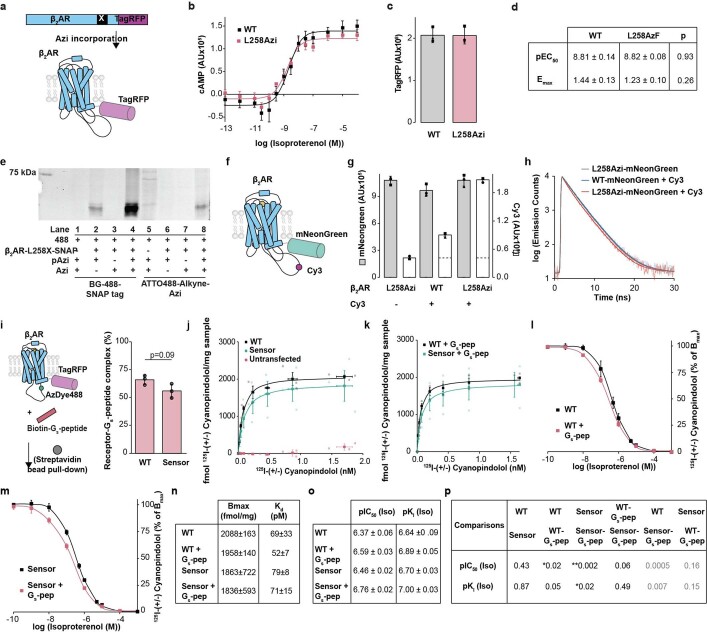
Pharmacological and biochemical characterization of L258Azi incorporation and bio-orthogonal click chemistry. **a**. Schematic of stop codon suppression. L258 in the ICL3 of C-terminally truncated β2AR (Δ350-413)-TagRFP is mutagenized to an amber stop codon (TAG). 4-azido-L-phenylalanine (Azi) is incorporated by charging amber suppressor tRNA_CUA_ with Azi using an engineered synthetase specific for Azi^20^. **b**. Isoproterenol dose - cAMP accumulation response for overexpressed β2AR-(Δ350-413)-TagRFP constructs. Points are mean ± standard error of mean of three biological replicates. Curves are the fit of the mean data (Methods). **c**. Expression levels of constructs in (b) as measured by TagRFP fluorescence at equivalent cell counts. Bars indicate mean ± standard deviation, points represent three independent experiments. **d**. Table of fit parameters from (b). Values indicate mean ± standard error of mean of three independent experiments. Statistical comparisons shown are from unpaired two-sided t-tests comparing WT and L258 Azi (pEC_50_: t = −0.09, 4 degrees of freedom; E_max_: t = −1.33, 4 degrees of freedom). **e**. Fluorescence gel scanning (ex 473 nm, 510- long pass emission scan; complete gel scan is included in Supplementary Fig. 1) Lanes 1-4 (numbered left to right; left half of gel) - incorporation of Azi into β2AR L258x (Δ350-413) SNAP (β_2_AR-L258X-SNAP), measured by detection with SNAP Surface Block 488 (BG-488) (Expected molecular weight 60 kDa). Deviations in expected vs apparent molecular weight have been previously reported in helical membrane proteins^67^. Membrane extracts from cells transfected without (−) and with (+) the vector containing the receptor mutant (Lane 1 vs 4); transfected +/− the vector containing the amber suppressor tRNA_CUA_ and Azi tRNA Synthetase (pAzi) (Lane 3 vs 4); and incubated +/− 0.5 mM of the unnatural amino acid Azi (Azi) (Lane 2 vs 4). Highest band intensity is seen when all components are present. A band of lighter intensity is seen in the absence of Azi (lane 2), which are likely attributable to charging of tRNA_CUA_ by the Azi tRNA synthetase with other amino acids under our transfection conditions. Lanes 5-8 (right half of gel), click-chemistry labeling of β2AR L258Azi (Δ350-413) SNAP with ATTO488-Alkyne (equivalent fluorophore to BG-488) using the same controls as lanes 1-4. Highest band intensity is seen when all components are present. Non-specific bands are seen in the absence of receptor mutant, which is attributable to labeling of Azi-suppressed amber stop codons of endogenous proteins (23% of open reading frames in the Human genome have TAG stop codons^68^) with ATTO488-Alkyne. Lighter intensity of lane 8 band compared to lane 4 band is likely due to incomplete labeling with ATTO488-Alkyne, which could reduce apparent basal FRET. Experiment for which scan was collected was performed in parallel three times. **f**. Schematic of β_2_AR L258Azi (Δ350-413) mNeonGreen, with L258Azi labeled with Cy3-Alkyne. **g**. Labeling specificity of construct depicted in **(f)**. Grey bars, mNeonGreen fluorescence (ex470/em515). White bars, Cy3 fluorescence (ex535/em565). Bars represent mean ± standard deviation of three independent experiments. Dotted line on Cy3 bars represents mNeonGreen cross-excitation (β_2_AR L258Azi (Δ350-413) mNeonGreen without Cy3). With this considered, the labeling conditions yield a ~3:1 signal (β_2_AR L258Azi (Δ350-413) mNeonGreen with Cy3-Alkyne) to noise (β_2_AR wild-type (WT) (Δ350-413) mNeonGreen with Cy3) ratio, which could also diminish apparent basal FRET **h**. Representative mNeonGreen fluorescence decay curves of the labeling conditions in (**g**). Only the specific labeling condition results in an observable left-shift in fluorescence decay. **i**. Receptor-G_s_ peptide complex formation. *Left*, schematic of G_s_-peptide pulldown assay used to assess complex formation. Membrane extracts of cells containing β2AR (Δ350-413)-TagRFP are pulled down onto streptavidin-coated magnetic beads via 30 μM of N-terminally Biotinylated G_s_-peptide. *Right*, Biotin-G_s_-peptide pulldown measurements comparing β_2_AR Δ350-413 TagRFP (WT) and β_2_AR L258Azi Δ350-413 TagRFP labeled with AZDye488 (Sensor). Bars represent mean ± standard deviation of 3 independent experiments. Points represent experimental replicates. Statistical comparison shown is from an unpaired two-sided t-test (t = 2.28, 4 degrees of freedom). **j**. ^125^I-(+/−)-Cyanopindolol binding to membrane extracts of WT, sensor, and endogenous adrenergic receptors (Untransfected). **k**. ^125^I-(+/−)-Cyanopindolol binding to membrane extracts of WT and Sensor in the presence of 30 μM G_s_-peptide. **l**. Competition binding between ^125^I-(+/−)-Cyanopindolol and Isoproterenol for WT and **m**. Sensor. Binding assays in l were performed in the presence and absence of 30 μM G_s_-peptide. For (**j—m**), opaque points represent the mean ± standard error of mean of 3 independent experiments. Curves represent the fit of the mean data to a binding model (see Methods). For (**j**) and (**k**), transparent points represent experimental replicates. **n**. Table of fit parameters from ^125^I-(+/−)-Cyanopindolol binding assays in (**j**) and (**k**) and **o**. competition binding assays in (**l**) and (**m**) (mean ± standard error of mean of three independent experiments). **p**. Table of p-values from post-hoc statistical comparisons. Comparisons shown are from two-way ANOVA followed by Tukey’s post-hoc significance test where ** indicates p < 0.01 and * indicates p < 0.05 (pIC_50_: Factor 1 (WT vs Sensor): F = 11.1, p = 0.01, Factor 2 (G_s_-peptide treatment): F = 44.5, p = 2e-4, Factor 1* Factor 2: F = 1.2, p = 0.3; pK_i_: Factor 1: F = 2.5, p = 0.2, Factor 2: F = 25.3, p = 1e-3, Factor 1* Factor 2: F = 0.3, p = 0.6). Grey indicates an insignificant interaction from ANOVA (p ≥ 0.05). Source data

Our technique uses a single amino acid substitution in residue L258 in ICL3 of the receptor. We mutagenized L258 to the unnatural amino acid 4-azido-l-phenylalanine (Azi) using stop codon suppression^20^ (Extended Data Fig. 2). We conjugated fluorescent probes to this site in crude membrane extracts using bio-orthogonal click chemistry (Extended Data Fig. 3). The modifications made to the receptor maintain membrane localization patterns (Extended Data Fig. 2b), second messenger signalling (Extended Data Fig. 3b) and radioligand-binding properties (Extended Data Fig. 3j–m) of wild-type β_2_AR.

**Fig. 1:**
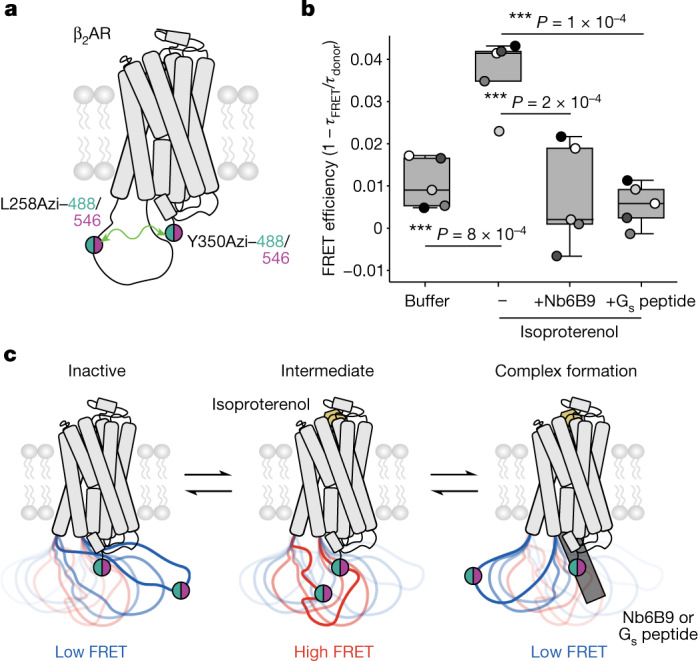
Agonist- and cytoplasmic effector-binding proteins drive conformational changes in ICL3. **a**, Schematic of the β_2_AR ICL3 FRET sensor. Membrane extracts of cells expressing β_2_AR(L258Azi/Y350Azi/∆351–413) were labelled with Alkyne-AZDye488 and Alkyne-AZDye546 to generate the sensor. **b**, FRET efficiency of untreated sensor (buffer), sensor treated with isoproterenol (100 µM), sensor treated with isoproterenol and nanobody Nb6B9 (500 nM) or sensor treated with isoproterenol and 10 µM G_s_ peptide (10 µM). FRET efficiency is defined as 1 − *τ*_FRET_/*τ*_donor_, where *τ*_FRET_ is the average lifetime of the FRET sensor (Extended Data Fig. 4p, grey bars) and *τ*_donor_ is the average lifetime of an AZDye488-only-labelled control sample (Extended Data Fig. 4p, white bars). Box edges delineate the 1st and 3rd quartiles of the data, the centre line represents the median, whiskers represent the furthest points within 1.5× the interquartile rangean and points represent five independent experiments. One-way ANOVA followed by Tukey’s post hoc significance test; ***P < 0.001 (*F* = 15.2, *P* = 6 × 10^−6^, 16 d.f.). **c**, Proposed sensor readout of ICL3 conformational equilibrium. Left, in the receptor’s inactive state, the donor and acceptor probes are further apart, resulting in low FRET. Centre, agonist (isoproterenol) binding increases probe proximity, thereby increasing FRET efficiency (intermediate). Right, formation of agonist–receptor–effector (with Nb6B9 or G_s_ peptide) complex displaces ICL3 from the intracellular cavity, extending the distance between donor and acceptor probes and quenching the FRET readout. Source data

**Extended Data Fig. 4:**
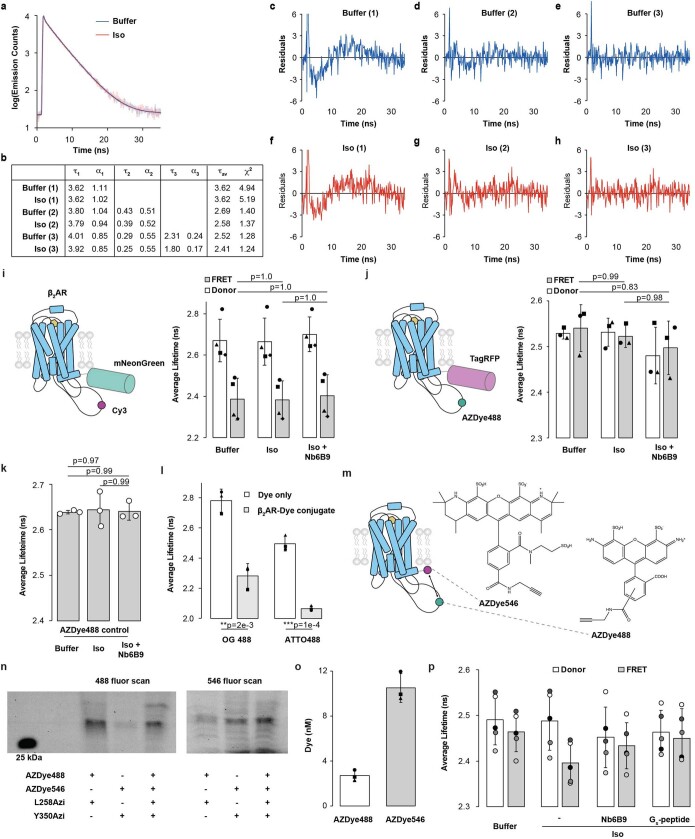
Analysis and optimization of β_2_AR ICL3 FRET sensor. Time-correlated single photon counting data used to derive fluorescence lifetime values. **a**. Representative decay curves of β_2_AR ICL3 FRET sensor untreated (Buffer) or treated with Isoproterenol (100 µM). Transparent traces represent collected data, and opaque lines represent fits of data to a 3-exponential decay model (Methods). **b**. Table of lifetimes (*τ*, ns) and amplitudes (*α*, arbitrary units), for single exponential (1), double exponential (2), and three exponential (3) fits. Comparison of goodness-of-fit for the (**c—e**) buffer condition and for the (**f—h**) Iso condition. Traces are the residuals between the data and a 1-exponential decay, 2-exponential decay, and 3-exponential decay. The systematic overestimation (0-5 ns,10-30 ns) or underestimation (5-10 ns) of residuals for the 1- and 2-exponential fits suggests use of a 3-exponential fit to estimate the average lifetime (χ2) more accurately. (**i—p**) Sensor designs (*illustrated in i, j, m*) based on β_2_AR L258Azi (Δ350-413). Donor/Acceptor pairs are formed between fluorophores located at β_2_AR L258 and the truncated C-terminus. *Summary -* β_2_AR with dual AZDyes (488 and 546) shows robust changes in donor fluorophore lifetimes in response to agonist and G protein site effector. **i**. Membrane extracts of cells expressing β_2_AR L258Azi (Δ350-413) mNeonGreen labeled with Cy3-Alkyne (n = 4 independent experiments) or (**j)** β_2_AR L258Azi (Δ350-413) TagRFP labeled with AZDye488-Alkyne (n = 3 independent experiments). For (**i**) and (**j**): Left, schematic of sensor. Right, Average fluorescence lifetime (**i**-mNeonGreen or **j**-AzDye488) of untreated sensors (Buffer), sensors treated with the agonist Isoproterenol (100 µM), or sensors treated with Iso and nanobody Nb6B9 (500 nM). White bars designate donor only controls (**i**- β_2_AR L258Azi (Δ350-413) mNeonGreen without Cy3-Alkyne label, **j**- β_2_AR L258Azi (Δ350-413) SNAP labeled with Alkyne-AZDye488). Grey bars designate FRET sensor with both donor and acceptor fluorophores present. Statistical comparisons shown from a two-way ANOVA followed by Tukey’s post-hoc significance test where NS indicates *p* ≥ 0.05 (**i**-Factor 1 (Donor vs FRET): F = 49.5, p = 1.5e-6, Factor 2 (Buffer vs Iso vs Iso+Nb): F = 0.2, p = 0.84, Factor 1* Factor 2: F = 0.014, p = .986, 18 degrees of freedom; **j**- Factor 1 (Donor vs FRET): F = 0.1, p = 0.76, Factor 2 (Buffer vs Iso vs Iso+Nb): F = 1.9, p = 0.20, Factor 1* Factor 2: F = 0.15, p = .86, 12 degrees of freedom). **k**. Fluorescence lifetime of Alkyne-AZDye488 added free in solution to β_2_AR (Δ350-413) wild-type TagRFP membranes (n = 3 independent experiments). Statistical comparisons shown from a one-way ANOVA followed by Tukey’s post-hoc significance test where NS indicates *p* ≥ 0.05 (F = 0.03, p = .97, 6 degrees of freedom) **l**. Fluorescence lifetime of OG 488-Alkyne and ATTO488-Alkyne (n = 3 independent experiments). White bars represent measurements of dye free in solution, and grey bars represent dyes conjugated to membrane extracts from cells expressing β2AR L258Azi (Δ350-413) SNAP. Conjugation of any of the three donor dyes to β_2_AR L258Azi decreases fluorescence lifetime even the absence of FRET acceptor. Statistical comparisons shown are from two-sided unpaired t-tests (OG 488: t = 7.7, 4 degrees of freedom; ATTO488: t = 14.3, 4 degrees of freedom) **m**. Schematic of β2AR L258Azi Y350Azi (∆351-413) labeled with Alkyne-AZDye488 and Alkyne-AZDye546. Membrane extracts of cells expressing the β2AR double mutant were labeled with both dyes to generate the FRET sensor. Right, chemical structure of both dyes. **n**. Gel scanning of dyes incorporated into L258Azi and Y350Azi (complete gel scan is included in Supplementary Fig. 2). Membrane extracts of cells expressing β_2_AR (Δ350-413), with either a single Azi incorporation site or both incorporation sites (indicated as L258Azi (+/−) or Y350Azi (+/−)), were conjugated to AZDye488-Alkyne, AZDye546-Alkyne, or both dyes (expected molecular weight 40 kDa)^67^. Left, 488 fluor scan (ex 473 nm, 510- long pass emission scan). Right, 546 fluor scan (ex 532 nm, 570- long pass emission scan) of the same gel. Bands in lane 2 on the left and lane 1 on the right can be attributed to cross-excitation (10-15% cross-excitation is expected on the excitation wavelengths and the long-pass filters available on the instrument). **o**. Concentration of each dye in membrane extracts containing sensor depicted in (**m**) (n = 3 independent experiments). Dye concentrations were assessed from sample emissions in a fluorometer (at emission maximum) and concentration standards. **p**. Average fluorescence lifetime of sensors (n = 5 independent experiments) under Buffer, Isoproterenol (100 µM), Isoproterenol with Nb6B9 (500 nM), or Isoproterenol with G_s_-peptide (10 µM) treatment. White bars designate donor only controls (β_2_AR L258Azi (Δ350-413) SNAP labeled with AZDye488-Alkyne). Grey bars designate FRET sensor. For all bar graphs (**i—l,o,p**), bars represent mean ± standard deviation. Points represent individual experiments. Source data

We measured the conformational changes in ICL3 via changes in fluorescence lifetime. Proximity of a fluorophore conjugated at L258Azi to a second fluorophore at the truncated C terminus of the receptor (∆350–413) is expected to decrease fluorescence lifetime of the donor fluorophore by fluorescent resonance energy transfer (FRET) to the acceptor fluorophore (Extended Data Fig. 4). Sensor optimization revealed that conjugating AZDye488 and AZDye546 to L258Azi and Y350Azi could resolve changes in lifetime based on the activation state of the receptor (Extended Data Fig. 4p). Treatment of this sensor with the agonist isoproterenol increases FRET efficiency (by around 4%) relative to buffer alone (Fig. 1b). FRET efficiency is quenched when the sensor is treated with isoproterenol in combination with a nanobody that binds the receptor’s cytosolic cavity in the active state^21^ (Nb6B9). Similar quenching is observed upon treatment with isoproterenol and a peptide composed of the α_5_ helix of the α-subunit of the G_s_ protein (G_s_-peptide), the structural element of the G protein that interacts with the cytoplasmic core of the receptor. These data suggest a three-state model for ICL3 conformation: an inactive (low-FRET) state, an agonist-stimulated intermediate (high-FRET) state, and an effector-bound (low-FRET) state (Fig. 1c).

## Conformational landscape of ICL3

**Fig. 2:**
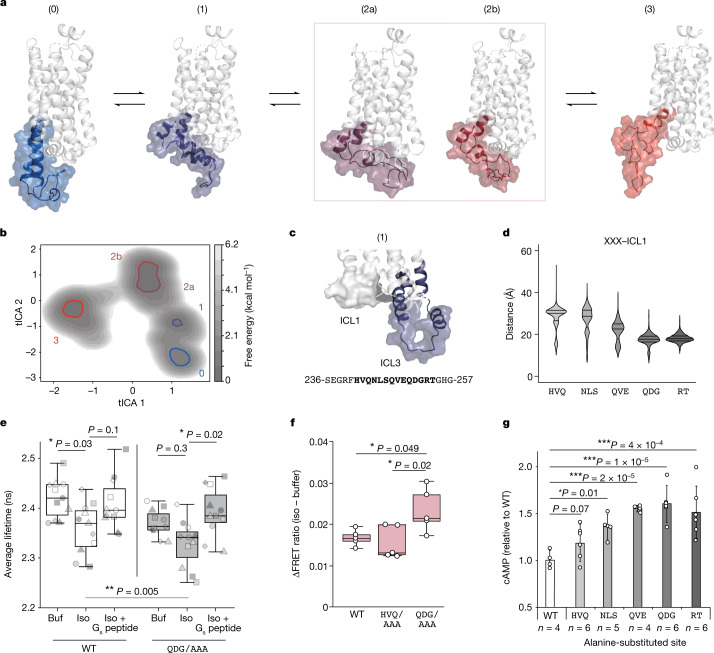
The conformation of ICL3 controls the accessibility of the G protein-binding cavity. **a**, Representative structures of β_2_AR, highlighting states of ICL3 in molecular dynamics simulations. **b**, Free energy landscape derived from simulations. *x*- and *y*-axes represent the largest time-correlated independent components (tICA 1 and tICA 2) from simulation dihedral angles. Free energy local minima represented by structures in **a** are highlighted. **c**, Top, the structure of state 1, showing the proximity of ICL3 to ICL1. Bottom, the ICL3 residues 236–257, with distance-constrained residues in bold. **d**, The distance between indicated three-amino-acid segments of ICL3 to ICL1 for simulation trajectories in intermediate cluster 1 (*n* = 11,648 molecular dynamics snapshots). Lines represent quartiles of each dataset. X represents any amino acid. **e**, Fluorescence lifetime measurements of AZDye488 in the ICL3 FRET sensor, comparing wild-type (WT) receptor with Q250–G252 mutated to AAA (QDG/AAA). Measurement conditions: untreated (buf), treated with isoproterenol (iso) (100 μM), or treated with isoproterenol (100 μM) and G_s_ peptide (10 μM) (*n* = 11 independent experiments). **f**, Agonist-induced change (∆) in FRET ratio (isoproterenol (100 µM) − buffer) for β_2_AR–G_s_ peptide interactions (*n* = 5 independent experiments). In **e**,**f**, box edges delineate the 1st and 3rd quartiles of the data, the centre line represents the median and whiskers represent points within 1.5× the interquartile range. **g**, cAMP accumulation for wild-type β_2_AR and 5 alanine-scanning mutants of ICL3 at a saturating concentration of isoproterenol (10 µM). Data are mean ± s.d. *n* represents independent biological samples (indicated on figure) from six experiments. One-way (**f**,**g**) or two-way (**e**) ANOVA followed by Tukey’s post hoc test; ***P< 0.001, **P < 0.01 and *P < 0.05. **e**, Factor 1 (buffer versus isoproterenol versus isoproterenol + G_s_ peptide): *F* = 10.6, *P* = 0.0001; factor 2 (wild type versus QDG/AAA): *F* = 8.5, *P* = 5 × 10^−3^; factor 1 × factor 2: *F* = 0.7, *P* = 0.5, 60 d.f. **f**, *F* = 5.7, *P* = 0.02, 12 d.f. **g**, *F* = 13.2, *P* = 6.9 × 10^−6^, 21 d.f. Source data

**Extended Data Fig. 5:**
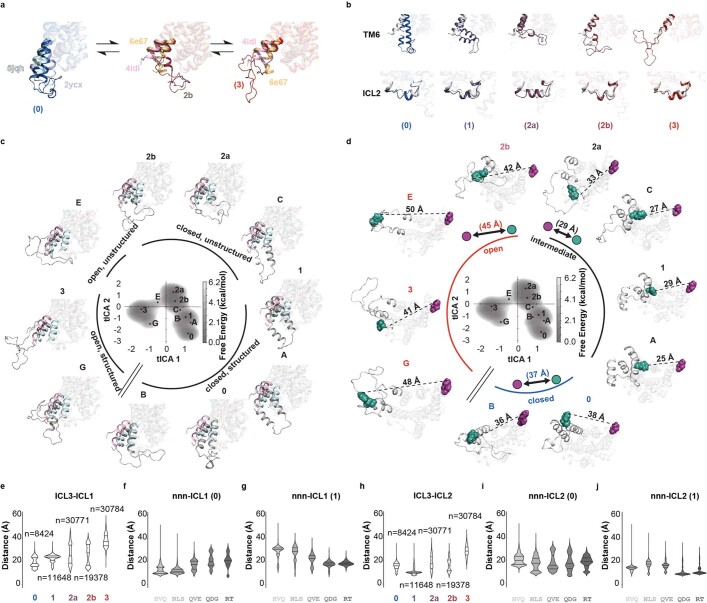
Conformational states of ICL3 are coordinated with the activation states of β_2_AR. **a**. Inactive and active state alignment of MD simulation trajectories. (*Left*) comparison of representative snapshot of conformation 0, with the inactive state structure of β_1_AR used as a starting point for simulations (PDB ID 2ycx)^23^ and the inactive state structure of β_2_AR bound to carazolol and Nb60 (PDB ID 5jqh)^69^ (residues 217—275 shown in opacity, TM6 labeled). Note that the structure of TM5 and TM6 of conformation 0 more closely align with 5jqh than 2ycx. (*Middle and Right*) comparison of the representative snap shot of conformations 2b and 3 with the active state structure of β_2_AR fused to the Gs peptide used as a starting point for simulations (PDB ID 6e67)^24^ and the active state structure of β_2_AR bound to hydroxybenzylisoproterenol and Nb6B9 (PDB ID 4ldl)^21^ (residues 217—275 shown in opacity, TM6 labeled). Note that the intermediate structure 2b more closely aligns with the TM5 of 6e67 than 4ldl. In contrast, the structure of TM6 of conformation 3 and conformation 2b more closely align with 4ldl than 6e67. **b**. Alignment of the representative structures from each cluster in Fig. 2 and the crystal structure of the β_2_AR-G_s_ complex (PDB ID: 3sn6)^17^ (grey). (*Top panel*) ICL3 (residues 236—257) and TM6 (residues 262—299) shown in opacity. *(Bottom panel*) ICL2 (residues 133—150) shown in opacity. **c**. Representative snapshots from molecular dynamics simulations of β_2_AR depicting the conformational landscape of ICL3. (*Center*) - free energy landscape of simulation trajectories from Fig. 2b. tICA 1 and 2 coordinates of representative snapshots are highlighted. Points 0, 1, 2a, 2b, 3 are taken from local free energy minima of their corresponding free energy basins. Points A, B, C, E, G are taken from the sampling point which has longest distance from its starting point and shortest distance to a nearby state on the free energy landscape. Structural snapshots derived from each of these points (grey) are aligned to both an inactive structure of β_2_AR bound to the drug carazolol and Nb60 (PDB ID 5jqh, cerulean)^69^ and an active structure of β_2_AR bound to the agonist hydroxybenzylisoproterenol and Nb6B9 (PDB ID 4ldl, pink)^21^. Residues TM5.56-TM6.37 are shown with opacity. tICA 1 appears to correspond with how open (negative values) or closed (positive values) ICL3 is over the receptor. tICA 2 appears to correspond with how structured/helical ICL3 is at its N- and C- termini - where it bridges TM5 and TM6 respectively – with higher (positive) values reflecting higher disorder at each end. **d**. Simulation trajectories correlate with FRET sensor readout. Center free energy landscape and states (0-3; A-E) taken from (**c**). Structural snapshots derived from each point are also derived from (**c**), but are shown from the point of view of the cytoplasmic face of the receptor. Residue 258 (green) and 340 (magenta; the last residue simulated in the receptor) are shown as spheres, with the distance (Å) between these residues in each structure labeled. Structures were grouped based on these distances (closed, intermediate and open). Average distance for each of the groups is indicated on the circle diagram. **e**. Distance between ICL3 and ICL1 for simulation trajectories in each highlighted cluster. **f**. Distance between segments of ICL3 (residues 241—254 analyzed in segments of 2-3 residues) and ICL1 for simulation trajectories in cluster 0 (n = 8424 MD snapshots for each violin) or **g**. intermediate cluster 1 (n = 11648 MD snapshots for each violin; reproduction of Fig. 2c for comparative purposes). **h—j** Distance distributions between ICL3 and ICL2. Lines represent quartiles of each data set. Source data

Although our sensor reveals discrete conformations of ICL3, corresponding with the activation state of the receptor, it does not resolve their structural compositions. To map the conformational landscape of ICL3 with enhanced molecular detail, we performed extensive (22 µs) all-atom molecular dynamics simulations of β_2_AR bound to the agonist isoproterenol in a multi-lipid membrane bilayer mimicking cell membrane^22^ (Supplementary Fig. 3). To exhaustively sample the potential conformational landscape of ICL3, our simulations started with various inactive and active structural states of β-adrenergic receptors with ICL3 modelled in (Supplementary Fig. 4). For the inactive state starting point, we used homology modelling, fitting β_2_AR to a structure of β_1_AR^23^ (PDB ID: 2YCX). In this structure, transmembrane helix 6 is pointed in towards the cytoplasmic cavity, overlapping with the binding site of the Gα_s_ C terminus (Supplementary Fig. 5). For active state starting points, we modelled ICL3 with various starting poses into an agonist- and-effector-fused structure of β_2_AR^24,25^ (PDB ID: 6E67) (Methods). Simulation trajectories from each starting pose were aggregated and analysed using Markov state modelling, with additional simulation trajectories generated from one of the starting poses to capture transition points between states (Supplementary Table 2, model D). The aggregated simulation data reveal substates that span a continuum from ‘closed’ ICL3 states that occlude the intracellular cavity, to ‘open’ states that enable access to this cavity (Fig. 2a, 0 to 3). Across the continuum of states, the overall architecture of the simulated receptor aligned well with structurally determined inactive and active states of β_2_AR, with the receptor displaying hallmarks of activation as it transitioned from closed to open states (Extended Data Fig. 5a,b). These substates span a shallow free energy landscape (Fig. 2b) with reversible transitions observed in the molecular dynamics simulation trajectories (Extended Data Fig. 5c). As ICL3 transitions from closed to open states, the distance between L258 and the C terminus of the receptor correlates with our FRET sensor readout (Fig. 1), providing structural context for the three states that we sampled with the FRET-based technique (Extended Data Fig. 5d). We propose that agonist binding transitions ICL3 from inactive closed states that block the G protein-binding site (approximately 37 Å apart, low FRET) to intermediate states, where the receptor shows structural hallmarks of activation, but the cytoplasmic cavity is still occluded by ICL3 (approximately 29 Å apart, high FRET). We infer that Nb6B9 or G_s_ peptide binding then biases ICL3 conformation away from the cytoplasmic cavity of the receptor to open states that are amenable for signalling (approximately 43 Å apart, low FRET).

**Extended Data Fig. 6:**
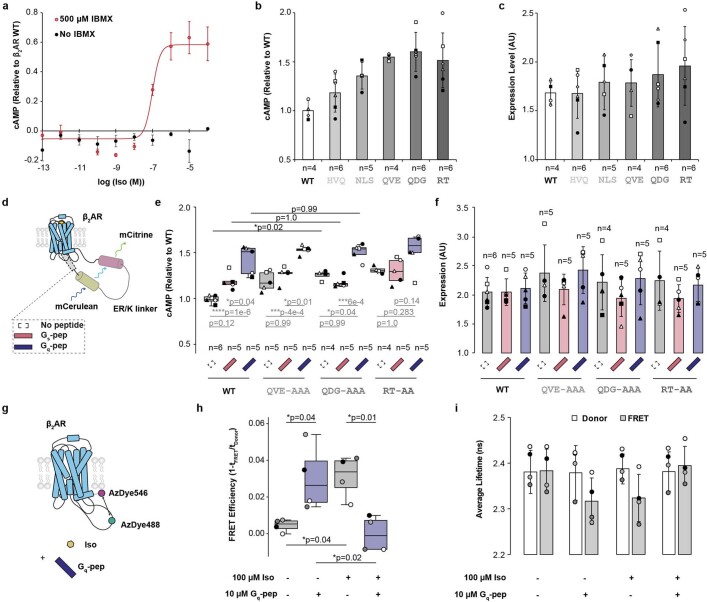
Receptor priming by G peptide involves modulation of ICL3’s conformational ensemble. **a**. cAMP accumulation from endogenous adrenergic receptors in HEK 293T cells. Graph shows cAMP levels in untransfected cells relative to cells transfected with β_2_AR WT (Fig. 2g, Black, 1.60e5 luminescence counts, Fig. 4e, Red, 1.61e5 luminescence counts). Points are mean ± standard deviation of three independent experiments, and curve is the fit of the mean data (Methods). At the concentration of Isoproterenol used for single-concentration assays (10 µM), cAMP generated from endogenous receptors was only detected in the presence of 500 µM IBMX. **b-c**. β_2_AR WT and mutant receptor expression levels for all biological replicates in Fig. 2g. **b**. Figure 2g reproduced with different symbols for each sample (n = biologically independent samples from 6 experiments). **c**. Expression levels of β_2_AR mutants for biological replicates in A shown with matched symbols. Receptors have a fused C-terminal mCerulean tag, which is used to assess the amount of β_2_AR expressed on cells for cAMP experiments. Expression level (Y-axis) represents the mCerulean fluorescence (ex430/em475) divided by cell-concentration dependent cell scattering (ex430/em450). Bars indicate mean ± standard deviation. **d**. Schematic of fusion constructs. Receptor and G_s_- or G_q_-peptide are fused through a 10 nm ER/K α-helical linker, which fixes the local concentration of the peptide at the receptor to ~10 μM. **e**. cAMP accumulation downstream of β2AR-peptide fusion constructs at saturating concentrations of Isoproterenol (10 µM). The three β2AR ICL3 alanine mutants assessed (QVE-AAA, QDG-AAA, RT-AA) have the highest ICL1 distance constraints in molecular dynamics simulations (Fig. 2d), as well as the greatest release of *closed* state mediated auto-inhibition, as assessed by cAMP accumulation (Fig. 2g). n indicates independent biological samples from 6 experiments. Box edges represent the 1st and 3rd quartiles of the data, centerline represents median, whiskers represent outlying points within 1.5x the interquartile range of the data. Points represent independent experiments, with point geometry indicating biological replicates. **f**. Expression levels of β2AR mutants. Y-axis refers to mCerulean fluorescence (ex430/em475) divided by cell-concentration dependent cell scattering (ex430/em450). Bars indicate mean ± standard deviation. Points indicate independent biological samples, with symbols corresponding with (**e**). **g**. Schematic of β_2_AR ICL3 FRET sensor (Fig. 1) treated in presence (+) or absence (−) of agonist Isoproterenol and/or G_q_-pep. **h**. FRET Efficiency for sensor shown in (**g**). Box edges represent the 1st and 3rd quartiles of the data, centerline represents median, whiskers represent outlying points within 1.5x the interquartile range of the data. Points represent 4 independent experiments, with shade indicating replicates. **i**. Source data for FRET Efficiency shown in (**h**). FRET Efficiency is defined as 1-*τ*_FRET_/*τ*_Donor_, where *τ*_FRET_ is the average lifetime of the FRET sensor (FRET, grey bars) and *τ*_Donor_ is the average lifetime of a 488-only labeled control sample (Donor, white bars). Bars indicate mean ± standard deviation from 4 independent experiments. Statistical comparisons shown are from a two-way ANOVA ((**e**): Factor 1 (G peptide treatment): F = 51.7, p = 1.4e-12, Factor 2 (ICL3 Mutation): F = 7.2, p = 4e-4, Factor 1* Factor 2: F = 2.1, p = 0.07, 47 degrees of freedom; (**h**): Factor 1 (G_q_-pep treatment): F = 0.2, p = 0.66, Factor 2 (Iso treatment): F = 0.1, p = 0.75, Factor 1* Factor 2: F = 22.9, p = 4e-4) followed by Tukey’s post-hoc significance test where **** indicates *p* < 0.0001, *** indicates p < 0.001, ** indicates *p* < 0.01, * indicates *p* < 0.05, and NS indicates *p* ≥ 0.05. Source data

To test this model, we sought to interrogate intramolecular interactions that putatively stabilize distinct ICL3 substates. Consistent with the predicted intrinsic disorder of the ICL3 region, we were unable to determine any such persistent non-covalent interactions in our simulation data. Nonetheless, intrinsically disordered regions maintain structural and conformational constraints relative to other regions of the protein when forming intramolecular interactions^26^. We observed that in the closed and intermediate substates, in which ICL3 occluded the receptor cytoplasmic cavity (0 and 1), ICL3 had a narrow distance distribution with ICL1 located on the opposing face of the receptor (Fig. 2c and Extended Data Fig. 5e). To further delineate critical distance constraints that could be consequential for stabilizing closed and intermediate states, we analysed segments of two to three amino acids previously shown to be critical for the function of allosteric modulators derived from the β_2_AR ICL3 that were shown to enhance G protein signalling^27^. We found the distance between ICL1 and these C-terminal residues in ICL3 to be shorter than the distance between ICL1 and more N-terminal residues in ICL3 (Fig. 2d). To examine the functional significance of this observation, we mutagenized a set of highly constrained residues (QDG/AAA) in our ICL3 conformational sensor. Overall, the QDG/AAA mutation decreased fluorescence lifetime (Fig. 2e, wild type versus QDG/AAA). The decrease in lifetime observed upon addition of agonist (Fig. 2e, wild type, buffer versus isoproterenol) was muted in the QDG/AAA mutant, suggesting that the mutation destabilizes the closed states of ICL3. Furthermore, the fluorescence lifetime of the QDG/AAA mutant increases upon addition of agonist and G_s_ peptide compared with addition of agonist alone. This suggests that destabilizing the closed states of ICL3 leads to an easier transition to open states that are amenable for effector binding. Consistent with this interpretation, the QDG/AAA mutation increased the strength of G_s_ peptide binding to the receptor relative to both the wild-type receptor and to mutagenesis of a less constrained site in ICL3 (HVQ/AAA), as measured by a FRET sensor that detects agonist-induced receptor–G peptide complex formation^28^ (Fig. 2f). Additionally, disruption of distance-constrained sites in ICL3 resulted in increased receptor activity, as measured by cAMP accumulation (Fig. 2g and Extended Data Fig. 6a–c). Together, these findings suggest that the conformational equilibrium of ICL3 controls intracellular effector access, thereby autoregulating receptor activity.

## ICL3 steers effector-mediated GPCR activation

Both the cognate G_s_ peptide and the non-cognate G_q_ peptide, composed of the equivalent α5 helix of the α-subunit of the G_q_ protein, allosterically activate β_2_AR^29^. We proposed that this phenomenon, termed GPCR priming, leverages allostery between the receptor cytoplasmic cavity and the orthosteric ligand-binding site, where interactions at the cytoplasmic face of the receptor increase the affinity of the agonist at the extracellular surface^30^. Correspondingly, previous studies have demonstrated that truncation of ICL3 ablates allosteric binding between G protein and agonist^31^. We hypothesized that ICL3 mediates GPCR priming by G_s_ and G_q_ peptides. To test this, we fused the G_s_ and G_q_ peptides to β_2_AR through an ER/K linker that maintains equivalent concentrations^32^, and measured the effects of these fusions on receptor signalling. In agreement with previous reports, fusion of the G_s_ and G_q_ peptides augmented cAMP accumulation for the wild-type receptor (Extended Data Fig. 6d–f). We additionally fused the G_s_ and G_q_ peptides to β_2_AR ICL3 mutants that shifted the conformational equilibrium of ICL3 and increased receptor activity (Fig. 2e–g), observing increases in cAMP accumulation with G_q_ peptide fusion (Extended Data Fig. 6e, no peptide versus G_q_ peptide, all mutants). However, fusion of G_s_ or G_q_ peptide to the receptor did not further augment the increased cAMP observed upon mutagenesis of structurally constrained sites in ICL3 (Extended Data Fig. 6e, wild type versus all mutants). The non-additive effects of the G protein peptide fusions and ICL3 mutations suggest that the peptide-induced increases in receptor activation are mediated by the influence of the peptides on the conformational ensemble of ICL3. To further test this, we assessed the effect of the G_q_ peptide on the conformation of ICL3 using our ICL3 FRET sensor (Fig. 1)—the G_q_ peptide alone increased FRET to a similar level to the agonist alone (Extended Data Fig. 8e). The combination of agonist and G_q_ peptide decreased FRET, suggesting that agonist and G_q_ peptide together drive ICL3 to populate open states (low FRET) that are amenable for signalling^29^.

**Fig. 3:**
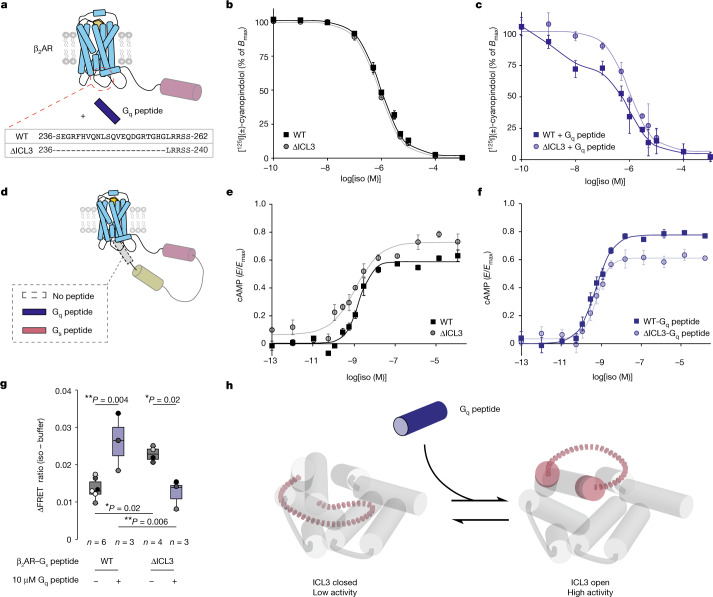
Effector-mediated allosteric activation of the receptor occurs via conformational equilibrium of ICL3. **a**, Schematic of β_2_AR. G_q_ peptide at saturating concentrations (30 µM) is used to allosterically activate the receptor. The ICL3 of β_2_AR is truncated between residues 236 and 257 (∆ICL3). **b**, Competition binding between [^125^I](±)-cyanopindolol and isoproterenol for wild-type β_2_AR and β_2_AR(∆ICL3). *B*_max_ is the maximal amount of specific [^125^I](±)-cyanopindolol binding. **c**, Effect of G_q_ peptide on competition binding between [^125^I](±)-cyanopindolol and isoproterenol for wild-type β_2_AR and β_2_AR(∆ICL3). **d**, Schematic of β_2_AR–G peptide fusion. **e**, Isoproterenol dose–cAMP accumulation response curve for wild-type β_2_AR and β_2_AR(∆ICL3). *E* is the response. **f**, The effect of G_q_ peptide fusion on isoproterenol dose–cAMP accumulation response curve for wild-type β_2_AR and β_2_AR(∆ICL3). In **b**–**f**, data are mean ± s.e.m. of three independent biological experiments and curves are the fit of the mean data (Extended Data Fig. 6b,c and Methods). **g**, Agonist-induced change (∆) in FRET ratio (isoproterenol (100 µM) − buffer) for β_2_AR–G_s_ peptide FRET sensors, comparing the effects of ICL3 truncation (∆ICL3) and G_q_ peptide (10 µM) treatment. Box edges delineate the 1st and 3rd quartiles of the data, the centre line represents median and whiskers represent points within 1.5× the interquartile range. Points represent independent biological samples (*n* indicated on figure). Two-way ANOVA followed by Tukey’s post hoc test; **P < 0.01, *P < 0.05: NS, *P* ≥ 0.05. Factor 1 (G_q_ peptide treatment): *F* = 1.6, *P* = 0.22; factor 2 (wild type versus ∆ICL3): *F* = 0.2, *P* = 0.69; factor 1 × factor 2: *F* = 23.4, *P* = 5.2 × 10^−4^. **h**, Conformational equilibrium model of ICL3-mediated allosteric activation, in which agonist and G_q_ peptide bias the open states of ICL3, without the G_q_ peptide forming stable interactions with the receptor. In turn, the open states relay increased receptor activity. Source data

**Extended Data Fig. 7:**
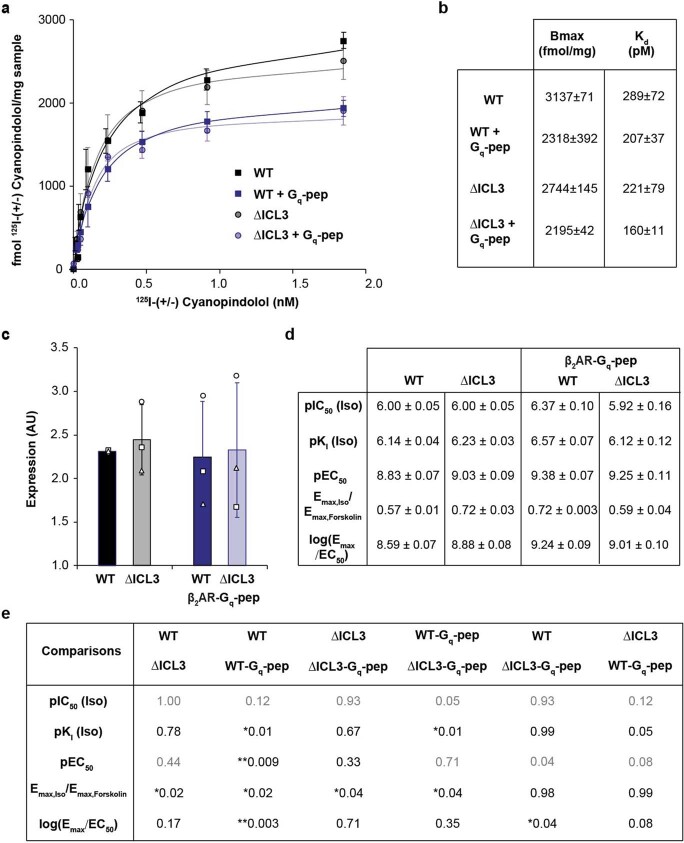
Supporting data for Fig. 3. **a**. ^125^I-Cyanopindolol binding to membranes expressing β_2_AR wild-ype (WT) and β_2_AR ∆ICL3 (∆ICL3 – Fig. 3a). Points represent mean ± standard error of mean from 3 independent experiments. Curve indicates the fit of the mean data (Methods). Binding experiments were done in the presence or absence of G_q_-peptide to assess K_i_ values for the agonist isoproterenol. **b**. Table of fit parameters from binding curves in (**a**) (mean ± standard error of mean). **c**. Expression levels of β_2_AR mutants in cAMP dose response curves (Fig. 3c and e). Y-axis refers to fluorescence of mCerulean fused to receptor C-terminus (ex430/em475) divided by cell-concentration dependent cell scattering (ex430/em450). Bars indicate mean ± standard deviation from 3 independent experiments. Points indicate individual experiments with symbols denoting individual biological replicates. **d**. Table of fit parameters from Isoproterenol competition binding curves (Fig. 3b,c) and cAMP dose response curves (Fig. 3e,f). The agonism index log(E_max_/EC_50_) is derived from individual E_max_ and EC_50_ values^70^. Values indicate mean ± standard error of mean for 3 independent biological experiments. **e**. Table of statistical comparisons of fit parameters. Comparisons shown are from two-way ANOVA (pIC_50_: Factor 1 (WT vs ∆ICL3): F = 5.05, p = 0.05, Factor 2 (G_q_ peptide treatment): F = 2.0, p = 0.20, Factor 1* Factor 2: F = 5.06, p = 0.05); pK_i_: Factor 1: F = 5.6, p = 0.046, Factor 2: F = 4.3, p = 0.07, Factor 1* Factor 2: F = 13.7, p = 6e-3; pEC_50_: Factor 1: F = 0.1, p = 0.72, Factor 2: F = 19.8, p = 2e-3, Factor 1* Factor 2: F = 3.5, p = 0.10; E_max_: Factor 1: F = 0.1, p = 0.79, Factor 2: F = 0.9, p = 0.77, Factor 1* Factor 2: F = 26.3, p = 9e-4; log(E_max_/EC_50_): Factor 1: F = 0.2, p = 0.70, Factor 2: F = 19.8, p = 0.002, Factor 1* Factor 2: F = 8.5, p = 0.02; 8 degrees of freedom for all tests) followed by Tukey’s post-hoc significance test where ** indicates *p* < 0.01 and * indicates *p* < 0.05. Grey indicates an insignificant comparison of factors (*p* ≥ 0.05). Source data

To further investigate whether ICL3 is necessary for β_2_AR priming, we truncated 22 ICL3 amino acids of from β_2_AR (∆ICL3, ∆236–257) (Fig. 3a). Consistent with previous β_2_AR ICL3 mutagenesis studies (Supplementary Table 1), we observed a negligible effect of truncation on agonist binding affinity compared with the wild-type receptor (Fig. 3b and Extended Data Fig. 7). Although the presence of G_q_ peptide increased agonist binding affinity for wild-type β_2_AR, this effect was lost for β_2_AR(∆ICL3) (Fig. 3c and Extended Data Fig. 7d). This same trend was observed in relation to agonist signalling efficacy (log(*E*_max_/EC_50_), where *E*_max_ is the maximal response and EC_50_ is the half-maximal agonist concentration) (Fig. 3e,f and Extended Data Fig. 7d). As an orthogonal measure of the influence of the G_q_ peptide on receptor activation, we evaluated the effect of the G_q_ peptide on β_2_AR–G_s_ peptide coupling using a FRET sensor (Fig. 3d). Treatment with G_q_ peptide enhanced FRET for the wild-type β_2_AR–G_s_ peptide sensor (Fig. 3g). ICL3 truncation alone also increases FRET relative to the wild type, consistent with our alanine mutagenesis experiments (Fig. 2f). The G_q_ peptide did not enhance FRET for the β_2_AR(∆ICL3)–G_s_ peptide sensor, aligning with our results from agonist binding and signalling assays. Viewed through the lens of our conformational equilibrium model (Fig. 1c), our data suggest that the G_q_ peptide allosterically activates the receptor by biasing ICL3 conformation to open and active states (Fig. 3h).

## ICL3 is a determinant of G protein selectivity

**Fig. 4:**
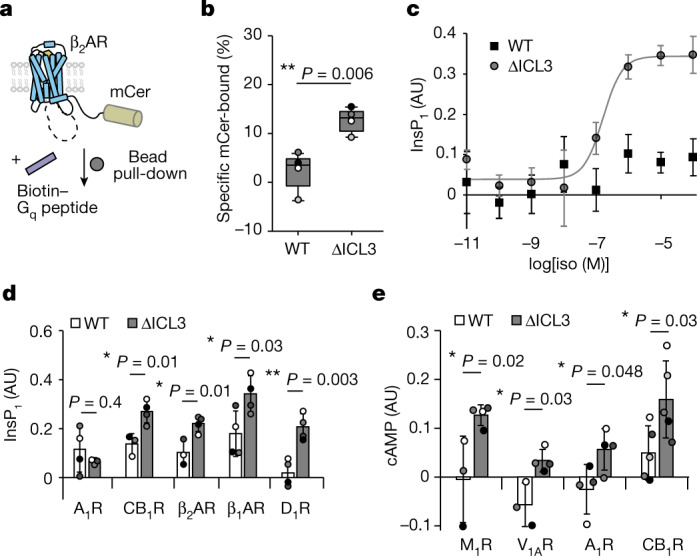
ICL3 autoregulates secondary and non-cognate interactions with GPCRs. **a**, Schematic of the G_q_ peptide pulldown assay. β_2_AR–mCerulean is pulled down onto streptavidin-coated magnetic beads via a N-terminally biotinylated G_q_ peptide. mCer, mCerulean. **b**, G_q_ peptide pulldown measurements comparing wild-type β_2_AR and β_2_AR(∆ICL3) (*t* = −4.2, 7 d.f.). Box edges delineate the 1st and 3rd quartiles of the data, the centre line represents the median and whiskers represent outlying points within 1.5× the interquartile range of the data. Points represent four independent experiments. **c**, Isoproterenol dose–InsP_1_ accumulation response curve for wild-type β_2_AR and β_2_AR(∆ICL3). Data are mean ± s.d. of 4 biological replicates and the curve is the fit of the mean data (EC_50_ = 10 nM). AU, arbitrary units. **d**, InsP_1_ accumulation at saturating agonist concentrations (Supplementary Table 3) for G_i_-coupled receptors A_1_R (WT: *n* = 4, ∆ICL3: *n* = 3, *t* = −0.91, 5 d.f.) and CB_1_R (WT: *n* = 3, ∆ICL3: *n* = 4, *t* = 3.87, 5 d.f.) and G_s_-coupled receptors β_1_AR (*n* = 4, *t* = 2.71, 6 d.f.), β_2_AR (WT: *n* = 3, ∆ICL3: *n* = 4, *t* = 3.72, 5 d.f.) and D_1_R (*n* = 4, *t* = 4.86, 6 d.f.). Data are mean ± s.d. of *n* independent biological samples. **e**, cAMP accumulation at saturating agonist concentrations for G_i_-coupled receptors A_1_R (*n* = 4, *t* = 2.47, 6 d.f.) and CB_1_R (*n* = 5, *t* = 2.55, 8 d.f.) and G_q_-coupled receptors M_1_R (WT: *n* = 3, ∆ICL3: *n* = 4, *t* = 2.95, 5 d.f.) and V_1A_R (WT: *n* = 3, ∆ICL3: *n* = 4, *t* = 3.56, 5 d.f.). In **d**,**e**, the left bar represents the wild-type receptor and the right bar represents the ∆ICL3 mutant (Supplementary Table 3). Data are mean ± s.d. of *n* independent biological samples. Points represent biological samples, shaded by experimental replicate. **b**,**d**,**e**, Unpaired two-sided *t*-test comparing wild type and ∆ICL3; **P < 0.01 and *P < 0.05. Source data

**Extended Data Fig. 8:**
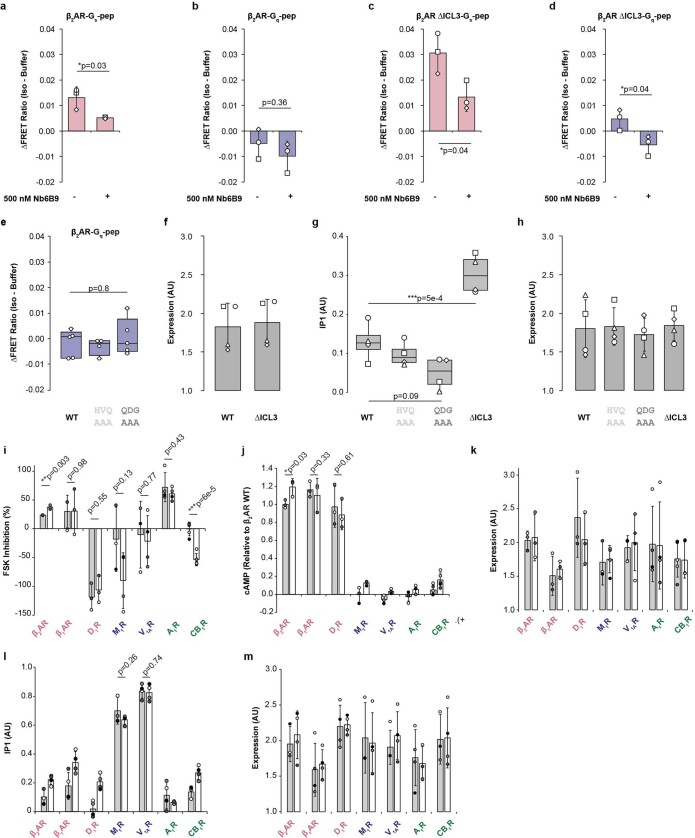
Non-cognate and secondary GPCR-G protein coupling is regulated by ICL3. **(a—d)** Cognate and non-cognate G-peptides bind the receptor at the cytoplasmic cavity. The active state nanobody Nb6B9, which binds β_2_AR tightly at the cytoplasmic cavity, was used to quench agonist-induced changes in FRET (Isoproterenol (100 µM)-Buffer) for sensors linking β_2_AR to either the cognate G_s_ peptide (**a** and **c**) or non-cognate G_q_ peptide (**b** and **d**). ΔICL3 refers to truncation of ICL3 sequence as depicting in Fig. 3a. Bars represent mean ± standard deviation for 3 independent experiments, and symbols represent independent biological replicates. Statistical comparisons shown are from unpaired two-sided t-tests comparing WT and ∆ICL3, where ** indicates *p* < 0.01 and * indicates *p* < 0.05 (**a**: t = 3.26, **b**: t = 1.04, **c**: t = 3.06, **d**: t = 3.10, 4 degrees of freedom for each). (**e-h**) Site-directed mutations in ICL3 alone are not sufficient to alter β_2_AR signaling specificity. **e**. Agonist induced change in FRET ratio (Isoproterenol (100 µM)-Buffer) for β_2_AR-G_q_-peptide FRET sensors. Sensors for the wild-type β_2_AR (WT) (n = 5 biologically independent samples), HVQ-AAA (n = 4), and QDG-AAA (n = 5) mutations in ICL3 showed no significant differences in sensor responses (F = 0.5, p = 0.6, 11 degrees of freedom). **f**. Expression levels of β_2_AR mutants in IP1 dose response curves from 4 independent experiments (Fig. 4c). **g**. IP1 accumulation downstream of β_2_AR mutants (HVQ-AAA, QDG-AAA and ICL3 truncation (ΔICL3 – Fig. 3a)) under saturating Isoproterenol (10 µM) (n = 4 independent experiments). For **e** and **g**, Box edges represent the 1^st^ and 3^rd^ quartiles of the data, centerline represents median, whiskers represent outlying points within 1.5x the interquartile range of the data. Symbols represent independent biological replicates. Statistical comparisons shown are from a one-way ANOVA (F = 26.02, p = 1.5e-5, 12 degrees of freedom) followed by Tukey’s post-hoc significance test where *** indicates *p* < 0.001. **h**. Expression levels of β_2_AR mutants in (C). For **f** and **h**, Y-axis refers to mCerulean fluorescence (ex430/em475) divided by cell-concentration dependent cell scattering (ex430/em450). Bars indicate mean ± standard deviation. Symbols denote independent experiments (n = 4). (**i—m**) ICL3 truncation enhances non-cognate or secondary G protein signaling. Assays were performed at saturating agonist concentrations (Supplementary Table 3). Receptor names are colored by cognate/primary G protein (red – G_s_; blue – G_q_; green – G_i_) **i**. Forskolin inhibition downstream of receptor activation (G_i_ signaling) for G_s_-coupled receptors β_2_AR (n = 3, t = 6.57, 4 degrees of freedom), β_1_AR (n = 3): t = 0.026, 4 degrees of freedom), and D_1_R (WT: n = 3, t = 0.64, 5 degrees of freedom); G_q_-coupled receptors M_1_R (WT: n = 3, ∆ICL3: n = 4 t = −1.83, 5 degrees of freedom) and V_1A_R (WT: n = 3, ∆ICL3: n = 4, t = −0.3, 5 degrees of freedom); and G_i_-coupled receptors A_1_R (n = 4, t = −0.83, 6 degrees of freedom) and CB_1_R (n = 4, t = −7.54, 8 degrees of freedom) **j**. cAMP accumulation following receptor activation (G_s_ signaling) for G_s_-coupled receptors β_2_AR (n = 3, t = 3.24, 4 degrees of freedom), β_1_AR (WT: n = 3, ∆ICl3: n = 4, t = −1.07, 5 degrees of freedom), D_1_R (n = 3, t = −0.55, 4 degrees of freedom); G_i_-coupled receptors A_1_R (n = 3) and CB_1_R (n = 5); and G_q_-coupled receptors M_1_R (WT: n = 3, ∆ICL3: n = 4)) and V_1A_R (WT: n = 3, ∆ICL3: n = 4). **k**. Corresponding expression level of wild-type receptors (WT, grey bars) and receptor ICL3 truncation mutants (∆ICL3, white bars) for (**i**) Forskolin inhibition and (**j**) cAMP accumulation**. l**. IP_1_ accumulation following receptor activation (G_q_ signaling) for G_s_-coupled receptors β_1_AR (n = 4), β_2_AR (WT: n = 3, ∆ICL3: n = 4), and D_1_R (n = 4); G_q_-coupled receptors M_1_R (WT: n = 3, ∆ICL3: n = 4, t = −1.26, 5 degrees of freedom) and V_1A_R (n = 4, t = −0.35, 6 degrees of freedom); and G_i_-coupled receptors A_1_R (n = 3) and CB_1_R (WT: n = 3, ∆ICL3: n = 4). **m**. Corresponding expression level of wild-type receptors (WT, grey bars) and receptor ICL3 truncation mutants (∆ICL3, white bars) for (**l**) IP_1_ accumulation. Statistical comparisons shown in (**I**,**j** and **l**) are from unpaired t-tests comparing WT and ∆ICL3, where *** indicates *p* < 0.001, ** indicates *p* < 0.01 and * indicates *p* < 0.05. Bars represent mean ± standard deviation of n biological samples (**i**) and (**j**). Shaded circles represent independent experiments. Source data

The G_q_ peptide both unable to prime activation of β_2_AR(∆ICL3) and appeared to decrease G_s_ peptide coupling to this mutant (Fig. 3g). On the basis of this result, we hypothesized that in the absence of ICL3, the G_q_ peptide competitively inhibits cognate G_s_ coupling, leading to suppression of G_s_ signalling. To test this, we first addressed the β_2_AR–G peptide interaction interfaces using Nb6B9, whose receptor-binding interface overlaps with the G_s_-binding site^21^. As expected, Nb6B9 significantly quenched FRET for the wild-type β_2_AR–G_s_ peptide FRET sensor (Extended Data Fig. 8a). For β_2_AR(∆ICL3), Nb6B9 quenched the interactions of both the G_s_ and G_q_ peptides with the receptor (Extended Data Fig. 8c,d). A receptor-pulldown approach (Fig. 4a) demonstrated that β_2_AR(∆ICL3) enhanced the receptor interaction of the G_q_ peptide relative to the wild type (Fig. 4b). Further, β_2_AR(∆ICL3) displayed an agonist dose-dependent increase in inositol monophosphate (InsP_1_) accumulation that is characteristic of G_q_ activation (Fig. 4c, EC_50_ ≈ 100 nM). These data suggest that both cognate and non-cognate G peptides engage the cytosolic cavity of the receptor in the absence of ICL3. β_2_AR coupling to G_q_ is dependent on complete removal of ICL3, as alanine mutations to ICL3 that increased receptor activity did not recapitulate the increased G_q_ peptide interaction strength (Extended Data Fig. 8e) or InsP_1_ accumulation (Extended Data Fig. 8g). Thus, removal of ICL3 from β_2_AR enables a weakly associating G protein to functionally couple to the receptor, increasing G protein signalling promiscuity.

To broaden our insights from β_2_AR to other GPCRs, we removed ICL3 from six other receptors, truncating each receptor at similar ICL3 positions relative to the fifth and sixth transmembrane domains (Supplementary Table 3). Given that these receptors signal primarily through the G_s_ pathway (β_1_-adrenergic receptor (β_1_AR) and D_1_ dopaminergic receptor (D_1_R)), the G_i_ pathway (A_1_ adenosine receptor (A_1_R) and cannabinoid CB_1_ receptor (CB_1_R)) or the G_q_ pathway (the M_1_ muscarinic acetylcholine receptor (M_1_R) and vasopressin V_1A_ receptor (V_1A_R)), we measured the second messenger flux at saturating agonist concentrations for all three pathways. We observed an increase in non-cognate or secondary G protein signalling through G_q_ (InsP_1_) or G_s_ (cAMP) for all receptors tested, except for A_1_R–G_q_ (Fig. 4d,e). However, ICL3 truncation augmented cognate pathway signalling only for β_2_AR (Extended Data Fig. 8i–m); we observed either no change (β_1_AR, D_1_R, M_1_R, A_1_R and V_1A_R) or decreases (CB_1_R) in the suppression of forskolin-stimulated cAMP responses for all receptors tested, except for β_2_AR (Extended Data Fig. 8i). Although agonist-stimulated inhibition of the forskolin response is an established measure of G_i_ activation, we speculate that crosstalk with our observations for G_s_-stimulated cAMP accumulation convolutes interpretation of G_i_ signalling. Nonetheless, the observed increases in G_s_ and G_q_ protein signalling promiscuity at saturating agonist concentrations for a diverse sample of GPCRs demonstrates a general role for ICL3 in G protein selectivity.

## ICL3 screening of G protein signalling

**Fig. 5:**
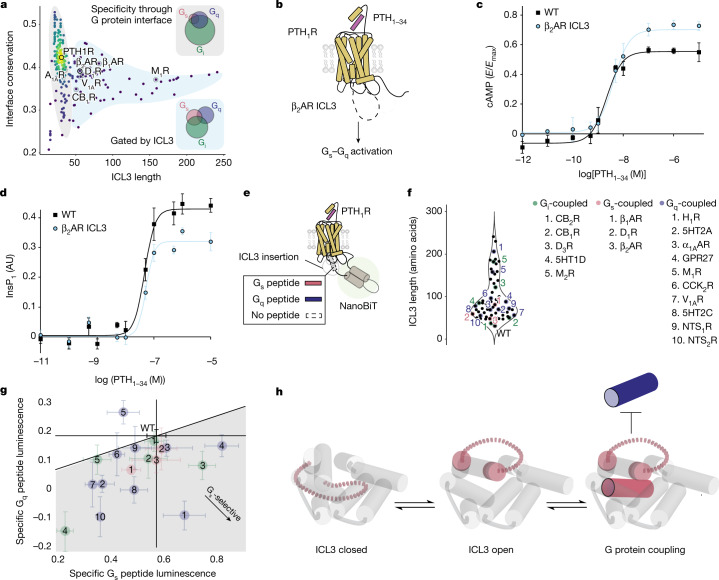
ICL3 is a G protein selectivity filter. **a**, ICL3 length versus conservation of the G protein-binding cavity (*n* = 249 receptors). The colour indicates the density of points from lowest (navy) to highest (yellow). Receptors interrogated in Fig. 4 are highlighted. Insets, Venn diagram of G_s_, G_q_ and G_i_ protein coupling with receptors with short (specificity through G protein interface, *n* = 187) or long (gated by ICL3, *n* = 62) ICL3s. **b**, Schematic of PTH_1_R constructs. The 22-amino acid ICL3 sequence from β_2_AR was inserted into PTH_1_R to create the PTH_1_R–β_2_AR chimera. **c**,**d**, cAMP (**c**) and InsP_1_ (**d**) accumulation downstream of wild-type PTH_1_R (WT) and the PTH_1_R–β_2_AR chimera (β_2_AR ICL3), treated with the agonist PTH_1–34_. Data are mean ± s.e.m. for independent biological samples (*n* = 3 for PTH_1_R–β_2_AR ICL3 chimera in **c**, *n* = 4 for all others) from 4 independent experiments, and curves are the fit of the mean data (Extended Data Fig. 9k and Methods). **e**, Schematic of luciferase complementation reporter assay to compare the effects of ICL3 insertion on PTH_1_R interactions with G_s_ and G_q_ peptides. See Methods and Extended Data Fig. 9n, o for details on data analysis. **f**, Left, plot of ICL3 lengths from the ‘gated by ICL3’ group, with interrogated ICL3 sequences highlighted (*n* = 62). Right, name and numbering scheme for the interrogated ICL3s. αAR, α-adrenergic receptor; 5HT, serotonin receptor; CCKR, cholecystokinin receptor; GPR, probable G protein-coupled receptor; HR, histamine receptor; NTSR, neurotensin receptor. **g**, Specific G_s_ peptide versus G_q_ peptide interaction for each PTH_1_R–ICL3 chimera. Data are mean ± s.e.m. of biological replicates. See Supplementary Table 4 for exact sample size of each individual point. The line indicates proportional effects of ICL3 insertion on G_s_ and G_q_ peptide interactions relative to wild-type PTH_1_R. **h**, ICL3-mediated G protein selectivity. ICL3 is equilibrated between closed and open states. This equilibrium coordinates productive cognate G protein coupling and inhibits coupling to secondary G proteins that are incompatible with the receptor’s G protein interface. Source data

**Extended Data Fig. 9:**
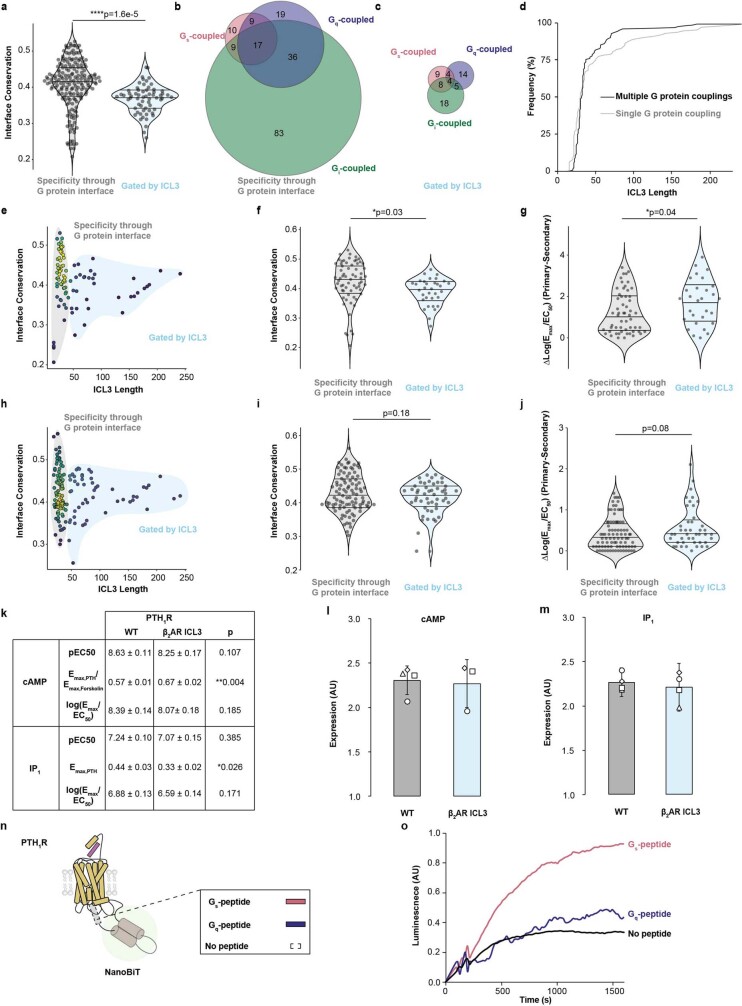
ICL3 length as a G protein selectivity determinant. **a**. Violin plot of interface conservation for receptors in each group shown in Fig. 4a (*Specificity through G protein interface*: n = 183 receptors, *Gated by ICL3*: n = 60 receptors, t = 4.41, 247 degrees of freedom). **b**. Venn diagram of experimental G_s_, G_q_, and G_i_ protein couplings derived from the IUPHAR/BPS *Guide to Pharmacology* database for receptors in the grey region (*Specificity through G protein interface*, n = 187) or **c**. the blue region (*Gated by ICL3*, n = 62) in Fig. 4a. Number of receptors in each overlapping or non-overlapping region is indicated **d**. Cumulative distribution function of ICL3 lengths of receptors with multiple experimentally determined G protein couplings (G_s_, G_i_, G_q_, and/or G_12/13_, n = 132 receptors), or a single experimentally determined G protein coupling (n = 111 receptors). A larger proportion of long ICL3 receptors couple to a single G protein (rightward shift of single G protein coupling plot). **e**. ICL3 length versus conservation of the G protein binding cavity (sequence similarity) for GPCRs with experimentally determined G protein coupling in a high-throughput screening assay (*Specificity through G protein interface*: n = 66 receptors, *Gated by ICL3*: n = 33 receptors)^38,71^. **f**. Violin plots comparing interface conservation (t = 2.21, 97 degrees of freedom) and **g**. G protein selectivity (*Specificity through G protein interface*: n = 56 receptors, *Gated by ICL3*: n = 29 receptors t = −2.11, 83 degrees of freedom) for receptors in E. G protein selectivity is determined by the largest G protein subtype log(E_max_/EC_50_) value (primary) and subtracting the second-largest value (secondary). **h—j**. Repeat of ICL3 length versus interface conservation using data from a second screening assay^37^ (*Specificity through G protein interface*: n = 99 receptors, *Gated by ICL3*: n = 51 receptors, I: t = 1.35, 148 degrees of freedom; J: *Specificity through G protein interface*: n = 88 receptors, *Gated by ICL3*: n = 44 receptors, t = −1.74, 130 degrees of freedom). **k**. Table of fit parameters from dose-response curves in Fig. 5b,c. Values reflect mean ± standard error of mean of biological samples from 4 independent experiments (n = 3 for PTH_1_R-β_2_AR ICL3 Chimera cAMP, n = 4 for all others; cAMP pEC_50_: t = 1.96, 5 degrees of freedom; cAMP E_max_: t = 4.91, 5 degrees of freedom; cAMP log(E_max_/EC_50_): t = −1.53, 5 degrees of freedom; IP1 pEC_50_: t = 0.94, 6 degrees of freedom, IP1 E_max_: t = −2.95, 6 degrees of freedom; IP1 log(E_max_/EC_50_): t = −1.55, 6 degrees of freedom). Statistical comparisons shown in (**a,f-g,i—k**) are from unpaired two-sided t-tests, where * indicates *p* < 0.05, ** indicates *p* < 0.01, and **** indicates *p* < 1e-4. **l**. Expression level of receptor mutants from Fig, 5b and **m**. Figure 5c. Bars represent mean ± standard deviation of biological replicates. Symbols represent independent biological replicates (n = 3 for PTH_1_R-β_2_AR ICL3 Chimera cAMP, n = 4 for all others). **n**. Schematic of luciferase complementation reporter assay to compare the effects of ICL3 insertion on PTH_1_R-G_s_- or G_q_-peptide coupling (see Methods). **o**. Representative time courses of PTH_1_R wild-type. For each data point in Fig. 5g, a time course is collected for a PTH_1_R-G_s_-peptide sensor, PTH_1_R-G_q_-peptide sensor, and a PTH1R No peptide control sensor simultaneously. The plateau of the luminescence value is subtracted from the plateau of the control sensor to represent the specific PTH_1_R-G_s_-and PTH_1_R-G_q_-peptide s. Source data

We sought to contrast the effect of ICL3 on G protein subtype selectivity relative to a more established determinant of G protein selectivity, the amino acid composition of the structurally resolved receptor–G protein binding interface^33^. Individual interface residues of the receptor can either positively and negatively select for G protein interactions, depending on their compatibility with a given G protein subtype. To quantify the cumulative effect of these interface residues, we grouped G_s_-, G_q_- and G_i_-coupled receptors by their primary G protein signalling pathway, with their coupling determined from evidence in the literature, and computed the average sequence similarity of all of their interface residues^34–36^ (Fig. 5a, interface conservation). Given the sequence divergence of ICL3 across receptor subfamilies^4^, we compared interface conservation with ICL3 length (Fig. 5a). We observe two different regimes demarcated by ICL3 length (46 amino acids, Fig. 5a). Receptors with short ICL3s (grey region) have a broad distribution of interface conservation, whereas receptors in the longer ICL3 group (blue region) have narrower and—on average—lower interface conservation (Extended Data Fig. 9a). Furthermore, receptors in the short-ICL3 group exhibit greater overlap in G protein signalling pathways than the long-ICL3 group, in which receptors prefer a single G protein (Fig. 5a and Extended Data Fig. 9b–d). These trends were consistent when we performed the same analysis on subsets of GPCRs, with G protein couplings determined comparatively and quantitatively by high-throughput assays^37,38^ (Extended Data Fig. 9e–j).

The trend of more selective G protein coupling in long-ICL3 receptors, despite the moderate sequence conservation within the established receptor–G protein interface, suggests an important role for ICL3 in G protein selectivity. Our experimental measurements of enhanced signalling promiscuity upon ICL3 truncation align with this observation. Receptors with long (β_2_AR, M_1_R, CB_1_R, V_1A_R, β_1_AR and D_1_R) but not short (A_1_R) ICL3s require this region to maintain G protein selectivity (Fig. 4d,e). To further examine whether a substantial ICL3 length can aid in determining the specificity of G protein signalling, we grafted the β_2_AR ICL3 into the parathyroid hormone 1 receptor (PTH_1_R), a receptor with a short ICL3 that couples primarily to G_s_ and secondarily to G_q_ (Fig. 5b). The PTH_1_R–β_2_AR ICL3 chimeric receptor displays an increased cAMP *E*_max_ (Fig. 5c and Extended Data Fig. 9k) and a decreased InsP_1_ *E*_max_ (Fig. 5d and Extended Data Fig. 9k) relative to wild-type PTH_1_R. However, the efficacy of the agonist parathyroid hormone (PTH_1–34_) (log(*E*_max_/EC_50_)) proportionally decreased for the chimera relative to wild-type PTH_1_R for both cAMP (G_s_) and InsP_1_ (G_q_), albeit not statistically significantly (Extended Data Fig. 9k). Nonetheless, the opposing effects on maximal response for each pathway suggest a role for the β_2_AR ICL3 in enhancing signalling specificity for G proteins that are more compatible with the receptor’s G protein interface at saturating agonist concentrations.

We propose that this property extends to the ICL3s of other receptors, where longer ICL3s ‘buffer’ interactions that are less compatible with the receptor to reinforce selectivity for cognate G proteins. To test this idea, we developed a luciferase complementation reporter assay to compare G_s_ and G_q_ peptide interactions with agonist-stimulated PTH_1_R containing insertions of different receptor derived ICL3s (Fig. 5e). The luciferase signal for the wild-type PTH_1_R–G_s_ peptide interaction in this assay format is stronger than for the wild-type PTH_1_R–G_q_ peptide interaction, recapitulating the established G protein signalling preferences of PTH_1_R^39^ (Extended Data Fig. 9o). We used a panel of ICL3 sequences spanning a range of ICL3 lengths and host receptor–G protein coupling preferences (Fig. 5f and Supplementary Table 4). As expected, insertion of a short ICL3 (six amino acids) into PTH_1_R results in minimal changes in G_s_ and G_q_ peptide interactions relative to wild-type PTH_1_R (Fig. 5g, green 1; cannabinoid CB_2_ receptor (CB_2_R)). By contrast, a longer insertion (17 amino acids) from a receptor in the same subfamily (Fig. 5g, green 2; CB_1_R) has a larger effect on the interactions with both G_s_ and G_q_. In general, most insertions (72%) decreased the interactions with both G_s_ and G_q_ (Fig. 5g and Supplementary Table 4), consistent with our model of ICL3 gating access to the cytosolic cavity of the receptor (Fig. 1e). Of note, all insertions apart from M_1_R disproportionately decrease G_q_ peptide interactions relative to G_s_ peptide interactions with the receptor, rendering these chimeric receptors more G_s_-selective than the wild-type PTH_1_R. Despite the sequence and structural diversity of the ICL3 region across the GPCR superfamily, these findings reinforce a common role for ICL3 in tuning the specificity of GPCR–G protein interactions.

## Discussion

In the current model of GPCR signalling, the sequence, structure and dynamics of structural elements in the cytosolic pocket of the receptor work in concert to determine the strength of coupling to different G protein subtypes^40^. However, this model does not incorporate potential roles for unstructured regions at the receptor–effector interface in G protein selectivity. Computational, structural and pharmacological approaches across a range of GPCRs suggest that ICL3 provides a positive selection mechanism by facilitating cognate G protein coupling^41,42^ (Supplementary Table 5). Here we demonstrate a complementary negative selection mechanism for ICL3 in tuning G protein coupling selectivity. Specifically, ICL3 buffers weakly coupled receptor–G protein interactions, which are poorly compatible with the cytosolic G protein-binding interface of the receptor, to reinforce cognate G protein coupling. Despite the extensive sequence diversity of ICL3s, our experimental and bioinformatic analyses reinforce a length threshold of approximately 45 amino acids as a simple determinant for gating G protein selectivity.

Using β_2_AR as a model receptor system for mechanistic insights, we propose that ICL3 tunes G protein coupling through its autoregulatory conformational ensemble. We provide experimental and computational evidence for a dynamic equilibrium between closed states of ICL3 that occlude the G protein-binding site and inactivate the receptor, and open states of ICL3 that enable receptor–effector interactions and facilitate receptor activation (Fig. 5h). We demonstrate that modulation of this dynamic equilibrium can tune receptor activity and consequently downstream signalling. Specifically, we show that a native peptide derived from the C terminus of the Gα_q_ subunit biases ICL3 in β_2_AR to open states, priming and thereby enhancing receptor activation and subsequent cAMP accumulation. It should be noted that interpretation of the conformational equilibrium of ICL3 can be influenced by technical limitations of our experimental design. Specifically, effectors binding near labelled sites in the receptor could influence sensor lifetime measurements (Fig. 1). Additionally, truncation of the C terminus of the receptor in the sensor ignores potential roles for this unstructured element in receptor activity^4^. Despite these limitations, our data provide proof of concept for allosteric modulation of receptor activity through ICL3. Corroborating these insights, cell-permeable native peptides derived from receptor ICL sequences, termed pepducins, have been proposed to allosterically modulate target receptors by displacing autoregulatory interactions in the cytoplasmic domain^43^. Given the sequence divergence of ICL3s among even closely related GPCRs and the combined evidence for allosteric modulation through ICL3, our findings provide a conceptual framework for using ICL3 as a receptor-selective allosteric site.

## Methods

### Reagents

Human A_1_R, human β_1_AR, HA-tagged human β_2_AR, murine CB_1_R, human D_1_R, human M_1_R, human PTH_1_R, and human V_1A_R constructs were cloned into a pcDNA5/FRT backbone following standard cloning procedures. SPASM sensor constructs were assembled as previously described with Gly-Ser-Gly repeats between domains (Receptor-4×GSG-mCitrine-4×GSG-10 nm ER/K linker-4×GSG-mCerulean-4×GSG-G peptide)^28^. Receptor-fluorescent protein fusion constructs were separated by a 2×GSG linker. PTH_1_R luciferase complementation reporter constructs had the same basic topology as SPASM sensor constructs with lgBiT/smBiT luciferase fragments (Promega N2014) in place of the FRET acceptor/donor and with an inserted fluorescent protein to track expression level (PTH_1_R-lgBiT-4×GSG-10 nm ER/K linker-4×GSG-TagRFP-3×GSG-smBiT-4×GSG-G peptide). Point mutations to various receptor constructs were made to various constructs using a modified site-directed mutagenesis procedure^44^. Large ICL3 deletions were introduced into receptor constructs using the Q5 site-directed mutagenesis method (New England Biolabs E0554). PTH_1_R insertions were introduced using a BsmBI-v2 based vector assembly method (New England Biolabs E1602). Nb6B9 (Nb6B9-2×GSG-SNAP tag-Flag-2×GSG-6×His) was cloned into a pBiex1 backbone following standard cloning procedures. tRNA/Synthetase plasmid (pIRE4-Azi) was a gift from Irene Coin (Addgene plasmid #105829, RRID:Addgene_105829). Carbamoylcholine chloride (14486) was purchased from Cayman Chemical. Synthetic Arginine 8 Vasopressin (CYFQNCPRG-NH_2_), Spep (DTENIRRVFNDCRDIIQRMHLRQYELL), Qpep (DTENIRFVFAAVKDTILQLNLKEYNLV), Bio-Qpep (N-Biotin-DTENIRFVFAAVKDTILQLNLKEYNLV), Bio-Spep (N-Biotin-DTENIRRVFNDCRDIIQRMHLRQYELL) and PTH_1–34_ (SVSEIQLMHNLGKHLNSMERVEWLRKKLQDVHNF) were purchased from Genscript. 3-Isobutyl-1-methylxanthine (IBMX), alprenolol, ascorbic acid, bovine serum albumin, copper II sulfate, forskolin, isoproterenol (+)-bitartrate salt, lysozyme from chicken egg white, metoprolol tartarate, sodium ascorbate, and X-tremeGENE HP transfection reagent were purchased from Sigma-Aldrich. [^125^I](±)-cyanopindolol (NEX174) was purchased from PerkinElmer. 4-azido-l-phenylalanine (1406), AZDye488-Alkyne (1277), AZDye546-Alkyne (1285), 2-(4-((bis((1-(*tert*-butyl)-1*H*-1,2,3-triazol-4-yl)methyl)amino)methyl)-1*H*-1,2,3-triazol-1-yl)acetic acid (BTTAA; 1236), Cy3-Alkyne (TA117), and OG 488-Alkyne (1397) were purchased from Click Chemistry Tools. ATTO488-Alkyne (AD 488-141) was purchased from ATTO-TEC. Polyethyleneimine (PEI) 25 kDa linear polymer was purchased from Polysciences (23966). A 1 mg ml^−1^ solution of PEI was prepared by reconstituting the polymer in endotoxin-free water at 80 °C and passing the solution through a 0.2 µM syringe filter; sterile PEI solution was stored at −20 °C. Ni-NTA agarose (QIA30210) was purchased from Qiagen. PNGase F (P0704) SNAP-Surface 488 (BG-488, S9124S), and Streptavidin-coated magnetic beads (S1420S) were purchased from New England Biolabs. DNAse I (04716728001), fibronectin (F1141) and normal goat serum (G9023) were purchased from Millipore Sigma. anti-HA-Alexa488 (A-21287), formaldehyde (cat# PI28908), and ProLong Diamond Anti-fade Mountant (P36970) were purchased from ThermoFisher. 2-Arachidonylglycerol (1298), dopamine hydrochloride (3548), and *N*^6^-cyclopentyladenosine (1702) were purchased from Tocris. All reagents were reconstituted and stored according to the manufacturer’s specifications.

### Cell culture

HEK 293T Flp-In T-Rex Cells (ThermoFisher, R78007) were cultured in Dulbecco’s modified eagle medium supplemented with 10% (v/v) fetal bovine serum, 20 mM HEPES pH 7.4, and GlutaMAX at 37 °C with 5% CO_2_. Cells were not authenticated, nor were they tested for mycoplasma contamination.

#### Transfection procedures

For membrane preparations, cells were passaged onto 15 cm dishes at ~50% confluence. For each 15 cm dish, 18 µg of DNA, 63 µl of PEI, and 900 µl Opti-MEM were combined, incubated for 15–30 min, and combined with resuspended cells. 4 h after transfection and passaging, the medium was replaced. Receptor transfections (β_2_AR–mCerulean) were incubated for 20 h, G_s_ peptide sensor transfections were incubated for 23 h, and G_q_ peptide sensor transfections were incubated for 26 h.

For second messenger assays, cells were seeded the day prior to transfection at 30–40% confluence. For each well of a 6-well plate, 1 µg DNA, 3 µl X-tremeGENE HP, and 100 µl Opti-MEM were combined, incubated for 15–20 min, and added to seeded wells. As optimal expression time varied for each construct and with cell passage number, transfections were performed at multiple time points between 16–28 h for each second messenger assay.

For PTH_1_R luciferase complementation reporter assays, cells were seeded the day prior to transfection at 25–30% confluence on 12-well plates. For each transfection, 0.5 µg DNA, 1.5 µl X-tremeGENE HP, and 100 µl Opti-MEM were combined, incubated for 15–20 min, and added to seeded wells. For each mutant, PTH_1_R–G_s_ peptide, PTH_1_R–Gq peptide and PTH_1_R–(no peptide) constructs were transfected in parallel. Spep and Qpep constructs were transfected 24 h prior to collection, and no-peptide constructs were transfected 18 h before collection.

#### Stop codon replacement

β_2_AR- nonsense mutants were created by replacing single amino acids with amber (TAG) stop codons. To minimize the influence of conformational changes in the unstructured β_2_AR C-tail on FRET measurements, all β_2_AR constructs used a truncated receptor C terminus (∆350–413). Stop codon replacement transfections were performed following previously described procedures^20^. In brief, 0.5 M Azi was prepared as a fresh stock in 0.5 M NaOH and filtered through a 0.2 µM syringe filter. The fresh Azi stock was added to ~50% confluent 15 cm dishes and incubated for 1–2 h at a final concentration of 0.5 mM. Dishes were co-transfected with β_2_AR nonsense mutant plasmid and pIRE4-Azi at a 1:2 ratio, following the same transfection procedure as membrane preparations. 0.5 mM Azi was added to the changed culture medium. Transfections were incubated 40–44 h prior to collection. Wild-type controls (Extended Data Figs. 2 and  3a–d) were performed in absence of Azi and pIRE4-Azi. Labelling controls (Extended Data Fig. 3e–g) were performed in presence of Azi and pIRE4-Azi.

### Cell imaging

Fibronectin-coated coverslips were prepared by incubating coverslips in 0.01 mg ml^−1^ fibronectin diluted in PBS on a parafilm surface for 1 h at room temperature. After incubation, coverslips were transferred to a cell culture dish. HEK293 cells were seeded at 30% confluency and incubated for 24 h. Cells were transfected following the procedure in ‘Stop codon replacement’, scaled down to a 6-well dish (1 µg DNA, 3.5 µl PEI, and 100 µl Opti-MEM). Following 24 h of expression, culture medium was aspirated from the culture dish, and coverslips were washed 3 times with PBS. Cells were fixed using a solution of 4% formaldehyde diluted in PBS for 10 min at room temperature. Coverslips were washed three times with PBS to remove remaining formaldehyde.

Coverslips mounted using ProLong Diamond Anti-fade Mountant and left at room temperature overnight to cure. For imaging, coverslips were sealed with vaseline/lanolin/paraffin. Images were acquired on a Nikon A1Rsi laser scanning confocal microscope using a 60× oil immersion objective (Nikon).

### Crude cell membrane extracts

#### Procedure

Cells were collected by scraping and pipetting with media and washed twice in phosphate buffered saline (PBS; 10 ml per 15 cm dish) by centrifugation (300*g*, 5 min, room temperature). Cell pellets were resuspended in chilled hypotonic buffer (10 mM HEPES pH 7.4, 1 mM EGTA, 1 mM DTT, 1.5 µg ml^−1^ aprotinin, 1.5 µg ml^−1^ leupeptin, 5 µg ml^−1^ PMSF; 5 ml per confluent 15 cm dish) and incubated for 30 min on ice. Solutions were lysed gently in a chilled Dounce homogenizer (40 strokes). Nuclei and intact cells were separated from the lysate by centrifugation (1,000*g*, 2 min, 4 °C). Lysates were centrifuged (135,000*g*, 25 min, 4 °C). Spun-down lysate was resuspended in assay buffer (20 mM HEPES, 150 mM NaCl, 10 mM KCl, 5 mM MgCl_2_; 1 ml per 15 cm dish of cells). Pellets were then homogenized, first by passing through a 1 ml micropipette 10 times, then passing through a 26-gauge needle 10 times. Lysate was centrifuged again at 135,000*g* following the same procedure and resuspended in assay buffer supplemented with 12% sucrose (w/v; 0.5 ml per 15 cm dish). Pellet homogenization was repeated as before. One-hundred microlitres of a 1:20 dilution of sample in assay buffer was used for analytical fluorescence spectra (Fluoromax 4, Horiba Scientific). Spectra were used to confirm expression level (mCerulean peak emission (excitation 430 nm, 475 nm):optical density emission (excitation 430 nm, emission 450 nm) ratio of 1.0 ± 0.2) and sensor integrity (mCitrine peak emission (excitation 490 nm, emission 525 nm):mCerulean peak emission (excitation 430 nm, emission 475 nm) ratio of 2.0 ± 0.2). Resuspended lysates were aliquoted, flash frozen in liquid nitrogen, and stored at −80 °C.

### Fluorescent dye labelling

#### Click chemistry for radioligand-binding assays

Cell membranes (Extended Data Fig. 3j–m) were prepared following the above procedure, but at a higher cell concentration (1.5 ml hypotonic buffer per 15 cm dish), and without EGTA or DTT in the hypotonic buffer to prevent decreased efficiency of the click reaction. After Dounce homogenization, the following reagents were added to the membrane mixture (final concentrations in parentheses): Cell membranes were prepared following the above procedure, but at a higher cell concentration (1.5 ml hypotonic buffer per 15 cm dish), and without EGTA or DTT in the hypotonic buffer to prevent decreased efficiency of the click reaction. After Dounce homogenization, the following reagents were added to the membrane mixture (final concentrations in parentheses): NaCl (250 mM), KCl (10 mM), MgCl_2_ (5 mM), bovine serum albumin (1 mg ml^−1^) (2 ml final). The dye and labelling reagents were pre-mixed and incubated on ice for 1 min before being added to the lysate mixture at the following final concentrations: 20 µM AZDye488-Alkyne, 250 µM BTTAA, 50 µM copper (ii) sulfate, and 2.5 mM sodium ascorbate. Reactions were incubated with mild shaking (500 rpm) for 30 min at 25 °C.

#### Fluorescence lifetime assays and labelling controls

Cell membranes were prepared following the procedure in ‘Crude cell membrane extracts’ through the first ultracentrifugation step (135,000*g*, 25 min, 4 °C). Pellets were rinsed 3 times with 1 ml assay buffer, and then resuspended in (final concentrations in parentheses): HEPES (20 mM), NaCl (250 mM), KCl (10 mM), MgCl_2_ (5 mM), bovine serum albumin (1 mg ml^−1^), dye (ATTO488-Alkyne, AZDye488-Alkyne, AZDye546-Alkyne, Cy3-Alkyne, and/or OG 488-Alkyne) (5 µM), BTTAA (5 mM), and copper (ii) sulfate (50 mM). Solution was homogenized, first by passing through a 1 ml micropipette 10 times, then passing through a 20-gauge needle 10 times. To initiate the reaction, sodium ascorbate (50 mM) was added to the mixture and mixed by pipetting (2 ml final volume). Reactions were incubated with mild shaking (500 rpm) for 30 min at 25 °C.

#### Azi incorporation controls

Membranes (Extended Data Fig. 3e, SNAP-Tag labelling) were prepared following the procedure in ‘Fluorescence lifetime assay and labelling controls’, with a modified labelling buffer recipe (final concentrations in parentheses): BG-488 (10 µM), DTT (1 mM). No BTTAA or copper (ii) sulfate were added to the mixture. Solution was homogenized, first by passing through a 1 ml micropipette 10 times, then passing through a 20-gauge needle 10 times (500 µl final volume). Membranes were incubated for 30 min at 37 °C.

#### Sample processing

For the three labelling procedures above, aggregated lysate was removed using centrifugation (1,000*g*, 2 min). Lysates were ultracentrifuged as described above for crude cell extracts. After the first ultracentrifugation step, lysate pellets were rinsed 10 times with 1 ml assay buffer. After rinsing, pellets were processed as described above.

To assess expression and labelling efficiency, lysates were diluted 1:10 in optical quartz cuvettes (3-3.30-SOG-3, Starna Cells), and assessed by fluorescence spectroscopy Horiba Fluoromax 4 Fluorometer using the following parameters for the indicated fluorophores: AZDye488/ATTO488/Oregon Green: excitation at 470 nm, emission scan from 500 nm–650 nm; mNeonGreen: samples in optical quartz cuvettes, excitation at 470 nm, emission scan from 495 nm–600 nm; AZDye546: samples in optical quartz cuvettes, excitation at 540 nm, emission scan from 555 nm–620 nm; Cy3: samples in optical quartz cuvettes, excitation at 535 nm, emission scan from 550 nm–620 nm and TagRFP: excitation at 540 nm, emission scan from 565–600 nm. A 1:10 dilution of lysate was also used as an analytical lifetime measurement. Lysates were aliquoted, flash frozen, and stored at −80 °C.

### Radioligand-binding assays

#### Protein content estimation

Membranes were diluted 1:5 in assay buffer. 15 µl of sample was assayed for protein content against a BSA standard (0, 0.4, 0.8, 1.2 mg ml^−1^) in technical triplicates using a DC assay, following the manufacturer’s protocol (BioRad 5000111). DC assay was detected using absorbance (Tecan Spark Plate reader, 750 nm, 9 nm bandwidth, 25 flashes). This protein content estimate was used to calculate membrane *B*_max_ values in fmol mg^−1^.

#### Radiolabelled antagonist-binding assay

Membranes containing expressed β_2_AR constructs were diluted (equivalent of 10,000 mCerulean counts (excitation 430 nm/emission 350 nm) per 200 µl reaction, or ~0.7 µg) in assay buffer supplemented with 30 µM of either Qpep or Spep (depending on condition), 1 mg ml^−1^ Bovine Serum Albumin, 10 mM GTP*γ*S, and 1 mM ascorbic acid and sonicated briefly. Increasing concentrations of [^125^I](±)-cyanopindolol were added to the membrane samples. Non-specific [^125^I](±)-cyanopindolol binding was assessed by repeating the same assay conditions listed above in the presence of 1 mM alprenolol. Reactions were equilibrated on ice for 90 min in 96-well deep well plates. Reactions were transferred to Multiscreen plates (Millipore Sigma MAFCN0B50). Equilibrated reactions were passed through GF/C filters using a vacuum manifold (Millipore Sigma MSVMHTS00) and washed 5 times with 200 µl ice-cold tris-buffered saline (50 mM Tris pH 7.4, 150 mM NaCl). Filters were air dried, removed from plates, and transferred to 75 mm glass tubes. Filter-bound ^125^I was measured by automatic gamma counting (PerkinElmer Wizard^2^). Due to the high concentration of receptor used in the assays, bound [^125^I](±)-cyanopindolol and non-specific [^125^I](±)-cyanopindolol signal for each reaction condition were fit to a saturation-binding curve to obtain the equilibrium dissociation constant (*K*_d_) values for [^125^I](±)-cyanopindolol^45^. Binding assays were performed in technical duplicates. Three biological replicates were collected, with different membrane preparations for each replicate.

#### Competition binding assay

Membrane containing expressed β_2_AR sensors were resuspended following the same procedure as the radiolabelled antagonist-binding assay. Sub-saturating [^125^I](±)-cyanopindolol (50 pM final) and increasing concentrations of isoproterenol were added to the resuspended membrane samples. Non-specific [^125^I](±)-cyanopindolol binding was assessed by measuring 50 pM [^125^I](±)-cyanopindolol in the presence of 1 mM alprenolol. Maximum [^125^I](±)-cyanopindolol signal was assessed using 50 pM [^125^I](±)-cyanopindolol without competing unlabelled ligand. Reactions were equilibrated, washed, and measured following the same procedure listed above for the radioligand antagonist-binding assay. Using the mean dissociation constant (*K*_d_) values for [^125^I](±)-cyanopindolol obtained in the radioligand antagonist-binding assay, bound [^125^I](±)-cyanopindolol and non-specific [^125^I](±)-cyanopindolol signal for each reaction condition were fit to a displacement curve to obtain the equilibrium dissociation constant (IC_50_) values for isoproterenol:

$$Y=({B}_{\max }-{B}_{\min })/(1+{10}^{A-\log ({{\rm{IC}}}_{50})})+{B}_{\min }$$

Where *Y* is the percentage of [^125^I](±)-cyanopindolol bound to the receptor, *B*_max_ is the maximum percentage [^125^I](±)-cyanopindolol bound to the receptor, *B*_min_ is the minimum [^125^I](±)-cyanopindolol bound to the receptor, and *A* is the concentration of isoproterenol. For two-site binding, (WT + Qpep condition), the following model was used:

$$Y=({F}_{1})({B}_{\max }-{B}_{\min })/(1+{10}^{A-\log ({{\rm{IC}}}_{50,1})})+(1-{F}_{1})({B}_{\max }-{B}_{\min })/(1+{10}^{A-\log ({{\rm{IC}}}_{50,2})})+{B}_{\min }$$

Where *F*_1_ is the fraction of sites for affinity site 1, IC_50,1_ is the IC_50_ of affinity site 1, and IC_50,2_ is the IC_50_ of site 2. Weighted averages of these IC_50_ values were used for comparison purposes and inhibition constant (*K*_i_) calculations. IC_50_ values were converted to *K*_i_ values using the Cheng–Prusoff correction^46^ as follows:

$${K}_{{\rm{i}}}={{\rm{IC}}}_{50}/(1+L/{K}_{{\rm{D}}})$$

Where *L* is the concentration of [^125^I](±)-cyanopindolol, and *K*_D_ is the equilibrium dissociation constant for [^125^I](±)-cyanopindolol determined in the antagonist-binding assays. Binding assays were performed in technical duplicates. Three biological replicates were collected with different membrane preparations for each replicate.

### Nanobody expression and purification

Five-hundred millilitres of terrific broth with 100 µg ml^−1^ carbenicillin was inoculated with *Escherichia coli* (JM109 strain) transformed with the Nb6B9 vector at *A*_600_ = 0.05. The culture was grown to *A*_600_ = 0.8 at 37 °C with shaking (180 rpm). The culture was then induced with 0.4 mM IPTG and incubated for 16 h at 16 °C with shaking (180 rpm). The culture was pelleted at 3,000*g* for 15 min. The pellet was then resuspended in 30 ml lysis buffer (20 mM HEPES pH 7.4, 150 mM NaCl, 10 mM imidazole, 10 mM MgCl_2_, 5 mM CaCl_2_, 1 mg ml^−1^ lysozyme, 1 µg ml^−1^ DNAse I, 1 mM DTT, 1 µg ml^−1^ aprotinin/leupeptin, 0.1 µg ml^−1^ PMSF, 1% v/v Triton X-100) and incubated for 30 min at 4 °C on an orbital shaker (100 rpm). The lysate was then sonicated for 10 min (10 s on, 10 s off) and clarified by centrifugation (18,000*g*, 20 min, 4 °C).

A column containing 3 ml Ni-NTA agarose was equilibrated with 15 ml wash buffer (20 mM HEPES pH 7.4, 150 mM NaCl, 10 mM imidazole). Clarified lysate was flowed over the column. Column was washed with 15 ml wash buffer, 15 ml high-salt wash buffer (20 mM HEPES pH 7.4, 500 mM NaCl, 10 mM imidazole), and 15 ml wash buffer. Four total 3 ml elution fractions were collected in wash buffer containing 60 mM imidazole, 120 mM imidazole, 180 mM imidazole, and 240 mM imidazole. All fractions except the 60 mM Imidazole fraction were pooled, concentrated using a 10,000 kDA molecular weight cut-off centrifugal filter (Amicon Ultra-15), and further purified over a Superdex 200 Increase 10/300 GL gel filtration column (GE Healthcare) in size exclusion buffer (20 mM HEPES pH 7.4, 400 mM NaCl). Purity of size exclusion eluate was confirmed via SDS–PAGE. Eluate was concentrated and rebuffered into assay buffer containing 10% w/v Glycerol. Concentration was determined by 280 nm absorbance (Thermo Scientific Nanodrop One). Aliquots were flash frozen and stored at −80 °C.

### Fluorescence measurement assays

#### Fluorescence gel scanning

Ten micrograms (Extended Data Fig. 3e) or 20 µg (Extended Data Fig. 4n) (concentration determined using the procedure in ‘Radioligand-binding assays’, ‘Protein content estimation’) of membranes prepared as described in ‘Crude membrane extracts’ were denatured and deglycosylated with PNGase F. Samples were prepared following the manufacturer’s recommended protocol for denaturing conditions, with the denaturation step scaled to 25 µl and the deglycosylation step scaled to 35 µl. 50 mM DTT and 1× LDS sample buffer were added to the reaction mixture at the end of the procedure. Samples were then separated using 7.5% (Extended Data Fig. 3e) or 10% (Extended Data Fig. 4n) polyacrylamide gels. Gels were scanned for fluorescence (GE Healthcare Typhoon FLA 9500) of AZDye488/ATTO488/Snap Surface Block 488 (excitation 473 nm, long-pass emission filter 510 nm, gain 1,000 V) or AZDye546 (excitation 532 nm, long-pass emission filter 570 nm, gain 1,000V).

#### Time-correlated single photon counting FRET assay

Dye-only controls or membranes containing ICL3 FRET sensors were resuspended in assay buffer containing 1 mM ascorbic acid to an equivalent of ~1.5% of excitation counts/second (0.12 MHz for emission pulses for an 8 MHz excitation pulse) (a final concentration of ~3–4 nM green fluorophore). Ligand (100 µM isoproterenol), G peptide (10 µM), and/or Nb6B9 (0.5 µM) were added for a final reaction volume of 110 µl. For conditions without drug, peptide or nanobody, an equivalent volume of assay buffer was added. Reactions were equilibrated for 5 min prior to reading. One-hundred and five microlitres of each reaction was loaded into an optical quartz cuvette. Measurements were taken by time-correlated single photon counting (DeltaPro, Horiba Scientific) using a 479 nm pulse diode laser and a 515 nm long-pass emission filter. Time-resolved fluorescence decay data were fit to the equation:

$${\rm{Decay}}={\alpha }_{1}{{\rm{e}}}^{-t/{\tau }^{1}}+{\alpha }_{2}{{\rm{e}}}^{-t/{\tau }^{2}}+{\alpha }_{3}{{\rm{e}}}^{-t/{\tau }^{3}}$$

The three-exponential fit was optimized empirically (*χ*^2^ ≈ 1.25, where two-exponential fit *χ*^2^ ≈ 1.4) (Extended Data Fig. 4a–h). Amplitude-weighted average lifetimes (*τ*_avg_) were calculated from the three-exponential decay equation:

$${\tau }_{{\rm{avg}}}=({\alpha }_{1}{\tau }_{1}+{\alpha }_{2}{\tau }_{2}+{\alpha }_{3}{\tau }_{3})/({\alpha }_{1}+{\alpha }_{2}+{\alpha }_{3})$$

Each condition was performed in technical duplicate or triplicate, depending on sensor yield.

#### ∆FRET assay

Membranes containing β_2_AR-SPASM sensors were resuspended in assay buffer based on mCerulean fluorescence (1 × 10^6^ mCerulean counts at 475 nm) and sonicated briefly. For conditions containing Qpep, a final concentration of 10 µM Qpep was added to the membrane mixture following sonication. A final concentration of 100 µM isoproterenol or an equivalent amount of assay buffer was added to each reaction (100 µl final). Reactions were equilibrated for 5 min at 25 °C with shaking (500 rpm). 110 µl of each reaction was loaded into an optical quartz cuvette. Fluorescence spectra (Horiba Fluoromax 4) for mCerulean were acquired for each sample (excitation 430 nm, emission scan 450 nm–600 nm, bandpass 4 nm). The mCitrine (emission 525 nm): mCerulean (emission 475 nm) ratio (FRET ratio) was calculated from each acquired spectra. Each drug–peptide condition was performed in quintuplicate. For each experiment, the ∆FRET metric was calculated by subtracting the average FRET ratio of the buffer only conditions from the average FRET ratio of the isoproterenol-treated conditions.

#### BioSp, BioQp pulldown assay

Membranes containing β_2_AR–mCerulean or β_2_AR-TagRFP (1:10 dilution of frozen membrane aliquots) were resuspended in assay buffer containing 10 mg ml^−1^ Bovine Serum Albumin, 1 mM ascorbic acid and 100 µM isoproterenol and sonicated briefly. Bio-Spep/Bio-Qpep (10 µM final) was added to the reaction mixture (300 µl final) and equilibrated on ice for 30 min. 100 µl of the reaction mixture was removed; an analytical fluorescence spectra of this sample was acquired (mCerulean: Horiba Fluoromax 4, excitation 430 nm, emission scan 450 nm–600 nm, bandpass 4 nm; TagRFP: Tecan Spark Plate Reader, 96-well clear bottom plate, bottom read, excitation at 521 nm, emission scan from 560 nm–650 nm, gain 232) as a measure of the total β_2_AR for a given condition. To the remaining 200 µl, 20 µl of 0.4 mg ml^−1^ streptavidin-coated magnetic beads were added to the reaction mixture, equilibrated for 5 min at ambient temperature, and precipitated using a neodymium disc magnet N52 (20 × 40 mm). Fluorescence spectra was taken of the remaining supernatant in duplicate. Percent mCerulean bound was calculated as the average peak fluorescence count (mCerulean:emission 475 nm, TagRFP: emission 584 nm) of the remaining supernatant samples subtracted from the peak fluorescence counts of the total receptor sample, divided by the peak fluorescence count of the total receptor sample.

### Second messenger assays

#### General procedure

One millilitre of medium was removed from each well containing transfected cells. The remaining volume was used to gently shear and resuspend the cells by pipetting. The cell mixture was centrifuged (3 min, 300*g*) and media was removed using a vacuum manifold. The cell pellet was resuspended in 1 ml of either cAMP assay buffer (PBS with 0.5 mM ascorbic acid, 0.2% (w/v) glucose) or InsP_1_ assay buffer (10 mM HEPES pH 7.4, 1 mM CaCl_2_, 4.2 mM KCl, 145 mM NaCl, 5.5 mM glucose, 50 mM LiCl). The cells were washed once by repeating this procedure.

For Extended Data Fig. 2b, cells were diluted to 2 × 10^6^ ± 5 × 10^5^ cells per ml. Expression level was estimated through TagRFP fluorescence (Horiba Fluoromax 4): excitation 540 nm, emission scan 565–600 nm, bandpass 4 nm.

For all other second messenger assays, expression level and cell density of each condition was estimated through fluorescence. Fluorescence spectra for mCerulean (Horiba Fluoromax 4, excitation 430 nm, emission scan 450–600 nm, bandpass 4 nm) and mCitrine (Horiba Fluoromax 4, excitation 490 nm, emission scan 500–600 nm, bandpass 4 nm) were acquired for each condition. Cells were resuspended at 350,000 ± 30,000 fluorescence counts at a wavelength representing optical density of the sample (excitation 430 nm/emission 450 nm), corresponding with 2 × 10^6^ ± 5 × 10^5^ cells per m^l^. This was confirmed by counting the cells on a haemocytometer (Countess II). The following metrics were assessed for optimal expression: mCerulean peak emission (excitation 430 nm/475 nm):optical density emission (excitation 430 nm/emission 450 nm) of 2.0 ± 0.3 and mCitrine peak emission (excitation 490 nm/emission 525 nm):mCerulean peak emission (excitation 430 nm/emission 475 nm) of 2.0 ± 0.2.

#### cAMP accumulation

Resuspended cells were added 1:1 with a 2× concentration of ligand (10 µl final) into an opaque 384-well flat bottom plate (Greiner Bio-One). For single-concentration experiments, a saturating amount of agonist (10 µM PTH_1–34_ (PTH_1_R), 10 µM 2-arachidonoylglycerol (CB_1_R), 10 µM carbachol (M_1_R), 10 µM dopamine (D_1_R), 10 µM *N*^6^-adenosine (A_1A_R), 100 nM arginine-vasopressin (V_1_R)) was used. For cAMP accumulation experiments with β-ARs, 10 µM isoproterenol was used; for FSK inhibition assays with β-ARs, 100 µM metoprolol was used. For dose–response curves, a saturating concentration of forskolin (10 µM) was included as a control to measure cAMP stimulation independent of the transfected receptor. For experiments comparing multiple receptors measuring non-cognate or secondary G_s_ signalling (Fig. 4e, Extended Data Fig. 8i–k), 500 µM 3-isobutyl-1-methylxanthine (IBMX) was used to inhibit phosphodiesterase activity. For all other experiments, no IBMX was used to minimize cAMP accumulation from endogenous receptors (Extended Data Fig. 6a). For experiments in Fig. 2g and Extended Data Fig. 6b,e, plates were incubated at 37 °C for 10 min to stimulate cAMP production. For all other experiments, plates were incubated at room temperature for 10 min. We found that the room temperature incubation maintained cAMP accumulation levels to equivalent levels as 37 °C incubation and decreased well-to-well variability in the experiments. Reactions were quenched and processed for the cAMP-Glo Assay (Promega) following the manufacturer’s instructions. Luminescence was measured on a Tecan Spark plate reader (500 ms integration, one measurement per well). Data were either normalized to β_2_AR-WT (Figs. 2g and 4e and Extended Data Figs. 6 and  8j), β_2_AR-WT-Nopep (Extended Data Fig. 7d), or to maximum forskolin stimulation (Figs. 3e,f and 5c and Extended Data Figs. 6 and  9k). Dose–response curves were fit to the equation:

$$E=({E}_{\max }-{E}_{\min })/(1+{10}^{\log ({{\rm{IC}}}_{50})-\left.L\right)\times {N}_{{\rm{H}}}})+{E}_{\min }$$

Where *E* is the response, L is the concentration of isoproterenol, *E*_max_ is the response at saturating concentrations of isoproterenol, *E*_min_ is the response in the absence of isoproterenol, EC_50_ is the isoproterenol concentration that gives 50% of the *E*_max_, and N_H_ is the Hill coefficient of the curve.

#### Forskolin inhibition

Experiments were set up as described above. A 1 µM forskolin treatment for each receptor was compared to 1 µM forskolin with saturating concentrations of agonist. All conditions were supplemented with 500 µM IBMX, and a 10 min room temperature stimulation condition was used. Reactions were quenched and processed for the cAMP-Glo Assay (Promega) following the manufacturer’s instructions.

#### InsP_1_

Resuspended cells were added 1:1 with a 2× concentration of ligand (70 µl final) into an opaque 96-well U-bottom plate (Greiner Bio-One). For single-concentration experiments, a saturating amount of ligand (10 µM isoproterenol, 10 µM PTH_1–34_) was used. Plates were incubated at 37 °C for 2 h to stimulate InsP_1_ production. Reactions were quenched and processed for the InsP_1_ HTRF Assay (CisBio), following a protocol modified to achieve a higher signal to noise ratio. Fifteen microlitres of D2-conjugated InsP_1_ resuspended in lysis buffer (Cisbio) and 15 µl of terbium cryptate conjugated anti-InsP_1_ antibody resuspended in lysis buffer (Cisbio) were added to the stimulated cell mixture. Cell lysate was equilibrated for 1 h at ambient temperature with shaking (500 rpm). Reactions were transferred (4 × 20 µl) to a 384-well plate for technical replicates. Fluorescence readings (Flexstation3, Molecular Devices) of acceptor D2 (excitation 340 nm, emission 665 nm, cut-off 630 nm) and donor terbium cryptate (excitation 343 nm, emission 620 nm, cut-off 570 nm) were acquired with a delay of 50 µs and an integration time of 300 µs. FRET ratio for each reading was calculated as the ratio of acceptor emission to donor emission. InsP_1_ signal for a drug and transfection combination was calculated as the average FRET ratio of a given transfection condition without drug treatment subtracted from the average FRET ratio of a of a given transfection condition with drug treatment. Dose–response curves were fit to the same equation as cAMP dose–response curves.

#### Outlier handling

Biological samples with poorly matched cell density or receptor expression levels (desired parameters for collection are indicated in ‘General procedure’) were flagged as potential failed sample replicates. Flagged samples that were outliers (absolute *Z*-score greater than 3) in comparison to other biological replicates were omitted.

### PTH_1_R-luciferase complementation reporter assay

Expressed PTH_1_R-luciferase complementation constructs were vesiculated as previously described, with modifications for smaller sample volume^25^. Media in each well was used to gently shear and resuspend the cells by pipetting. The cell mixture was centrifuged (3 min, 300*g*) and the media was aspirated. Cell pellet was resuspended in 1 ml PBS and centrifuged again as above. Cells were resuspended again in 0.6 ml vesiculation buffer (10 mM HEPES pH 7.4, 150 mM NaCl, 20 mM CaCl_2_, 2 µg ml^−1^ aprotinin, 2 µg ml^−1^ leupeptin, 2 mM *N*-ethylmaleimide) and incubated at 30 °C with shaking at 180 rpm for 2 h. Cellular debris was removed by centrifugation (1,000*g* for 1 min). To collect additional vesicles, debris was resuspended in 0.3 ml PBS, briefly vortexed, and centrifuged again (1,000*g* for 1 min). The ~0.9 ml of combined decanted supernatant was centrifuged one additional time (1,000*g* for 2 min) to better remove cellular debris. Vesicles were collected by centrifugation (3,200*g* for 40 min at 4 °C) and washed in 0.5 ml assay buffer. Centrifugation step was repeated, and vesicles were resuspended in 0.1 ml assay buffer.

Vesicle samples were collected in a 96-well clear bottom plate and assayed for TagRFP fluorescence (Tecan Spark, excitation 521 nm, emission 585 nm, gain 150). For each set of constructs for a given ICL3 insertion (Spep, Qpep, and control), samples were diluted to the lowest TagRFP counts.

Four ICL3 insertion constructs were assayed at a given time (12 total). 45 µl of each sample was transferred to an opaque 96-well flat bottom plate. 45 µl of Nano-Glo Substrate (Promega N1110) diluted 1:50 in assay buffer was added to each well in the new plate. After tapping the plate to collect liquid at the bottom of the well, a kinetic luminescence read was started (500 ms integration, continuous for 40 min) and luminescence signal was tracked. When luminescence signal equilibrated (plateau between 300–350 s), the kinetic read was paused and 10 µM of PTH_1–34_ was added to each well. The plate was tapped 2–3 times to mix, and the kinetic read was resumed. Moving averages were computed for each kinetic trace (3-point averages for 5 min equilibration, 8-point moving averages post-drug treatment). Each kinetic trace was normalized to the last point of the pre-drug equilibration. The maximum luminescence value that appeared stable over time was used for further analysis. Specific Spep and Qpep signals were calculated by subtracting the control value from the Spep value and the Qpep value for a given experiment.

### Molecular dynamics simulations

For maximum sampling of the conformational heterogeneity exhibited by the third intracellular loop of β_2_AR, we used multiple runs of all-atom molecular dynamics simulations using the agonist isoproterenol, as detailed below.

#### Initial state structural models of β_2_AR

β_2_AR with a truncated N terminus (∆1–34) and truncated C terminus (∆341–413) was used for all simulations. We built the ICL3 sequence (228-RQLQKIDKSEGRFHVQNLSQVEQDGRTGHGLRRSSK-263) as an unstructured loop into distinct models of the receptor derived from known structures of β_1_AR and β_2_AR, with the following rationale (Supplementary Table 2, Supplementary Fig. 4):

1. Model A. Published inactive state crystal structures of β_2_AR lack atomic coordinates in the ICL3 region. However, the inactive state crystal structure of thermostabilized wild turkey β_1_AR (PDB ID: 2YCX)^47^ has a truncated, but structurally resolved ICL3 that folds into the receptor’s intracellular cavity. Alignment of this structure with the with the agonist- and G protein bound structure of β_2_AR (PDB ID: 3SN6)^17^ showed that a C-terminal portion of β_1_AR’s ICL3 aligns with the Cα5 helix of the G protein bound β_2_AR (Supplementary Fig. 5). We posited that this is a possible ‘autoregulated’ state of GPCR activity, wherein the ICL3 competitively inhibits G protein binding. Thus, we used the β_1_AR inactive state structure (2YCX) as a template to derive a homology model of β_2_AR in the autoregulated inactive state using SWISS-MODEL software^48^.
2. Model B. Complementing the agonist- and G protein bound structure of β_2_AR is a structure of β_2_AR in complex with a 14-amino acid peptide derived from the Cα5 helix of the G protein (PDB ID: 6E67)^24^. The orientation of the 14-amino-acid peptide is distinct from that of the Cα5 helix of the G protein in the 3SN6 structure, and as posited by Kobilka and colleagues, represents an intermediate state in the G protein activation mechanism.
3. Model C. In our previous work^25^, we performed molecular dynamics simulations of β_2_AR using the atomic coordinates of PDB 6E67 as an initial state. In these simulations, we observed that the C-terminal cap of transmembrane helix 5 transitioning into the ICL3 region unravels. This is distinct from the helical conformation of this region observed in models A and B. Since this could be a part of the transition from the intermediate to the active state, we used this structural model as a starting point.
4. Model D. In our previous work^25^, we also observed that upon removing both a fused T4 lysozyme and an engineered disulfide bond between the receptor and the 14-amino-acid G peptide from the 6E67 structure, the peptide unravelled in our simulations, leaving just one turn of the Gs peptide capping the receptor’s G protein-binding site. As this could represent the movement of ICL3 out of the autoregulated state, we built the C-terminal portion of ICL3 mimicking this structure, with the rest of the loop modelled in an extended conformation.

#### Cell membrane mimicking multi-lipid bilayer

To study the effect of multiple lipids on the GPCR conformation ensemble, we used a mixed lipid bilayer to mimic the cell membrane (Supplementary Fig. 3). The outer leaflet of the membrane bilayer consists of POPC, DOPC, POPE, DOPE, sphingomyelin (Sph), ganglioside (GM3) and cholesterol in the ratio of 20:20:5:5:15:10:25, while the inner leaflet contains POPC, DOPC, POPE, DOPE, POPS, DOPS, phosphatidylinositol 4,5-bisphosphate, and cholesterol in the ratio of 5:5:20:20:8:7:10:2^22^. To further mimick the cell membrane conditions, we neutralized the composite lipid bilayer with 150 mM of CaCl_2_. To obtain a random distribution of lipids in coarse grain simulations, the lipid bilayer was built three times in the same composition given above. After equilibration of each of the three simulation boxes, we performed 10 μs of coarse grain molecular dynamics (CGMD) simulations with Martini2.2 forcefield^49^. The coarse grain lipid bilayer models were converted to all-atom models using the script backward.py from the Martini website^50^. We extracted five different cell membrane lipid configurations, described in detail in our previous work^22^. We then inserted our four initial state models of β_2_AR into these lipid configurations. After elimination of steric clash between the receptor and lipids, we found with one GPCR–lipid bilayer complex for each model A to C and five GPCR–lipid bilayer complexes for model D.

#### All-atom molecular dynamics simulation protocol

Each GPCR–lipid bilayer complex was solvated with water and neutralized with 150 mM of CaCl_2_. The disulfide bonds were built according to the disulfide bonds listed in the 6E67 structure’s template PDB file. The minimization–heating–equilibration–production was carried out as previously described^51–54^. Each GPCR–lipid bilayer complex was minimized and equilibrated using a 50 ns NPT equilibration simulation protocol (constant number of particles, pressure and temperature). Equilibration was performed starting with position restraints placed on the receptor, heavy atoms (C, N, O, S and P) in the ligand, and in the head group of the lipids. The force constant on the position restraints were reduced from 5 to 0 kcal mol^−1^ by a 1 kcal mol^−1^ interval per 10 ns simulation window. The last 10 ns of equilibration simulations were performed with no constraints. Starting from the last frame of the equilibration protocol, we performed 400 ns all-atom molecular dynamics simulation runs with NPT ensemble at 310 K with 2 fs time step using GROMACS with CHARMM36mFF^55^. We stored molecular dynamics snapshots during the molecular dynamics simulations at 20 ps intervals. The non-bond interactions in each simulation were calculated with a cut-off of 12 Å. The particle mesh Ewald method was applied to calculate van der Waals interactions^56^. The temperature was maintained at 310 K using Nose-Hoover thermostat^57^ and pressure at 1 atmosphere using Parrinello–Rahman method^58^.

In one of the simulation runs starting from model D, we observed that the cap of the helix that was blocking the G protein site (Supplementary Fig. 4) left the G protein-binding pocket and transitioned to the fully open state of ICL3. However, when we generated a free energy surface of our combined simulations (see ‘Free energy landscape’), there was no connection between these two states. To enrich the sampling of this rare event, we generated a swarm of simulation trajectories. We extracted three snapshots from the original simulation with transition event at 50 ns, 100 ns and 150 ns (Supplementary Table 2). We then performed a production run for 2 μs from each of these snapshots. Thus, we generated a total of 22 μs of molecular dynamics simulations to analyse the heterogenous conformation ensemble of ICL3.

#### Free energy landscape

In order to describe the global motion of ICL3 in our simulations, we mapped our simulation trajectories onto a free energy landscape using the Markov state model in the software MSMBuilder2 (Version 3.8.0)^59^. The backbone dihedral angles (phi and psi) of the whole GPCR were chosen as order parameters to describe the motion of ICL3. The phi and psi angle matrix was projected into two-dimensional space using time-correlated independent component analysis (tICA) with a lag time of 2 ns, and free energy landscape constructed based on the inverse of the population density. Four major free energy basins were observed on the free energy surface, which were mostly distinguished by the conformation of ICL3. The MinibatchKMean clustering method was applied on all sampling points to distinguish them into distinct clusters^60^. 5 total clusters were generated: one for each free energy basin, and two subclusters for one of the intermediate free energy basins (Fig. 2a,b).

#### Centre of mass distance measurement

To quantify putative structural constraints between ICL3 and other intracellular regions of the receptor, we calculated the distance between the centre of mass of ICL3 (S236–G257) and the centre of mass of either ICL2 (T136–T146) or ICL1 (F61–T66). Distance calculations were performed for each of the five conformation clusters extracted from the free energy landscape. Based on the tight distance distribution observed in cluster 1, we performed additional distance calculations comparing the centre of mass of five individual ICL3 sequence segments (241-HVQ/NLS/QVE/QDG/RT-254) and the centre of mass of either ICL2 or ICL1 for cluster 1.

### Bioinformatics analyses

#### Meta-analysis of ICL3 mutation data

As ICL3 is highly variable in sequence length, we opted to use an N- and C- terminal numbering scheme to keep track of the locations of mutagenized sites, where the N-terminal half of an ICL3 sequence is N1-Nn, and the C-terminal half is Cn-C1, where n is one-half the length of a receptor’s ICL3 sequence. We used TM5.56 as a starting point for the N-terminal sequence numbering to demarcate cytoplasmic exposure of TM5. The same logic applied to TM6.37 for the C-terminal sequence numbering (Extended Data Fig. 1 and Supplementary Tables 1–4).

We included all mutational data (p*K*_d_, pEC_50_, and *E*_max_) that we could find with a wild-type reference point. For p*K*_d_ plots, we only included agonist binding data. To plot the effect of location of mutation versus functional effect, we normalized each ICL3 length to the shortest ICL3 in the dataset (22 amino acids). Each position mutated was assigned the effect of the mutation.

#### ICL3 length versus G protein interface conservation

G protein interface conservation (Fig. 5a and Extended Data Fig. 9a–j) was calculated as the sequence similarity of all amino acids composing the GPCR’s G protein-binding interface. The residues composing this interface were inferred from previous structural alignment and interface mapping^33,34^. Sequence similarity was calculated from four separate GPCR interface alignments, in which receptors were separated based on their primary G protein signalling transduction pathway in the IUPHAR/BPS Guide to Pharmacology database^34,61^.

Interface composition was compared to ICL3 sequence length. The starting position and ending position of ICL3 for each GPCR was determined based on the generic residue numbers of the first and last cytosol-exposed residue in the ICL3 region of B2AR, as determined from crystal structures (PDB ID: 3SN6).

We repeated the analysis for two other datasets assessing GPCR G protein subtype specificity using parallelized high-throughput screening techniques^37,38^. These datasets allow for quantitative comparison different G proteins coupling to a given receptor. To assess if there were high-level differences in G protein selectivity for the short-ICL3 and long-ICL3 groups in these datasets, we compared the highest log(E_max_/EC_50_) value (considered cognate) with the second-highest value (considered secondary) for each receptor. For this analysis, we did not include the receptors that only had a log(E_max_/EC_50_) for a single receptor (Extended Data Fig. 9g,j).

### Statistics

Statistical analyses were performed in RStudio (version 2022.12.0). For experiments comparing two conditions, an unpaired two-sided *t*-test was used. For experiments comparing more than two conditions, analysis of variance was used. One-way ANOVA was used for single level comparisons (for example, effects of mutations), and two-way ANOVA was used for two-level comparisons (for example, effects of mutations and effects of G peptides). To compare between conditions, Tukey’s post hoc test was used. For two-way ANOVA comparisons where the interaction effect was not significant (*P* > 0.05), we did not make individual post hoc comparisons between levels (for example, we would still compare mutation A to mutation B, peptide A to peptide B, but not mutation A compared to peptide B). Statistics were not used to pre-determine sample size for any experiments. Conditions for biological samples (membranes, cells, vesicles) were plated and/or assayed in random order between experimental replicates for all datasets. Investigators were not blinded to group allocation during data collection or analysis, as all data presented are quantitative and no subjective metrics were assessed.

### Software

Fluorescence lifetime data were fit in DAS6 (Horiba). Curve fits were performed in Excel using the Solver add-in. Figures were generated in RStudio (version 2022.12.0) using the ggplot2 package^62^. Image processing was performed in Fiji^63,64^. Molecular structure representations were created using VMD (version 1.9.3)^65^ and Pymol (version 2.0.6)^66^.

### Reporting summary

Further information on research design is available in the Nature Portfolio Reporting Summary linked to this article.

## Online content

Any methods, additional references, Nature Portfolio reporting summaries, source data, extended data, supplementary information, acknowledgements, peer review information; details of author contributions and competing interests; and statements of data and code availability are available at 10.1038/s41586-023-05789-z.

## Supplementary information

Supplementary Information This file contains Supplementary Figs. 1–5, Supplementary Tables 1–5 and references.

Reporting Summary

## Data Availability

Simulation data are stored on the molecular dynamics database for GPCRs (http://GPCRmd.org) under dynamics ID 1247. Receptor structure files 3SN6, 2YCX, 6E67, 5JQH, AND 4LDL were obtained from the Protein Data Bank (https://www.rcsb.org/). G protein coupling data was obtained from the G protein database (https://gproteindb.org/signprot/couplings). Source data are provided with this paper.

## Source data

Source Data Figs. 1–5 and Source Data Extended Data Figs. 3–9
