# Supplementary material for: Autoregulation of GPCR signalling through the third intracellular loop
Source: Nature. 2023 Mar 8;615(7953):734–41. doi: 10.1038/s41586-023-05789-z (PMC10033409; doi:10.1038/s41586-023-05789-z)
Supplement: Supplementary file 1 — This file contains Supplementary Figs. 1–5, Supplementary Tables 1–5 and references. [file 41586_2023_5789_MOESM1_ESM.pdf]

nature portfolio

https://doi.org/10.1038/s41586-023-05789-z

Supplementary information

Autoregulation of GPCR signalling through the third intracellular loop

In the format provided by the authors and unedited

Receptor	Mutation Style	Positions	Insertion	Positions	Ligand Class	pKd,Mut-pKd,WT	Assay	pEC50,Mut-pEC50,WT	Emax,Mut/Emax,WT	Notes	Ref
**5HT2B**	Insertion	N11-C12	**BRIL**		agonist	0.19					73
**5HT2B**	Insertion	N11-C14	**BRIL**		agonist	-0.10					73
**5HT2B**	Insertion	N11-C14	**BRIL**		agonist	0.17					74
**5HT7R (mouse)**	Directed Mutation	C8	G		agonist	0.00	cAMP	0.68	0.33		75
**5HT7R (mouse)**	Directed Mutation	C6	S		agonist	-0.13	cAMP		0.22		75
**AA2AR**	Deletion	N10-C15			agonist	0.40					76
**AA2AR**	ICL3 replacement	N1-C18	**AA1R**		agonist	-0.18	cAMP	0.13	0.47	+GppNhp condition	77
**AA2AR**	Directed Mutation	C17-C16	A,A		antagonist	0.06	cAMP	-0.29	0.81		77
**AA2AR**	ICL3 replacement	C14-C11	**AA1R**		antagonist	-0.03	cAMP	-0.07	0.74		77
**AA2AR**	ICL3 replacement	C14-C7	**AA1R**		antagonist	0.02	cAMP	0.16	1.05		77
**AA2AR**	ICL3 replacement	N16-C18	**AA1R**		antagonist	0.09	cAMP	-0.25	0.97		77
**AA2AR**	Directed Mutation	N1,N3	**AA1R**		antagonist	-0.06	cAMP	0.01	0.80		77
**AA2AR**	Directed Mutation	N12	N		agonist	0.25	cAMP	-0.49	1.24	+GppNhp condition	77
**AA2AR**	Directed Mutation	N14	Q		agonist	-0.07	cAMP	-0.72	1.17	+GppNhp condition	77
**AA2AR**	ICL3 replacement	N1-C2	**AA1R**		agonist	0.36	cAMP	-0.73	0.25	+GppNhp condition	77
**AA2AR**	Insertion	N10-C15	**T_4L**		agonist						78
**AA2AR**	Insertion	N10-C15	**T_4L**		agonist	0.48					79
**ACM1**	Deletion	N10-N22			agonist	0.45	IP3	-0.43	0.53	EC50 value is an average of two given values	80
**ACM1**	ICL3 replacement	N25-C11	**ADRB1 (Turkey)**	N22-C11	agonist	0.27	IP3		0.70	Derived from Table 2 (- PTX)	81
**ACM1**	Deletion	C24-C10			agonist	0.26	IP3	0.36	0.63		80
**ACM1**	Directed Mutation	N5,N7,N12	A,A,K		agonist		IP3	0.14	1.00		80
**ACM1**	Directed Mutation	C9,C7	A,A		agonist		IP3	-0.32	0.69		80
**ACM1**	Deletion	N11-C54			agonist	-0.14	IP3	-0.34	0.85		80
**ACM1**	ICL3 replacement	N(-8)-C3	**ACM2**	N(-8)-C4	antagonist	-0.34	current response		0.01		82
**ACM1**	Insertion	N10-C14	**T_4L**		agonist	2.45				additional mutations: N2Q,N12Q, N110Q	83
**ACM1**	Directed Mutation	N2		A	agonist	-0.24	IP3		0.16		84
**ACM1**	Directed Mutation	N3		A	agonist	-0.16	IP3		0.50		84
**ACM1**	Deletion	C23-C10					IP3		0.71		85
**ACM1**	Deletion	N58-C75					IP3		0.87		85
**ACM1**	Deletion	N56-N65					IP3		1.03		85
**ACM1**	Deletion	N67-N73					IP3		0.87		85
**ACM1**	Deletion	N75-C75					IP3		0.95		85
**ACM1**	Deletion	N46-C58					IP3		0.71		85
**ACM1**	Deletion	N38-C63					IP3		0.85		85
**ACM1**	Deletion	N33-C56					IP3		0.95		85

**ACM1**	Deletion	N11-C54			agonist	-0.23	IP3		0.71		85
**ACM1**	Deletion	N10-C35					IP3		0.89		85
**ACM1**	Deletion	N11-C19			agonist	0.34	IP3		0.87		85
**ACM1**	Deletion	N23-C10			agonist	-0.61	IP3		1.19		85
**ACM1**	Directed Mutation	N(-1),N2,N3		A,A,A			IP3		0.34		86
**ACM1**	Directed Mutation	C8,C6,C2	A,A,A				IP3		0.39		86
**ACM1**	Deletion	N10-C35					IP3		1.40		87
**ACM1**	Deletion	N11-C19					IP3		1.00		87
**ACM1**	Deletion	N23-C10					IP3		1.21		87
**ACM1**	Directed Mutation	C7	A		agonist	-0.10	IP3	-0.72	1.00		88
**ACM1**	Directed Mutation	C6	A		agonist	-0.34	IP3	-1.10	0.46		88
**ACM1**	Directed Mutation	C3	A		agonist	-0.27	IP3	0.00	0.46		88
**ACM1**	Directed Mutation	C6,C3	A,A		agonist	-0.85	IP3	-0.86	0.46		88
**ACM1**	Directed Mutation	C7,C3	A,A		agonist	0.11	IP3	1.52	0.31		88
**ACM1**	Directed Mutation	C7,C6	A,A		agonist	-0.49					88
**ACM1**	Directed Mutation	C7,C6,C3	A,A,A		agonist	-0.23					88
**ACM1 (mouse)**	Insertion	N12-C25	RIR		antagonist	0.01	IP1		0.81		89
**ACM1 (mouse)**	Insertion	N12-C84	RIR		antagonist	0.00	IP1		1.03		89
**ACM1 (mouse)**	Insertion	N12-C67	RIR		antagonist	0.00					89
**ACM1 (mouse)**	Insertion	C69-C25	RIR		antagonist	0.00					89
**ACM2**	ICL3 replacement	N(-7)-C1	**ACM1**	N(-7)-C2	antagonist	0.00					82
**ACM2**	ICL3 replacement	N1-C2	**ACM3**	N1-C3	agonist	-0.88	IP3				3
**ACM2**	ICL3 replacement	N1-N16	**ACM3**	N1-N17	agonist	0.17	IP3				3
**ACM2**	ICL3 replacement	N1-C2	**ACM3**	N1-C3	agonist	-1.13	cAMP inhibition		0.02		90
**ACM2**	ICL3 replacement	N1-N16	**ACM3**	N1-N17	agonist	0.26	cAMP inhibition	-0.65	0.40		90
**ACM2**	ICL3 replacement	N1-C3	**ACM3**		agonist	-0.04	current response		1.74		91
**ACM2**	ICL3 replacement	N1-N21	**ACM3**		agonist	0.13	current response		1.21		91
**ACM2**	ICL3 replacement	N1-N12	**ACM3**		agonist	-0.77	current response		0.44		91
**ACM2**	ICL3 replacement	N13-N21	**ACM3**		agonist	-0.19	current response		1.60		91
**ACM2**	Insertion	N11-C14	**T_4L**		agonist	0.08					92
**ACM2**	Directed Mutation	N3		Y	agonist	0.15	IP3				93
**ACM2**	ICL3 replacement	N1-N11	**ADRB1 (Turkey)**	N1-N11	agonist	0.74			0.70		94
**ACM2**	ICL3 replacement	N1-N15	**ADRB1 (Turkey)**	N1-N16	agonist	0.74			0.70		94
**ACM2**	ICL3 replacement	N1-N25	**ADRB1 (Turkey)**	N1-N26	agonist	0.74	G_o activation		0.91		94
**ACM2**	Directed Mutation	N3	Y		agonist	0.68	IP3				95
**ACM2**	ICL3 replacement	N1-N21	**ACM3**	N1-N21	agonist	0.59	IP3				95
**ACM2**	ICL3 replacement	C29-C(-1)	**ACM3**	C29-C(-1)	agonist	-1.08	IP3				95
**ACM2**	Directed Mutation	C3,C2	A,A		agonist	0.67	IP3				95
**ACM2**	ICL3 replacement	C3-C(-1)	**ACM3**	C3-C(-1)			cAMP Inhibition		0.05		96

**ACM2**	Insertion	C1	A		agonist	0.08	cAMP Inhibition		0.62		97
**ACM2**	Insertion	C7	A		agonist	-0.08	cAMP Inhibition		0.88		97
**ACM3**	ICL3 replacement	N1-C3	**ACM2**	N1-C2	antagonist	-0.17	IP3		0.27		3
**ACM3**	ICL3 replacement	N1-N17	**ACM2**	N1-N16	antagonist	-1.74	IP3		0.17		3
**ACM3**	ICL3 replacement	N1-C3	**ACM2**	N1-C2	agonist	1.72	IP3		0.05		90
**ACM3**	ICL3 replacement	N1-N17	**ACM2**	N1-N16	agonist	0.27	IP3		0.01		90
**ACM3**	ICL3 replacement	N1-C3	**ACM2**		agonist	-0.33	current response		0.07		91
**ACM3**	ICL3 replacement	N1-N21	**ACM2**		agonist	-0.90	current response		0.16		91
**ACM3**	ICL3 replacement	N1-N12	**ACM2**		agonist	-0.42	current response		0.17		91
**ACM3**	ICL3 replacement	N13-N21	**ACM2**		agonist	-0.46	current response		0.67		91
**ACM3**	ICL3 replacement	N10-N21	**ACM2**		agonist	-0.62	current response		0.54		91
**ACM3**	ICL3 replacement	N5-N21	**ACM2**		agonist		current response		0.50		91
**ACM3**	Deletion	N3-N12			agonist	-0.19	current response		0.08		91
**ACM3**	Deletion	C17-C10			agonist		current response		0.97		91
**ACM3**	Deletion	C9-C6			agonist		current response		0.26		91
**ACM3**	Insertion	N9-C12	**T_4L**		agonist	0.77					98
**ACM3**	Insertion	N2		A	agonist	0.11	IP3	-0.99	1.08	3 and 5 alanine insertions disrupt signaling completely	99
**ACM3**	Directed Mutation	N3		F	agonist	0.15	IP3	-0.12	1.06	Point mutation to W or A at this site have different effects	93
**ACM3**	ICL3 replacement	N1-N16	**ACM2**	N1-N16	agonist	0.45	IP3		0.13	S(N3)Y rescues IP3 accumulation	93
**ACM3**	ICL3 replacement	N1-N16	**ACM2**	N1-N16	agonist	0.01	IP3		0.14		100
**ACM3**	ICL3 replacement	N1-N8	**ACM2**	N1-N8	agonist	0.60	IP3		0.19		100
**ACM3**	ICL3 replacement	N9-N19	**ACM2**	N9-N19	agonist	0.68	IP3	-0.51	0.96		100
**ACM3**	ICL3 replacement	N1-N4	**ACM2**	N1-N4	agonist	0.31	IP3	-0.82	0.27		100
**ACM3**	ICL3 replacement	N5-N8	**ACM2**	N5-N8	agonist	0.98	IP3	0.06	0.98		100
**ACM3**	Deletion	N1-N8			agonist	0.40	IP3		0.00		100
**ACM3**	Directed Mutation	N1	**ACM2**	N1	agonist	0.61	IP3	-0.45	1.22		100
**ACM3**	Directed Mutation	N2	**ACM2**	N2	agonist	0.45	IP3	-0.26	1.08		100
**ACM3**	Directed Mutation	N3	**ACM2**	N3	agonist	0.56	IP3	-0.82	0.33		100
**ACM3**	Directed Mutation	N4	**ACM2**	N4	agonist	0.22	IP3	-0.40	0.97		100
**ACM3**	Directed Mutation	N3-N7		FMLVL	agonist	0.00	current response		1.14		101
**ACM3**	Directed Mutation	N8-N12		MIVML	agonist	-0.22	current response		0.31		101
**ACM3**	Directed Mutation	N8,N10-N12		M,VML	agonist	-1.22	current response		1.24		101
**ACM3**	Deletion	N3-N7			agonist	0.00					101
**ACM3**	Directed Mutation	C8-C6,C4		VMN,T	agonist	-0.64	current response		0.22		101
**ACM3**	Deletion	N13-C13			agonist	-0.73	current response		0.77		101

**ACM3**	Deletion	N13-C10			agonist	-0.22			0.38		101
**ACM3**	Deletion	N13-C6			agonist	-0.30	current response		0.20		101
**ACM3**	Deletion, Directed Mutation	N13-C13,C8-C6,C4		VMN,T	agonist	-0.48	current response		0.14		101
**ACM3**	Insertion	C1	A		agonist	-0.26	IP3		0.16		97
**ACM3**	Insertion	C7	A		agonist	0.04	IP3		0.81		97
**ACM4**	Insertion	N10-C13	**T_4L**		agonist	-0.06					83
**ACM5**	Directed Mutation	N7	V					0.70	0.85		102
**ACM5**	Directed Mutation	N8, N10, N12	Q, S, N					0.40	1.13		102
**ACM5**	Directed Mutation	N5, N15	V, E					0.22	1.14		102
**ACM5**	Directed Mutation	N11	O					0.00	0.71		102
**ACM5**	Directed Mutation	N4	L					-0.04	1.35		102
**ACM5**	Directed Mutation	N5, N8, N12	L, O, A					-0.05	0.74		102
**ACM5**	Directed Mutation	N2, N10	L,S					-0.11	0.85		102
**ACM5**	Directed Mutation	N10, N15	I, E					-0.11	1.00		102
**ACM5**	Directed Mutation	N4, N6, N11, N14	W, A, M, G					-0.40	0.92		102
**ACM5**	Directed Mutation	N4, N17	W, L					-0.48	1.20		102
**ACM5**	Directed Mutation	N8, N13, N14	T, P, P					-0.76	0.79		102
**ACM5**	Directed Mutation	N1,N4,N5	O,O,A,V					-0.81	0.82		102
**ACM5**	Directed Mutation	N8,N15	D,R					-0.83	0.77		102
**ACM5**	Directed Mutation	N5	N,B					-0.89	0.72		102
**ACM5**	Directed Mutation	C10,C8,C7	G,K,S		agonist	0.04	IP3	0.40	1.10		103
**ACM5**	Directed Mutation	C21,C18,C13, C11-C7	R,P,PNRGS		agonist	0.09	IP3	0.40	1.20		103
**ACM5**	Directed Mutation	C15,C12	T,K				IP3	0.05	1.20		103
**ACM5**	Directed Mutation	C21,C9,C8	L,ET				IP3	-0.08	1.10		103
**ACM5**	Directed Mutation	C14,C3	I,K				IP3	-0.11	0.90		103
**ACM5**	Directed Mutation	C17,C16,C10	TQ,F				IP3	-0.15	1.20		103
**ACM5**	Directed Mutation	C17,C13-C11,C9	Q,GAO,R				IP3	-0.15	0.90		103
**ACM5**	Directed Mutation	C21,C19,C15, C12	R,I,N,I				IP3	-0.28	0.70		103
**ACM5**	Directed Mutation	C10,C9,C7	LT,T				IP3	-0.26	0.80		103
**ACM5**	Directed Mutation	C18,C17,C3	NG,H				IP3	-0.38	1.00		103
**ACM5**	Directed Mutation	C13,C12,C9,C6,C3,C2	GR,E,R,AM				IP3	-0.41	0.65		103
**ACM5**	Directed Mutation	C17,C9	G,E				IP3	-0.48	0.80		103
**ACM5**	Directed Mutation	C21,C20,C15, C13,C7	LP,R,E,S				IP3	-0.54	1.10		103
**ACM5**	Directed Mutation	C19,C12,C9	I,I,N				IP3	-0.62	1.00		103
**ACM5**	Directed Mutation	C17,C13,C9	G,S				IP3	-0.70	0.80		103
**ACM5**	Directed Mutation	C18,C17,C16, C14	R,QT,E				IP3	-0.95	0.80		103
**ACM5**	Directed Mutation	C20,C17,C16, C12,C5	P,NP,F,T				IP3	-0.95	1.00		103
**ACM5**	Directed Mutation	C19,C17,C10, C8,C6,C1	I,Q,R,V,H				IP3	-0.95	0.45		103
**ACM5**	Directed Mutation	C15,C12,C10	N,D,L				IP3	-1.11	0.80		103

**ACM5**	Directed Mutation	C20,C19,C12, C11,C9	PK,DO,				IP3	-1.20	1.20		103
**ACM5**	Directed Mutation	C18,C7,C3	I,T,P		agonist	0.11	IP3	-1.20	0.75		103
**ACM5**	Directed Mutation	C19,C18,C10, C5	IS,L,T				IP3	-1.20	0.90		103
**ACM5**	Directed Mutation	C21,C14,C11, C9,C8,C4,C2	Q,S,R,ND,T, K				IP3	-1.30	0.95		103
**ACM5**	Directed Mutation	C20,C2	H,P		agonist	0.16	IP3	-1.63	0.35		103
**ACM5**	Directed Mutation	C20,C19,C15-C13,C5,C3,C1	HI,MKL,Q,T, L,P				IP3	-1.65	0.5		103
**ACM5**	Directed Mutation	C10,C8-C6	L,DMT		agonist	-0.07	IP3	-1.81	0.55		103
**ACM5**	Directed Mutation	C21,C18,C12, C4,C2	Y,N,A,P,R				IP3	-1.88	0.5		103
**ACM5**	Directed Mutation	C13,C9,C4,C3	M,I,SP				IP3	-1.89	0.25		103
**ACM5**	Directed Mutation	C19,C14,C12, C7,C4	T,G,D,I,R				IP3	-1.94	0.7		103
**ACM5**	Directed Mutation	C19,C14,C13, C8	I,TE,D,N				IP3	-1.97	0.35		103
**ACM5**	Directed Mutation	C17,C15,C12, C10,C8,C4	R,M,F,G,D,T				IP3	-2.00	0.5		103
**ADA1B**	Deletion	N14-C12			agonist	0.60	IP1		0.80	$G\alpha_{16}$ Data	104
**ADA1B**	Deletion	N3-N5			agonist	0.01					104
**ADA1B**	Deletion	N6-C19			agonist	0.57					104
**ADA1B**	Deletion	N25-N32			agonist	0.02	IP1		0.20		104
**ADA1B**	Deletion	N25-C12			agonist	0.06					104
**ADA1B**	Deletion	N25-C5			agonist	0.37					104
**ADA1B**	Deletion	N3-N32			agonist	0.81					104
**ADA1B**	Directed Mutation	C6	L		agonist	0.09					104
**ADA1B**	Deletion,Directed Mutation	N25-N32,C6	L	C6	agonist	0.05					104
**ADA1B**	ICL3 replacement	N3-N5	**ADRB2**	N3-N5	agonist	0.99	IP3	0.08	0.55		105
**ADA1B**	ICL3 replacement	N18-N26	**ADRB2**	N10-N18	agonist	0.26	IP3	-0.33	0.55		105
**ADA1B**	ICL3 replacement	N28-N35	**ADRB2**	N17-N24	agonist	0.10	IP3		0.09		105
**ADA1B**	ICL3 replacement	C35-C30	**ADRB2**	N27-C24	agonist	0.16	IP3	0.37	0.67		105
**ADA1B**	ICL3 replacement	C9-C3	**ADRB2**	C9-C3	agonist	2.29	IP3	2.54	1.06		105
**ADA1B**	Directed Mutation	C9	**ADRB2**	C9	agonist	0.21	IP3	0.10	1.00		105
**ADA1B**	Directed Mutation	C7	**ADRB2**	C7	agonist	1.25	IP3	1.70	0.94		105
**ADA1B**	Directed Mutation	C4	**ADRB2**	C3	agonist	1.89	IP3	2.24	0.86		105
**ADA1B**	Directed Mutation	C4	T		agonist	1.11	IP3	1.21	0.95		106
**ADA2A**	ICL3 replacement	N(-19)-C(-6)	**ADRB2**	N(-2)-C(-19)	agonist	-1.66					1
**ADA2A**	ICL3 replacement	N1-N18	**ADRB2**	N1-N18	agonist	-0.07	cAMP Inhibition	-0.43	1.26		107
**ADA2A**	ICL3 replacement	N1-N18	**5HT1A**	N1-N18	agonist	0.14	cAMP Inhibition	-0.08	1.08		107
**ADA2A**	ICL3 replacement	N1-N18, C22-C6	**ADRB2**	N1-N18, C22-C6	agonist	-0.13	cAMP Inhibition	-1.81	0.87		107
**ADA2A**	ICL3 replacement	N1-N18, C22-C6	**5HT1A**	N1-N18, C22-C6	agonist	-0.16	cAMP Inhibition	-0.18	1.26		107
**ADA2A**	ICL3 replacement	C22-C6	**ADRB2**	C22-C6	agonist	0.56	cAMP Inhibition	0.18	0.79		107
**ADA2A**	ICL3 replacement	C22-C6	**5HT1A**	C22-C6	agonist	-0.04	cAMP Inhibition	-0.18	1.06		107
**ADA2A (pig)**	Directed Mutation	C16,C14,C12	A,A,A		agonist	0.50	cAMP inhibition	-0.12	1.02		108
**ADA2A (pig)**	Directed Mutation	C7,C6	A,A		agonist	0.50	cAMP inhibition	-1.30	0.96		108

**ADA2A (pig)**	Directed Mutation	C9,C7,C6	A,A,A		agonist	0.50	cAMP inhibition	-1.69	0.84		108
**ADA2A (pig)**	Directed Mutation	C9,C7,C6	A,A,A		antagonist	0.08	cAMP inhibition	-2.27	0.75		108
**ADRB2**	ICL3 replacement	N4-C4	**ADA1B**	N4-C4	agonist	0.63	cAMP	-0.48	0.28		109
**ADRB2**	ICL3 replacement	N4-N20,C9-C4	**ADA1B**	N4-N20,C9-C4	agonist	1.53	cAMP	-0.26	0.21		105
**ADRB2**	ICL3 replacement	N4-N13,C9-C4	**ADA1B**	N4-N13,C9-C4	agonist	1.51	cAMP	-0.22	0.26		105
**ADRB2**	ICL3 replacement	N9-N24	**ADA1B**	N9-N24	agonist	0.63	cAMP	-0.91	0.83		105
**ADRB2**	ICL3 replacement	N(-1)-N7	**ACM1**	N(-1)-N10	agonist	0.70	cAMP		0.01		110
**ADRB2**	Directed Mutation	C17	G		agonist	0.66	cAMP	0.65	0.96		2
**ADRB2**	Directed Mutation	C18-C21	KRFI		agonist	-0.19	cAMP	-0.54	0.78		2
**ADRB2**	Deletion	C9-C3			agonist	0.49	cAMP	-0.46	0.54		2
**ADRB2**	Deletion	C13-C3			agonist	0.06	cAMP	-0.23	0.83		2
**ADRB2**	Deletion, Directed Mutation	C9-C(-4)	VLAVVI								2
**ADRB2**	Deletion	N1-N7	E,F								2
**ADRB2**	Deletion	N2-N8									2
**ADRB2**	Directed Mutation	N(-5)-N5	ILVYVRIYQI	N(-5)-N5	agonist	0.92	cAMP	0.50	0.77		2
**ADRB2**	Deletion	C9-C3			agonist	-0.05	cAMP		0.46	Derived from Table 1 (Iso/FSK) and Table 2 (cyclase mix)	111
**ADRB2**	Insertion	N10-C13	**T$_4$L**		agonist	0.51					112
**ADRB2**	Deletion	N16-C13			agonist	-0.19					113
**ADRB2**	Insertion	N13-C9	**T$_4$L**		agonist	0.60					24
**ADRB2**	Directed Mutation	C10,C9,C7,C4	S,R,K,A		agonist	1.34	cAMP	0.89	1.00	+GppNhp condition	114
**ADRB2 (hamster)**	Deletion	N9-N16			agonist	-0.54	cAMP	0.60	1.11		115
**ADRB2 (hamster)**	Deletion	N19-C4			agonist	0.58	cAMP				115
**ADRB2 (hamster)**	Deletion	N9-N16			agonist	-0.41	cAMP	-0.18	0.67		115
**ADRB2 (hamster)**	Deletion	N19-C4			agonist	-0.40	cAMP	-0.40	0.67		115
**ADRB2 (hamster)**	Deletion	C4-C(-1)			agonist	-0.40	cAMP	-0.30	0.89		115
**ADRB2 (hamster)**	Directed Mutation	N7, N8, N12, N15	S,S,S,S		agonist	-0.60	cAMP	-0.37	0.77		116
**ADRB2 (hamster)**	Directed Mutation	N7, N12, N15	S,S,S		agonist	-0.85	cAMP	-0.52	0.87		116
**ADRB2 (hamster)**	Directed Mutation	N2, N3, N5, N6, N13	L,L,L,L,L		agonist	0.40	cAMP	-0.48	0.30		116
**ADRB2 (hamster)**	Directed Mutation	N1, N2, N3, N4, N5, N6, N7, N8, N9	K,A,L,A,A,L, A,K,K		agonist	-0.78	cAMP	-0.43	0.20		116
**ADRB2 (hamster)**	Deletion	N(-1)-N7			agonist	1.46	cAMP		0.31	+GppNhp condition	7
**ADRB2 (hamster)**	Deletion	N7-N14			agonist	-0.18	cAMP	0.53	0.97	+GppNhp condition	7
**ADRB2 (hamster)**	Deletion	N16-N29			agonist	1.82	cAMP	0.28	1.97	+GppNhp condition	7
**ADRB2 (hamster)**	Deletion	N21-C6			agonist	1.82	cAMP		0.31	+GppNhp condition	7
**ADRB2 (hamster)**	Deletion	N28-C19			agonist	1.00	cAMP	-0.25	0.94	+GppNhp condition	7
**ADRB2 (hamster)**	Deletion	C20-C8			agonist	-0.30	cAMP		0.41	+GppNhp condition	7
**ADRB2 (hamster)**	Deletion	N8-C12			agonist	-0.40	cAMP	-1.60	0.50	+GppNhp condition	117
**AGTR1**	Directed Mutation	N4,N7,N8,N10	Q,Q,Q,F		agonist	-0.37	IP3		1.13		118

**AGTR1**	Directed Mutation	C13,C11,C9,C7,C6	Q,Q,Q,G,G		agonist	0.00	IP3		0.77		118
**AGTR1**	ICL3 replacement	N2-C7	**AGTR2**	N2-C7	agonist	-0.06	gene expression		0.05		119
**AGTR1**	ICL3 replacement	N10-C7	**AGTR2**	N10-C7	agonist	0.12	gene expression		0.47		119
**AGTR1**	ICL3 replacement	N3-N9	**AGTR2**	N3-N9	agonist	0.16	gene expression		0.30		119
**AGTR1**	ICL3 replacement	N3-N10	**AGTR2**	N3-N10	agonist	-0.07	gene expression		0.06		119
**AGTR1**	ICL3 replacement	N3-C10	**AGTR2**	N3-C10	agonist	0.11	gene expression		0.24		119
**AGTR1**	ICL3 replacement	C13-C7	**AGTR2**	C13-C7	agonist	0.09	gene expression		0.54		119
**AGTR1**	ICL3 replacement	N10-C10	**AGTR2**	N10-C10	agonist	0.02	gene expression		0.92		119
**C5AR1**	Directed Mutation	N7					gene expression		0.55		120
**C5AR1**	Directed Mutation	N12					gene expression		0.40		120
**C5AR1**	Directed Mutation	C9					gene expression		0.25		120
**C5AR1**	Directed Mutation	C8					gene expression		0.20		120
**C5AR1**	Directed Mutation	C6					gene expression		0.10		120
**CCR2**	ICL3 replacement	N(-1)-C1	**CXCR1**	N(-1)-C1			IP3		0.08		121
**CXCR4**	Insertion	N9-N10	**T$_4$L**		antagonist	-0.31					122
**CXCR4**	Insertion	N9-N10	GS-**T$_4$L**-GS		antagonist	-0.33					122
**DRD2**	Directed Mutation	N23	G		agonist	0.04	cAMP inhibition	-0.12	0.94		123
**DRD2**	Directed Mutation	N24	T		agonist	-0.06	cAMP inhibition	-0.12	1.09		123
**DRD2**	Deletion	N32-N60			antagonist	-0.13	cAMP inhibition	0.25	1.00		124
**DRD2**	Directed Mutation	N41	V		antagonist	0.11	cAMP inhibition	-0.13	1.02		124
**DRD2**	Directed Mutation	N48,N57-N59	V,V,V,V		antagonist	0.13	cAMP inhibition	-0.45	1.03		124
**DRD2**	Directed Mutation	N48,N57-N59,N64,N65	V,V,V,V,V,V		antagonist	0.19	cAMP inhibition	-0.40	0.81		124
**DRD2**	Directed Mutation	N54	G		antagonist	-0.05	cAMP inhibition	-0.21	1.02		124
**DRD2**	Directed Mutation	N49,N52	A,A		antagonist	0.03	cAMP inhibition	0.43	0.96		124
**DRD2**	Directed Mutation	N39	V		antagonist	-0.10	cAMP inhibition	0.72	0.93		124
**DRD2**	Directed Mutation	N61	V		antagonist	0.04	cAMP inhibition	-0.02	0.96		124
**DRD2**	Deletion	N32-N60			agonist	0.05	cAMP inhibition		1.17		125
**DRD3**	Insertion	N13-C14	**T$_4$L**		agonist	-0.47					126
**FFAR1**	Insertion	N9-N10	**T$_4$L**		agonist	-0.03				additional mutations	127
**FFAR1**	Insertion	N9-N10	GS-**T$_4$L**-GS		agonist	0.10				additional mutations	127
**GASR (rat)**	Directed Mutation	C8	M		agonist	0.09	IP3		0.71		128
**GASR (rat)**	Directed Mutation	C7	T		agonist	-0.04	IP3		0.24		128
**GASR (rat)**	Directed Mutation	C7	L		agonist	-0.10	IP3		0.07		128
**GLP1R**	Deletion	N12-N14			agonist	0.08	cAMP		0.80	Also performed point mutations in this region	129
**GLP1R**	Deletion	N9-N11			agonist	0.07	cAMP		0.10		129
**GLP1R**	Deletion	N6-N8			agonist	0.33	cAMP		0.05		129
**GLP1R**	Deletion	N4-N5			agonist	0.22	cAMP		0.20		129
**GLP1R**	Deletion	N2-N3			agonist	0.13	cAMP		0.05		129
**HRH1**	Insertion	N11-C14	**T$_4$L**		agonist	0.07					130

**NMBR**	ICL3 replacement	N3-C4	**ACM3**	N1-C2	agonist	0.08	IP3		0.08		131
**NTS1**	Insertion	N11-C12	**T_4L**		agonist	-0.06				additional mutations	132
**NTS1**	Deletion	N19-C16	**T_4L**		agonist	0.09	GTPγS turnover		0.40	additional mutations	133
**NTS1**	Insertion	N11-C6	**T_4L**		agonist	-0.06				additional mutations	134
**OPN2**	ICL3 replacement	N8-C12	**ADRB2**	N8-C12			G_t activation		0.08		135
**OPN2**	ICL3 replacement	N9-C12	**ADRB2**	N8-C12			G_t activation		0.1		
**OPRK**	Insertion	C16-C14	**T_4L**		agonist	0.14				I135L mutation	136
**OPRM**	Insertion	N11-C15	**T_4L**		antagonist	0.04					137
**OPSD (bovine)**	Deletion	N12-C6					GtPase Activity		0.00		138
**OPSD_bovine**	ICL3 replacement	N6-C3	**PTGER3 (mouse)**	N7-C3			G_t activation		0.1		139
**OXTR**	ICL3 replacement	N4-C3	**V2R**	N4-C3	agonist	-1.03					140
**OXTR**	Directed Mutation	C10	A		agonist	-0.18	IP3	-0.08	2622.00		140
**OXTR**	Directed Mutation	C8	V		agonist	-0.69					140
**OXTR**	Directed Mutation	C10,C8	A,V		agonist	-0.80					140
**P2Y12**	Insertion	N13	**BRIL**		agonist	-0.09					141
**PACR**	Insertion	N7	TNLRLRVPK KTREDPLPV PSDQHSPP FL		agonist	-0.30	IP3	-0.10			142
**PACR**	Insertion	N7	SCVQKCYC KPQRAQQH SCKMSELS TITL		agonist	0.00	IP3	-0.10			142
**PTH1R**	Directed Mutation	N4-N7	AAAA		agonist	2.86	cAMP	-0.66	0.63		143
**PTH1R**	Directed Mutation	N8-N11	AAAA		agonist	3.23	cAMP	-0.64	0.40		143
**PTH1R**	Directed Mutation	N12-C17	AAAA		agonist	3.01	cAMP	-0.08	0.85		143
**PTH1R**	Directed Mutation	C16-C13	AAAA		agonist	3.02	cAMP	0.33	1.02		143
**STE3**	Deletion	N12-N14					gene expression		0.56		144
**STE3**	Deletion	N15-C20					gene expression		2.08		144
**STE3**	Deletion	N19-C17					gene expression		2.22		144
**STE3**	Deletion	C20-C15					gene expression		1.94		144
**STE3**	Deletion	C16-C11					gene expression		1.94		144
**STE3**	Deletion	C12-C7					gene expression		0.56		144
**TSHR**	Directed Mutation	C10-C6,C3,C2	NQNAG,Q,G		agonist	-0.93	cAMP		0.84		145
**TSHR**	Directed Mutation	N1,N3,N5,N7, C14	G,A,A,G,A		agonist	0.25	cAMP	0.39	0.21		145
**TSHR (rat)**	ICL3 replacement	C4	K		agonist	0.92	cAMP	-0.04	0.73	E mutation at same position had similar effects	146
**TSHR**	ICL3 replacement	N5-N7	**ADA1A**	N5-N7	agonist	0.04	cAMP	-0.22	0.69		147
**TSHR**	ICL3 replacement	N3-N7	**ADRB2**	N3-N7	agonist	0.09	cAMP	-0.48	0.74		147
**TSHR**	ICL3 replacement	N8-N11	**ADA1A**	N8-N11	agonist	0.07	cAMP	0.04	0.97		147
**TSHR**	ICL3 replacement	C10-C7	**ADA1A**	C11-C8	agonist	0.85	cAMP	-0.67	0.86		147
**TSHR**	ICL3 replacement	C5-C2	**ADA1A**	C6-C3	agonist	-0.02	cAMP	-0.63	0.60		147
**TSHR**	ICL3 replacement	C6-C2	**ADRB2**	C7-C3	agonist	0.54	cAMP	-0.80	0.57		147

**TSHR**	ICL3 replacement	N8-N11	**ADRB2**	N8-N11	agonist	0.32	cAMP	-0.03	0.91		147
**TSHR**	ICL3 replacement	C10-C7	**ADRB2**	C11-C8	agonist	0.85	cAMP	-0.55	1.03		147
**V1AR (rat)**	ICL3 replacement	N(-1)-C(-1)	**V2R**	N(-1)-C(-1)	agonist	0.30	cAMP	-0.29	0.76		148
**V1AR (rat)**	ICL3 replacement	N(-1)-C22	**V2R**	N(-1)-C22	agonist	-0.16	cAMP	-0.33	0.71		148
**V1AR (rat)**	ICL3 replacement	N13-C(-1)	**V2R**	N13-C(-1)	agonist	0.32	cAMP		0.11		148
**V1AR (rat)**	ICL3 replacement	N(-1)-N10	**V2R**	N(-1)-N10	agonist	-0.27	cAMP	-0.86	0.29		148
**V1AR (rat)**	ICL3 replacement	N(-1),N1	**V2R**	N(-1),N1			cAMP		0.10		148
**V1AR (rat)**	ICL3 replacement	N4-N6	**V2R**	N4-N6			cAMP		0.05		148
**V1AR (rat)**	ICL3 replacement	N8-N10	**V2R**	N8-N10			cAMP		0.04		148
**V1AR (rat)**	ICL3 replacement	N(-1),N1,N13-C(-1)	**V2R**	N(-1),N1,N13-C(-1)			cAMP		0.20		148
**V1AR (rat)**	ICL3 replacement	N4-N6,N3-C(-1)	**V2R**	N4-N6,N3-C(-1)	agonist	-0.01	cAMP	-0.86	0.44		148
**V1AR (rat)**	ICL3 replacement	N8-N10,N13-C(-1)	**V2R**	N8-N10,N13-C(-1)			cAMP		0.09		148
**V2R**	ICL3 replacement	N4-C3	**OXTR**	N4-C3	agonist	-0.06					140
**V2R**	ICL3 replacement	N4-N16	**OXTR**	N4-N16	agonist	-0.75	cAMP		0.44		140
**V2R**	ICL3 replacement	N19-C3	**OXTR**	N19-C3	agonist	-0.21	cAMP	-0.30	0.85		140

Supplementary Table 1. Effect of ICL3 mutagenesis on receptor pharmacology. Column 1, Receptor gene name (Uniprot ID). Column 2, Type of mutation: Insertion refers to insertion of a protein domain, deletion refers to truncation of amino acids, ICL3 replacement refers to switching ICL3 domain from one receptor to another, directed mutation refers to mutagenesis of individual amino acid(s). Column 3, Location of mutation: Nx refers to sequence position relative to TM5.56, read N-C. Cx refers to sequence position relative to TM6.37, read C-N. Column 4, new sequence. Entries in bold reflect new domains or new ICL3 sequences. T_4L refers to T_4-lysozyme, BRIL refers to apocytochrome b_{562}RIL. Column 6, Positions of new ICL3 sequences (for ICL3 replacement mutations), using the same numbering scheme as column 3. Column 4, ligand class used for binding assays (agonist vs antagonist). Column 5, comparison of ligand binding for the wild-type receptor versus the mutant. Column 6, Assay: refers to the measurement technique for assessing signaling potency (EC_{50}) and/or efficacy (E_{max}). Measurements curated are for canonical signaling for the wild-type receptor. Column 7, comparison of signaling potency for the wild-type receptor versus the mutant. Column 8, comparison of signaling efficacy for the wild-type receptor versus the mutant. Column 9, notes on which values are curated and/or additional mutations noted for the receptor which may affect changes in receptor function. Column 10, reference from which data are derived.

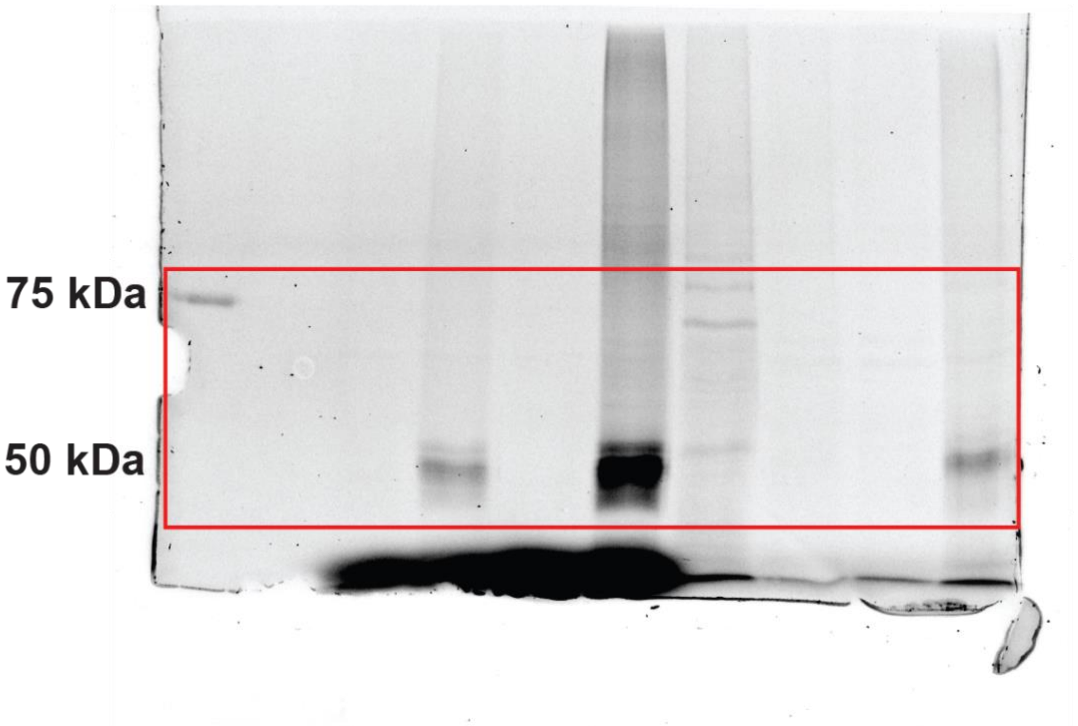

Supplementary Figure 1. Uncropped image from Extended Data Figure 3e. Boxes refer to the cropped areas in the figure, band in leftmost lane (75 kDa) is from the autofluorescence of ladder.

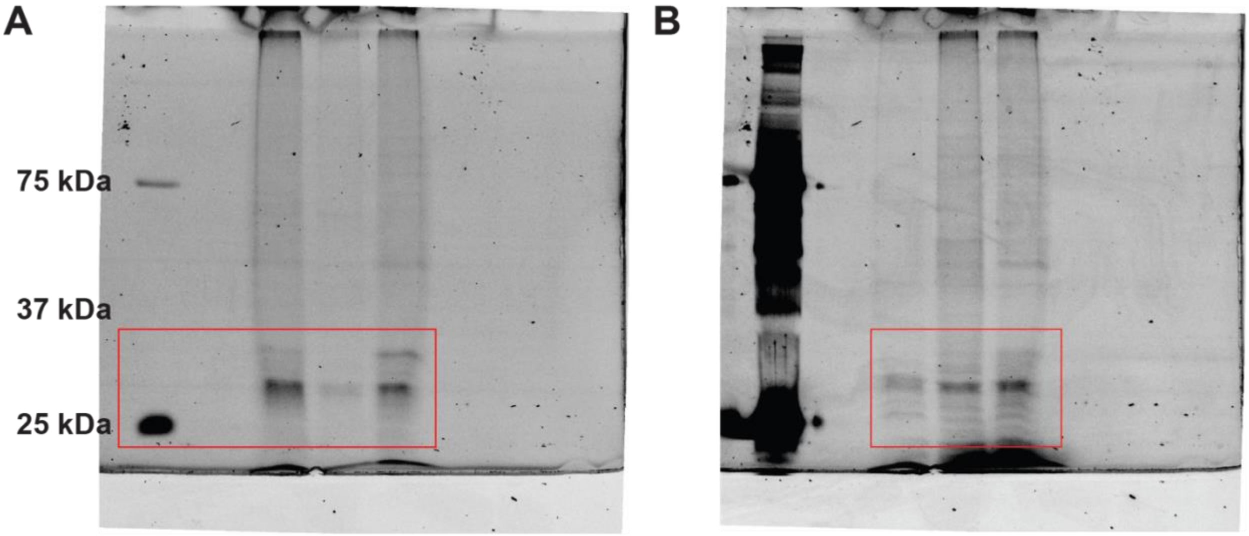

<u>Supplementary Figure 2.</u> Uncropped image from Extended Data Figure 4n. (**A**) is the 488 gel scan, and (**B**) is the 546 gel scan. The 10% polyacrylamide gel matrix prevents complete migration of labeled bands, evident in the signal at the interface of stacking and resolving gels (top of the lanes). Lower % acrylamide facilitates entry of fluorescent protein (note the lack of bands at the stacking-resolving interface in Supplementary Figure 1). However, it does not resolve the lower molecular weight bands. Boxes refer to the cropped areas in the figure. Bands at 75 kDa and 25 kDa in the leftmost lane is from the auto-fluorescent ladder (labeled).

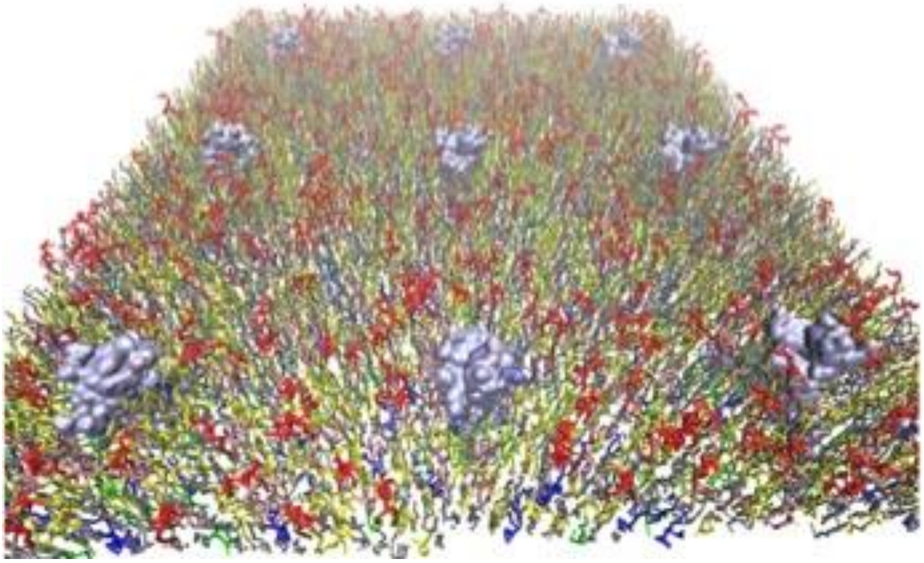

Supplementary Figure 3. Starting model of the cell membrane consisting of multiple lipids used in the coarse grain simulations to equilibrate the multi-lipid bilayer[22].

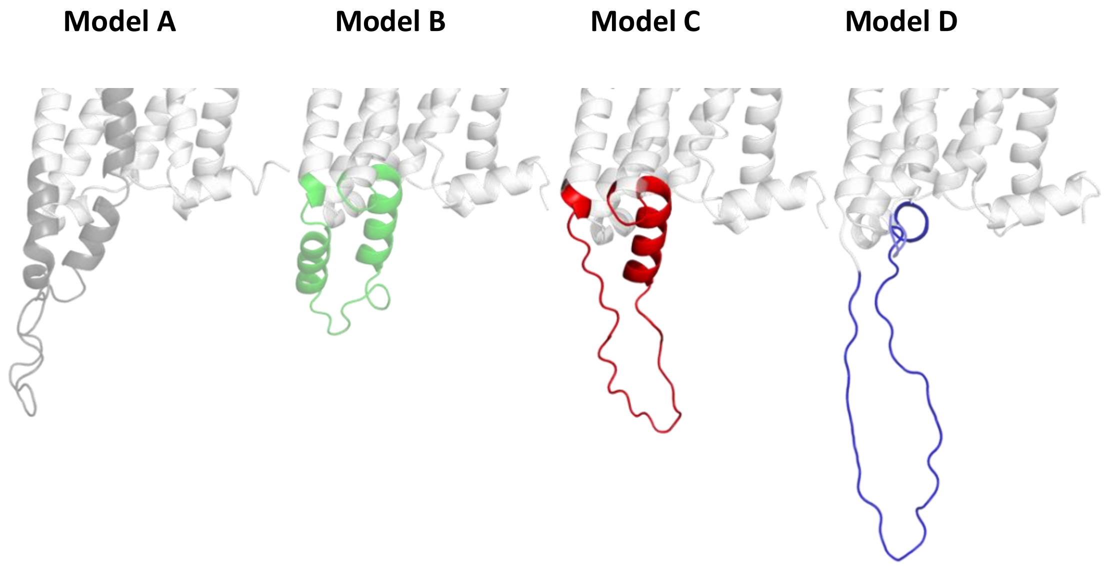

Supplementary Figure 4. Structural models of ICL3 in β_2AR used as starting structures for MD simulations in cell membrane conditions.

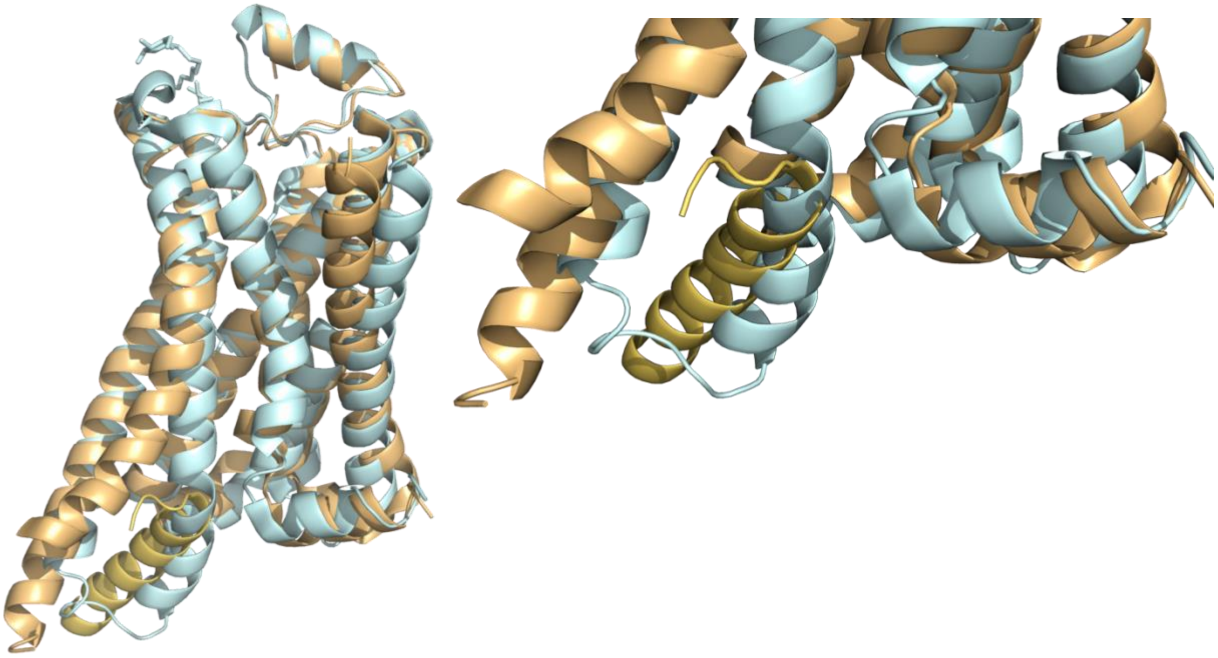

Supplementary Figure 5. β_1AR inactive state structure displays cytoplasmic TM6 conformation similar to the Gα_s C-terminus (G_s peptide). Left: Overlay of the inactive state structure of β_1AR (pale cyan) (PDB ID: 2ycx)[23] with the fully active state structure of β_2AR (orange) bound to the G_s protein (PDB ID: 3sn6)[17]. The last 24 amino acids of the Gs protein are shown (gold). Right: Close up view of the two structures showing that the intracellular part of helix 6 and ICL3 blocks the G protein binding site in the inactive state.

Starting Structure used for MD simulations	Number of simulations	Simulation time for each velocity (µs)	Total simulation time (µs)
Model A	5	0.4	2
Model B	5	0.4	2
Model C	5	0.4	2
Model D	25	0.4	10
*Starting structure: snapshot at end of 50 ns (Model D).	5	0.4	2
*Starting structure: snapshot at end of 100 ns (Model D).	5	0.4	2
*Starting structure: snapshot at end of 150 ns (Model D).	5	0.4	2

Supplementary Table 2. Simulation parameters (see Methods for details). A swarm of MD simulations, starting from each structural template of ICL3, was used to create the conformational landscape of ICL3 spanning. Bottom 3 rows reflect simulations starting from timepoints within the “Model D” simulation trajectories. These were used to better sample transitions between “intermediate” and “open” states of ICL3.

Receptor	Sequence	Ligand
**β_2AR WT**	FVYSRVFQEAKRQLQKIDKSEGRFHVQNLSQVEQDGRTGHGLRRSSKFCLKEHKALKTL	Isoproterenol (100 µM), Metoprolol (100 µM) for FSK Inhibition
**β_2AR ΔICL3**	FVYSRVFQEAKRQLQKIDK---------------------------KFCLKEHKALKTL	
**β_1AR WT**	FVYLRVFREAQKQVKKIDSCERRFLGGPARPPSPSPSPVPAPAPPPGPPRPAAAAATAPLANGR AGKRRPSRLVALREQKALKTL	Isoproterenol (100 µM), Metoprolol (100 µM) for FSK Inhibition
**β_1AR ΔICL3**	FVYLRVFREAQKQVKKID-- AGKRRPSRLVALREQKALKTL	
**D$_1$R WT**	VTYTRIYRIAQKQIRRIAALERAAVHAKNCQTTTGNGKPVECSQPESSFKMSFKRETKVLKTL	Dopamine (100 µM)
**D$_1$R ΔICL3**	VTYTRIYRIAQKQIRRIAA-------------------------PESSFKMSFKRETKVLKTL	
**M$_1$R WT**	TLYWRIYRETENRARELAALQGSETPGKGGGSSSSSERSQPGAEGSPETPPGRCCRCCRAPRLLQAYSWKEE EEEDEGSMESLTSSEGEEPGSEVVIKMPMVDPEAQAPTKQPPRSSPNTVKRPTKKGRDRAGKGQKPRGKEQL AKRKTFSLVKEKKAARTL	Carbachol (100 µM)
**M$_1$R ΔICL3**	TLYWRIYRETENRARELAA--- -- AKRKTFSLVKEKKAARTL	
**V$_{1A}$R WT**	TCYGFICYNIWCNVRGKTASRQSKGAEQAGVAFQKGFLLAPCVSSVKSISRAKIRTVKMT	Arginine vasopressin (100 nM)
**V$_{1A}$R ΔICL3**	TCYGFICYNIWCNVRGKTA-----------------------VSSVKSISRAKIRTVKMT	
**A$_{1A}$R WT**	LIYLEVFYLIRKQLNKKVSASSGDPQKYYGKELKIAKSL	N$_6$-methyladenosine (10 µM)
**A$_{1A}$R ΔICL3**	LIYLEVFYLIRKQLNKKV-------QKYYGKELKIAKSL	
**CB$_1$R WT**	YAYMYILWKAHSHAVRMIQRGTQKSIIIHTSEDGKVQVTRPDQARMDIRLAKTL	2-Arachidonoylglycerol (10 µM)
**CB$_1$R ΔICL3**	YAYMYILWKAHSHAVRMIQ-----------------QVTRPDQARMDIRLAKTL	

Supplementary Table 3. Receptor ICL3 truncations. Column 2 is the ICL3 sequence (TM5.56-TM6.37). Column 3 is the saturating agonist concentration used for signaling assays.

Insertion	Key		Inserted Sequence	Gs signal	N	Gq signal	N
**Wild-Type**				0.56±0.13	15	0.18±0.08	12
**CB$_2$R**	G_i	1	GHQDRQ	0.55±0.06	3	0.16±0.02	3
**CB$_1$R**	G_i	2	RGTQKSIIIHTSEDGKV	0.53±0.12	3	0.10±0.07	3
**D$_3$R**	G_i	3	TRQNSQCNSVRPGFPQQTLSPDPAHLELKRYYSICQDTALGGPGFQERGGELKREEKTRNSLSPTIAPKLSLEVRKLSNGRLSTSLKLG	0.72±0.11	5	0.08±0.17	3
**5HT1D**	G_i	4	LYGKRFTTAHLITGSAGSSLCSLNSSLHEGHSHSAGSPLFFNHVKIKLADSA	0.24±0.05	3	-0.15±0.12	3
**M$_2$R**	G_i	5	EPVANQDPVSPSLVQGRIVKPNNNNMPSSDDGLEHNKIQNGKAPRDPVTENCVQGEEKESSNDSTSVSAVASNMRDDEITQDENTVSTSLGHSKDENSKQTCIRIGTKTPKSDSCTPTNTTVEVVGSSGQNGDEKQNIVARKIVKMTKQ	0.35±0.09	3	0.10±0.10	5
**β$_1$AR**	G_s	1	CERRFLGGPARPPSPSPSPVPAPAPPPGPPRPAAAAATAPLANGRAGK	0.47±0.07	4	0.06±0.03	3
**D$_1$R**	G_s	2	LERAAVHAKNCQTTTGNGKPVECSQP	0.58±0.13	5	0.13±0.14	4
**β$_2$AR**	G_s	3	SEGRFHVQNLSQVEQDGRTGHG	0.56±0.17	4	0.1±0.09	4
**H$_1$R**	G_q	1	INRSLPSFSEIKLRPENPKGDAKKPGKESPWEVLKRKPKDAGGGSVLKSPSQTPKEMKSPVVFSQEDDREVDKLYCFPLDIVHMQAAAEGSSRDYVAVNRSHGQLKTDEQGLNTHGASEISEDQMLGDSQSFSRTDSDTTTETAPGKGKLRSGSNTGLDYIKFTWKRLRSHSR	0.66±0.13	3	-0.09±0.08	3
**5HT2A**	G_q	2	DLGTRAKLASFSFLPQSSLSSEKLFQRSIHREPGSYT	0.37±0.07	3	0.02±0.05	3
**α$_{1A}$AR**	G_q	3	GLKTDKSDSEQVTLRIHRKNAPAGGSGMASAKTKTH	0.59±0.19	4	0.14±0.06	4
**GPR27**	G_q	4	MRPARLVPAVSHDWTFHGPGATGQAAANWTAGFGRGPTPPALVGIRPAGPGRGAR	0.79±0.1	3	0.14±0.06	3
**M$_1$R**	G_q	5	LQGSETPGKGGGSSSSSERSQPGAEGSPETPPGRCCRCCRAPRLLQAYSWKEEEEEDEGSMESLTSSEGEEPGSEVVIKMPMVDPEAQAPTKQPPRSSPNTVKRPTKKGRDRAGKGQKPRGKEQL	0.44±0.13	5	0.26±0.07	3
**CCK$_2$R**	G_q	6	SDSDSQSRVRNQGGLPGAVHQNGRCRPETGAVGEDSDGCYVQLPRSRPALELTALTAPGPGSG	0.42±0.06	4	0.12±0.14	4
**V$_{1A}$R**	G_q	7	SRQSKGAEQAGVAFQKGFLLAPC	0.33±0.04	3	0.01±0.13	3
**5HT2C**	G_q	8	GHTEEPPGLSLDFLKCCKRNTAEEENSANPNQDQNARRRKKKERR	0.48±0.08	3	0.00±0.08	3
**NTS$_1$R**	G_q	9	QGQVCTVGGEHSTFS	0.48±0.14	6	0.14±0.18	6
**NTS$_2$R**	G_q	10	TSTPGSSTPSRLELLSEEGLLSFIVWKKTFIQGGQVSLV	0.36±0	4	-0.10±0.12	3

Supplementary Table 4. Sequence information and data from PTH1R-ICL3 insertion screen (Figure 5). Column 1, names of host receptors from which inserted ICL3s are derived. Wild-Type indicates Wild-Type PTH$_1$R. Columns 2-3, key for Figure 5F and G. Column 2 indicates primary G protein subtype of host receptor. Column 4, inserted ICL3 sequence. Column 5, specific G_S-peptide luminescence signal (mean ± standard deviation). Column 6, number of biological replicates for G_S-peptide assay. Column 7, specific G_q-peptide luminescence signal (mean ± standard deviation). Column 8, number of biological replicates.

Receptor	Positions	Insertion	Positions	Assay	pEC50 (Wild-Type)	pEC50 (Mutant)	Emax (Wild-Type)	Emax (Mutant)	Notes	Reference
**ACM1**	N25-C11	**ADRB1 (turkey)**	N22-C11	cAMP			1	49	derived from table 2 - PTX data	[81]
**ACM2**	N(-7)-C1	**ACM1**	N(-7)-C2	current response			210	2910		[82]
**ACM2**	N1-C2	**ACM3**	N1-C3	IP3	7.8	9.3	41	233		[3]
**ACM2**	N1-N16	**ACM3**	N1-N17	IP3	7.8	8.3	41	137		[3]
**ACM2**	N1-C2	**ACM3**	N1-C3	IP3	8.6	7.8	61	73		[90]
**ACM2**	N1-N16	**ACM3**	N1-N17	IP3	8.6	7.7	61	26		[90]
**ACM2**	N1-N25	**ADRB1 (Turkey)**	N1-N26	cAMP			0	400		[94]
**ACM2**	N1-N21	**ACM3**	N1-N21	IP3		5.54	11	40		[95]
**ACM3**	N1-C3	**ACM2**	N1-C2	cAMP Inhibition	-	9.9	1	18		[90]
**ACM3**	N1-N17	**ACM2**	N1-N16	cAMP Inhibition	-	10.3	1	11		[90]
**ACM3**	N11-C4	**GPR183**	N12-C9	G_{12} dissociation			0	0.34	Mutations-Y149C and A239G	[37]
**ACM3**	N11-C5	**NMBR**	N12-C9	G_{12} dissociation			0	0.08	Mutations-Y149C and A239G	[37]
**ACM3**	N11-C6	**S1PR2**	N12-C9	G_{12} dissociation			0	0.07	Mutations-Y149C and A239G	[37]
**ACM3**	N11-C7	**GPR132**	N12-C9	G_{12} dissociation			0	0.29	Mutations-Y149C and A239G	[37]
**ACM3**	N11-C8	**LTB4R2**	N12-C9	G_{12} dissociation			0	0.17	Mutations-Y149C and A239G	[37]
**ACM3**	C3-C(-1)	**ACM2**	C3-C(-1)	cAMP Inhibition			1	30		[96]
**ACM3**	N1-N16, C3-C(-1)	**ACM2**	C3-C(-1)	cAMP Inhibition			1	35		[96]
**ADA2A**	N(-19)-C(-6)	**ADRB2**	N(-2)-C(-19)	cAMP	-	6.3	0	35		[1]
**ADRB2**	N4-C4	**ADA1B (Hamster)**	N4-C4	IP3	-	9.4	0	162		[149]
**ADRB2**	N4-N20,C9-C4	**ADA1B (Hamster)**	N4-N20,C9-C4	IP3	-	11.0	0	155		[149]
**ADRB2**	N4-N20	**ADA1B (Hamster)**	N4-N20	IP3	-	10.5	0	135		[149]
**ADRB2**	N4-N13,C9-C4	**ADA1B (Hamster)**	N4-N13,C9-C4	IP3			0	10		[149]
**ADRB2**	N19-N24	**ADA1B (Hamster)**	N19-N24	IP3			0	10		[149]
**ADRB2**	N14-N24	**ADA1B (Hamster)**	N14-N24	IP3			0	9		[149]
**ADRB2**	N9-N24	**ADA1B (Hamster)**	N12-N24	IP3			0	12		[149]
**ADRB2**	N9-N24	**ADA1B (Hamster)**	N9-N24	IP3	-	9.4	0	110		[149]
**ADRB2**	N9-N18	**ADA1B (Hamster)**	N9-N18	IP3			0	18		[149]
**AGTR2**	N2-C7	**AGTR1**	N2-C7	gene expression			20	60		[119]
**CXCR1**	N(-1)-C1	**CCL2**	N(-1)-C1	IP3			100	300		[121]
**OPN2**	N1-C4	**ADRB2**	N1-C4	cAMP			0	0.61		[135]
**OPSD (bovine)**	N6-C3	**mAChR1 (pig)**	N7-C3	Gq activation			-	5		[139]
**OPSD (bovine)**	N6-C3	**mAChR2 (pig)**	N7-C3	Gi activation			-	140		[139]
**OPSD (bovine)**	N6-C3	**ADA1B (hamster)**	N7-C3	Gq activation			-	5		[139]
**OPSD (bovine)**	N6-C3	**ADA2B (human)**	N7-C3	Gi activation			-	40		[139]
**OPSD (bovine)**	N6-C3	**ADRB2 (human)**	N7-C3	Gs activation			-	40		[139]
**OPSD (bovine)**	N6-C3	**PTGER2 (mouse)**	N7-C3	Gs activation			-	1		[139]
**OPSD (bovine)**	N6-C3	**PTGER3 (mouse)**	N7-C3	Gi activation			-	50		[139]

**OPSD (bovine)**	N6-C3	**EDNRA (human)**	N7-C3	G protein activation			-	140		[139]
**OPSD (bovine)**	N6-C3	**EDNRB (human)**	N7-C3	G protein activation			-	125		[139]
**OPSD (bovine)**	N6-C3	**ADRB2 (human)**	N7-C3	Gs activation			-	40		[139]
**PACR (rat)**	N24-C23	**PAFR (rat)**	N(-2)-C1	IP3			1000	5000		[150]
**V1AR (rat)**	N13-C(-1)	**V2R**	N13-C(-1)	IP3			-	70		[148]
**V1AR (rat)**	C16-C(-1)	**V2R**	N(-1)-N10	IP3			-	72		[148]
**V1AR (rat)**	N8-N10	**V2R**	N8-N10	IP3			-	75		[148]
**V1AR (rat)**	N(-1),N1,N13-C(-1)	**V2R**	N(-1),N1,N13-C(-1)	IP3			-	100		[148]
**V1AR (rat)**	N8-N10,N13-C(-1)	**V2R**	N8-N10,N13-C(-1)	IP3			-	100		[148]
**V2R**	N4-C3	**OXTR**	N4-C3	IP3	8.49	9.21	1019	3714		[140]
**V2R**	N19-C3	**OXTR**	N19-C3	IP3	8.49	8.19	1019	2668		[140]

Supplementary Table 5. **Gain-of-function for new signaling pathways by ICL3 mutagenesis**. Column 1, Receptor gene name (Uniprot ID). Column 2, Location of mutation: Nx refers to sequence position relative to TM5.56, read N-C. Cx refers to sequence position relative to TM6.37, read C-N. Column 3, new sequence. Column 4, Positions of new ICL3 sequences (for ICL3 replacement mutations), using the same numbering scheme as column 3. Column 5, Assay: refers to the measurement technique for assessing signaling potency (EC_{50}) and/or efficacy (E_{max}). Measurements curated are for pathways that the wild-type receptor doesn't natively signal through, or weakly signals through. Columns 6-7, comparison of signaling potency for the wild-type receptor versus the mutant. Columns 8-9, comparison of signaling efficacy for the wild-type receptor versus the mutant. Column 10, notes on which values are curated and/or additional mutations noted for the receptor which may affect changes in receptor function. Column 11, reference from which data is derived.

References

73. Wang, C. *et al.* Structural basis for molecular recognition at serotonin receptors. *Science.* **340**, 610–614 (2013).
74. Wacker, D. *et al.* Structural features for functional selectivity at serotonin receptors. *Science.* **340**, 615–619 (2013).
75. Obosi, L. A., Hen, R., Beadle, D. J., Bermudez, I. & King, L. A. Mutational analysis of the mouse 5-HT7 receptor: Importance of the third intracellular loop for receptor-G-protein interaction. *FEBS Lett.* **412**, 321–324 (1997).
76. Doré, A. S. *et al.* Structure of the adenosine A2A receptor in complex with ZM241385 and the xanthines XAC and caffeine. *Structure* **19**, 1283–1293 (2011).
77. Olah, M. E. Identification of A2a adenosine receptor domains involved in selective coupling to Gs. *J. Biol. Chem.* **272**, 337–344 (1997).
78. Xu, F. *et al.* Structure of an agonist-bound human A(2A) adenosine receptor. *Science.* **332**, 318–322 (2011).
79. Jaakola, V.-P. *et al.* The 2.6 angstrom crystal structure of a human A(2A) adenosine receptor bound to an agonist. *Science.* **322**, 1211–1217 (2008).
80. Arden, J.R., Nagata, O., Shockley, M.S., Philip, M., Lameh, J., Sadee, W. Mutation Analysis of the third cytoplasmic loop domains in G-protein coupling of the HM1 Muscarinic Receptor. *Biochem. Biophys. Res. Commun.* **188**, 1111–1115 (1992).
81. Wong, S. K.-F., Parker, E. M. & Ross, E. M. Chimeric muscarinic cholinergic:β-adrenergic receptors that activate Gs in response to muscarinic agonists. *J. Biol. Chem.* **265**, 6219–6224 (1990).
82. Kubo, T. *et al.* Location of a region of the muscarinic acetylcholine receptor involved in selective effector coupling. *FEBS Lett.* **241**, 119–125 (1988).
83. Thal, D. M. *et al.* Crystal structures of the M1 and M4 muscarinic acetylcholine receptors. *Nature* **531**, 335–340 (2016).
84. Hogger, P., Shockley, M. S., Lameh, J. & Sadee, W. Activating and inactivating mutations in N- and C-terminal i3 loop junctions of muscarinic acetylcholine Hm1 receptors. *J. Biol. Chem.* **270**, 7405–7410 (1995).
85. Lameh, J. *et al.* Hm1 muscarinic cholinergic receptor internalization requires a domain in the third cytoplasmic loop. *J. Biol. Chem.* **267**, 13406–13412 (1992).
86. Moro, O., Shockley, M. S., Lameh, J. & Sadée, W. Overlapping multi-site domains of the muscarinic cholinergic Hm1 receptor involved in signal transduction and sequestration. *J. Biol. Chem.* **269**, 6651–6655 (1994).
87. Maeda, S. *et al.* Internalization of the Hm1 muscarinic cholinergic receptor involves the third cytoplasmic loop. *FEBS Lett.* **269**, 386–388 (1990).
88. Lee, N. H., Geoghagen, N. S. M., Cheng, E., Cline, R. T. & Fraser, C. M. Alanine scanning mutagenesis of conserved arginine/lysine- arginine/lysine-X-X-arginine/lysine G protein-activating motifs on m1 muscarinic acetylcholine receptors. *Mol. Pharmacol.* **50**, 140–148 (1996).
89. Shapiro, R. A. & Nathanson, N. M. Deletion analysis of the mouse m1 muscarinic acetylcholine receptor: effects on phosphoinositide metabolism and down-regulation. *Biochemistry* **28**, 8946–8950 (1989).
90. Wess, J., Bonner, T. I., Dorje, F. & Brann, M. R. Delineation of muscarinic receptor domains conferring selectivity of coupling to guanine nucleotide-binding proteins and second messengers. *Mol. Pharmacol.* **38**, 517–523 (1990).
91. Lechleiter, J. *et al.* Distinct sequence elements control the specificity of G protein

activation by muscarinic acetylcholine receptor subtypes. *EMBO J.* **9**, 4381–4390 (1990).
92. Haga, K. *et al.* Structure of the human M2 muscarinic acetylcholine receptor bound to an antagonist. *Nature* **482**, 547–551 (2012).
93. Blüml, K., Mutschler, E. & Wess, J. Functional role of a cytoplasmic aromatic amino acid in muscarinic receptor-mediated activation of phospholipase C. *J. Biol. Chem.* **269**, 11537–11541 (1994).
94. Wong, S. K. F. & Ross, E. M. Chimeric muscarinic cholinergic:β-adrenergic receptors that are functionally promiscuous among G proteins. *J. Biol. Chem.* **269**, 18968–18976 (1994).
95. Blin, N., Yun, J. & Wess, J. Mapping of single amino acid residues required for selective activation of G(q/11) by the m3 muscarinic acetylcholine receptor. *J. Biol. Chem.* **270**, 17741–17748 (1995).
96. Liu, J., Conklin, B. R., Blin, N., Yun, J. & Wess, J. Identification of a receptor/G-protein contact site critical for signaling specificity and G-prptein activation. *Proc. Natl. Acad. Sci. U. S. A.* **92**, 11642–11646 (1995).
97. Liu, J., Blin, N., Conklin, B. R. & Wess, J. Molecular mechanisms involved in muscarinic acetylcholine receptor-mediated G protein activation studied by insertion mutagenesis. *J. Biol. Chem.* **271**, 6172–6178 (1996).
98. Kruse, A. C. *et al.* Structure and dynamics of the M3 muscarinic acetylcholine receptor. *Nature* **482**, 552–556 (2012).
99. Blüml, K., Mutschler, E. & Wess, J. Insertion mutagenesis as a tool to predict the secondary structure of a muscarinic receptor domain determining specificity of G-protein coupling. *Proc. Natl. Acad. Sci. U. S. A.* **91**, 7980–7984 (1994).
100. Blüml, K., Mutschler, E. & Wess, J. Identification of an intracellular tyrosine residue critical for muscarinic receptor-mediated stimulation of phosphatidylinositol hydrolysis. *J. Biol. Chem.* **269**, 402–405 (1994).
101. Kunkel, M. T. & Peralta, E. G. Charged amino acids required for signal transduction by the m3 muscarinic acetylcholine receptor. *EMBO J.* **12**, 3809–3815 (1993).
102. Hill-Eubanks, D., Burstein, E. S., Spalding, T. A., Bräuner-Osborne, H. & Brann, M. R. Structure of a G-protein-coupling domain of a muscarinic receptor predicted by random saturation mutagenesis. *J. Biol. Chem.* **271**, 3058–3065 (1996).
103. Burstein, E. S., Spalding, T. A., Hill-Eubanks, D. & Brann, M. R. Structure-function of muscarinic receptor coupling to G proteins. *J. Biol. Chem.* **270**, 3141–3146 (1995).
104. Wu, D., Jiang, H. & Simon, M. I. Different α1-adrenergic receptor sequences required for activating different Gα subunits of Gq class of G proteins. *J. Biol. Chem.* **270**, 9828–9832 (1995).
105. Cotecchia, S., Exum, S., Caron, M. G. & Lefkowitz, R. J. Regions of the α1-adrenergic receptor involved in coupling to phosphatidylinositol hydrolysis and enhanced sensitivity of biological function. *Proc. Natl. Acad. Sci. U. S. A.* **87**, 2896–2900 (1990).
106. Kjelsberg, M. A., Cotecchia, S., Ostrowski, J., Caron, M. G. & Lefkowitz, R. J. Constitutive activation of the α(1B)-adrenergic receptor by all amino acid substitutions at a single site: Evidence for a region which constrains receptor activation. *J. Biol. Chem.* **267**, 1430–1433 (1992).
107. Eason, M. G. & Liggett, S. B. Chimeric mutagenesis of putative G-protein coupling domains of the α2A-adrenergic receptor. Localization of two redundant and fully competent G1 coupling domains. *J. Biol. Chem.* **271**, 12826–12832 (1996).

108. Wade, S. M. *et al.* Gi activator region of α2A-adrenergic receptors: distinct basic residues mediate Gi versus Gs activation. *Mol. Pharmacol.* **56**, 1005–1013 (1999).
109. Cotecchia, S., Ostrowski, J., Kjelsberg, M. A., Caron, M. G. & Lefkowitz, R. J. Discrete amino acid sequences of the α1-adrenergic receptor determine the selectivity of coupling to phosphatidylinositol hydrolysis. *J. Biol. Chem.* **267**, 1633–1639 (1992).
110. Cheung, A. H., Dixon, R. A. F., Hill, W. S., Sigal, I. S. & Strader, C. D. Separation of the structural requirements for agonist-promoted activation and sequestration of the β-adrenergic receptor. *Mol. Pharmacol.* **37**, 775–779 (1990).
111. Hausdorff, W. P., Hnatowich, M., O'Dowd, B. F., Caron, M. G. & Lefkowitz, R. J. A mutation of the β2-adrenergic receptor impairs agonist activation of adenylyl cyclase without affecting high affinity agonist binding. Distinct molecular determinants of the receptor are involved in physical coupling to and functional activation of G(s). *J. Biol. Chem.* **265**, 1388–1393 (1990).
112. Rosenbaum, D. M. *et al.* GPCR engineering yields high-resolution structural insights into β2-adrenergic receptor function. *Science.* **7**, 692–700 (2007).
113. Zou, Y., Weis, W. I. & Kobilka, B. K. N-terminal T4 lysozyme fusion facilitates crystallization of a G protein coupled receptor. *PLoS One* **7**, (2012).
114. Samama, P., Cotecchia, S., Costa, T. & Lefkowitz, R. J. A mutation-induced activated state of the β2-adrenergic receptor. Extending the ternary complex model. *J. Biol. Chem.* **268**, 4625–36 (1993).
115. Dixon, R. A. F. *et al.* Ligand binding to the β-adrenergic receptor involves its rhodopsin-like core. *Nature* **326**, 73–77 (1987).
116. Cheung, A. H., Huang, R.-R. C. & Strader, C. D. Involvement of specific hydrophobic, but not hydrophilic, amino acids in the third intracellular loop of the β-adrenergic receptor in the activation of Gs. *Mol. Pharmacol.* **41**, 1061–1065 (1992).
117. Cheung, A. H., Sigal, I. S., Dixon, R. A. & Strader, C. D. Agonist-promoted sequestration of the beta 2-adrenergic receptor requires regions involved in functional coupling with Gs. *Mol. Pharmacol.* **35**, 132–138 (1989).
118. Ohyama, K., Yamano, Y., Chaki, S., Kondo, T. & Inagami, T. Domains for G-protein coupling in angiotensin II receptor type I: studies by site-directed mutagenesis. *Biochem. Biophys. Res. Commun.* **189**, 677–683 (1992).
119. Wang, C., Jayadev, S. & Escobedo, J. A. Identification of a domain in the angiotensin II type 1 receptor determining Gq coupling by the use of receptor chimeras. *J. Biol. Chem.* **270**, 16677–16682 (1995).
120. Matsumoto, M. L., Narzinski, K., Kiser, P. D., Nikiforovich, G. V. & Baranski, T. J. A comprehensive structure-function map of the intracellular surface of the human C5a receptor. I. Identification of critical residues. *J. Biol. Chem.* **282**, 3105–3121 (2007).
121. Arai, H. & Charo, I. F. Differential regulation of G-protein-mediated signaling by chemokine receptors. *J. Biol. Chem.* **271**, 21814–21819 (1996).
122. Wu, B. *et al.* Structures of the CXCR4 chemokine GPCR with small-molecule and cyclic peptide antagonists. *Science.* **330**, 1066–1071 (2010).
123. Senogles, S. E., Heimert, T. L., Odife, E. R. & Quasney, M. W. A region of the third intracellular loop of the short form of the D2 dopamine receptor dictates Gi coupling specificity. *J. Biol. Chem.* **279**, 1601–1606 (2004).
124. Guiramand, J., Montmayeur, J. P., Ceraline, J., Bhatia, M. & Borrelli, E. Alternative splicing of the dopamine D2 receptor directs specificity of coupling to G-proteins. *J. Biol. Chem.* **270**, 7354–7358 (1995).

125. Montmayeur, J.-P., Guiramand, J. & Borrelli, E. Preferential coupling between dopamine D2 receptors and G-proteins. *Mol. Endocrinol.* **7**, 161–170 (1993).
126. Chien, E. Y. T. *et al.* Structure of the human dopamine D3 receptor in complex with a D2/D3 selective antagonist. *Science.* **330**, 1091–1095 (2010).
127. Srivastava, A. *et al.* High-resolution structure of the human GPR40 receptor bound to allosteric agonist TAK-875. *Nature* **513**, 124–127 (2014).
128. Wang, H. L. Basic amino acids at the C-terminus of the third intracellular loop are required for the activation of phospholipase C by cholecystokinin-B receptors. *J. Neurochem.* **68**, 1728–1735 (1997).
129. Mathi, S. K., Chan, Y., Li, X. & Wheeler, M. B. Scanning of the glucagon-like peptide-1 receptor localizes G protein- activating determinants primarily to the N terminus of the third intracellular loop. *Mol. Endocrinol.* **11**, 424–432 (1997).
130. Shimamura, T. *et al.* Structure of the human histamine H 1 receptor complex with doxepin. *Nature* **475**, 65–72 (2011).
131. Tseng, M. J., Coon, S., Stuenkel, E., Struk, V. & Logsdon, C. D. Influence of second and third cytoplasmic loops on binding, internalization, and coupling of chimeric bombesin/m3 muscarinic receptors. *J. Biol. Chem.* **270**, 17884–17891 (1995).
132. White, J. F. *et al.* Structure of the agonist-bound neurotensin receptor. *Nature* **490**, 508–513 (2012).
133. Egloff, P. *et al.* Structure of signaling-competent neurotensin receptor 1 obtained by directed evolution in Escherichia coli. *Proc. Natl. Acad. Sci. U. S. A.* **111**, (2014).
134. Krumm, B. E., White, J. F., Shah, P. & Grisshammer, R. Structural prerequisites for G-protein activation by the neurotensin receptor. *Nat. Commun.* **6**, (2015).
135. Kim, J. M. *et al.* Light-driven activation of β2-adrenergic receptor signaling by a chimeric rhodopsin containing the β2-adrenergic receptor cytoplasmic loops. *Biochemistry* **44**, 2284–2292 (2005).
136. Wu, H. *et al.* Structure of the human κ-opioid receptor in complex with JDTic. *Nature* **485**, 327–332 (2012).
137. Manglik, A. *et al.* Crystal structure of the μ-opioid receptor bound to a morphinan antagonist. *Nature* **485**, 321–326 (2012).
138. Franke, R. R., König, B., Sakmar, T. P., Khorana, H. G. & Hofmann, K. P. Rhodopsin mutants that bind but fail to activate transducin. **250**, 123–125 (1990).
139. Yamashita, T., Terakita, A. & Shichida, Y. Distinct roles of the second and third cytoplasmic loops of bovine rhodopsin in G protein activation. *J. Biol. Chem.* **275**, 34272–34279 (2000).
140. Yang, M. *et al.* Lysine 270 in the third intracellular domain of the oxytocin receptor is an important determinant for Gαq coupling specificity. *Mol. Endocrinol.* **16**, 814–823 (2002).
141. Zhang, K. *et al.* Structure of the human P2Y12 receptor in complex with an antithrombotic drug. *Nature* **508**, 115–118 (2014).
142. Pisegna, J. R. & Wank, S. A. Cloning and characterization of the signal transduction of four splice variants of the human pituitary adenylate cyclase activating polypeptide receptor. Evidence for dual coupling to adenylate cyclase and phospholipase C. *J. Biol. Chem.* **271**, 17267–17274 (1996).
143. Huang, Z. *et al.* The N-terminal region of the third intracellular loop of the parathyroid hormone (PTH)/PTH-related peptide receptor is critical for coupling to cAMP and inositol phosphate/Ca2+ signal transduction pathways. *J. Biol. Chem.* **271**, 33382–33389 (1996).

144. Boone, C., Davis, N. G. & Sprague, G. F. Mutations that alter the third cytoplasmic loop of the a-factor receptor lead to a constitutive and hypersensitive phenotype. *Proc. Natl. Acad. Sci. U. S. A.* **90**, 9921–9925 (1993).
145. Chazenbalk, G. D., Nagayama, Y., Russo, D., Wadsworth, H. L. & Rapoport, B. Functional analysis of the cytoplasmic domains of the human thyrotropin receptor by site-directed mutagenesis. *J. Biol. Chem.* **265**, 20970–20975 (1990).
146. Kosugi, S. *et al.* Mutation of alanine 623 in the third cytoplasmic loop of the rat thyrotropin (TSH) receptor results in a loss in the phosphoinositide but not cAMP signal induced by TSH and receptor autoantibodies. *J. Biol. Chem.* **267**, 24153–24156 (1992).
147. Kosugi, S. *et al.* Substitutions of different regions of the third cytoplasmic loop of the thyrotropin (TSH) receptor have selective effects on constitutive, TSH-, and TSH receptor autoantibody-stimulated phosphoinositide and 3',5'-cyclic adenosine monophosphate signal gene. *Mol. Endocrinol.* **7**, 1009–1020 (1993).
148. Erlenbach, I. & Wess, J. Molecular basis of V2 vasopressin receptor/G(s) coupling selectivity. *J. Biol. Chem.* **273**, 26549–26558 (1998).
149. Cotecchia, S., Ostrowski, J., Kjelsberg, M. A., Caron, M. G. & Lefkowitz, R. J. Discrete amino acid sequences of the α1-adrenergic receptor determine the selectivity of coupling to phosphatidylinositol hydrolysis. *J. Biol. Chem.* **267**, 1633–1639 (1992).
150. Carlson, S. A., Chatterjee, T. K. & Fisher, R. A. The third intracellular domain of the platelet-activating factor receptor is a critical determinant in receptor coupling to phosphoinositide phospholipase C-activating G proteins. Studies using intracellular domain minigenes and receptor chimeras. *J. Biol. Chem.* **271**, 23146–23153 (1996).
