# Supplementary material for: Autoregulation of GPCR signalling through the third intracellular loop
Source: Nature. 2023 Mar 8;615(7953):734–41. doi: 10.1038/s41586-023-05789-z (PMC10033409; doi:10.1038/s41586-023-05789-z)
Supplement: Supplementary file 2 — Reporting Summary [file 41586_2023_5789_MOESM2_ESM.pdf]

Corresponding author(s): Sivaraj Sivaramakrishnan
Last updated by author(s): Jan 25, 2023

Reporting Summary

Nature Portfolio wishes to improve the reproducibility of the work that we publish. This form provides structure for consistency and transparency in reporting. For further information on Nature Portfolio policies, see our Editorial Policies and the Editorial Policy Checklist.

Statistics

For all statistical analyses, confirm that the following items are present in the figure legend, table legend, main text, or Methods section.

n/a	Confirmed	
☐	☒	The exact sample size (*n*) for each experimental group/condition, given as a discrete number and unit of measurement
☐	☒	A statement on whether measurements were taken from distinct samples or whether the same sample was measured repeatedly
☐	☒	The statistical test(s) used AND whether they are one- or two-sided<br>*Only common tests should be described solely by name; describe more complex techniques in the Methods section.*
☒	☐	A description of all covariates tested
☐	☒	A description of any assumptions or corrections, such as tests of normality and adjustment for multiple comparisons
☐	☒	A full description of the statistical parameters including central tendency (e.g. means) or other basic estimates (e.g. regression coefficient) AND variation (e.g. standard deviation) or associated estimates of uncertainty (e.g. confidence intervals)
☐	☒	For null hypothesis testing, the test statistic (e.g. *F*, *t*, *r*) with confidence intervals, effect sizes, degrees of freedom and *P* value noted<br>*Give P values as exact values whenever suitable.*
☒	☐	For Bayesian analysis, information on the choice of priors and Markov chain Monte Carlo settings
☒	☐	For hierarchical and complex designs, identification of the appropriate level for tests and full reporting of outcomes
☒	☐	Estimates of effect sizes (e.g. Cohen's *d*, Pearson's *r*), indicating how they were calculated

Our web collection on statistics for biologists contains articles on many of the points above.

Software and code

Policy information about availability of computer code

Data collection	We used GROMACS-2016.3 for molecular dynamics simulations.
Data analysis	Free energy landscape in Figure 2B was created using MSMBuilder2 (Version 3.8.0). Fluorescence lifetime data were fit in DAS6 (Horiba). Statistical analyses were performed in RStudio (version 2022.12.0). Curve fits were performed in Excel using the Solver add-in. Figures were generated in RStudio using the ggplot2 package. Image processing was performed in Fiji. Molecular structure representations were created using VMD (version 1.9.3) and Pymol (version 2.0.6).

For manuscripts utilizing custom algorithms or software that are central to the research but not yet described in published literature, software must be made available to editors and reviewers. We strongly encourage code deposition in a community repository (e.g. GitHub). See the Nature Portfolio guidelines for submitting code & software for further information.

Data

Policy information about availability of data

All manuscripts must include a data availability statement. This statement should provide the following information, where applicable:

- Accession codes, unique identifiers, or web links for publicly available datasets
- A description of any restrictions on data availability
- For clinical datasets or third party data, please ensure that the statement adheres to our policy

All source data are included in the manuscript, including a "Source Data" spreadsheet. Simulation data is stored on the MD database for GPCRs (GPCRmd.org) under

dynamics ID 1247. Receptor structure files 3sn6, 2ycx, 6e67, 5jqh, and 4ldl were obtained from the Protein Data Bank (rcsb.org). G protein coupling data was obtained from the G protein database (https://gproteindb.org/signprot/couplings).

Human research participants

Policy information about studies involving human research participants and Sex and Gender in Research.

Reporting on sex and gender	This study did not involve human research participants.
Population characteristics	N/A
Recruitment	N/A
Ethics oversight	N/A

Note that full information on the approval of the study protocol must also be provided in the manuscript.

Field-specific reporting

Please select the one below that is the best fit for your research. If you are not sure, read the appropriate sections before making your selection.

☒ Life sciences ☐ Behavioural & social sciences ☐ Ecological, evolutionary & environmental sciences

For a reference copy of the document with all sections, see nature.com/documents/nr-reporting-summary-flat.pdf

Life sciences study design

All studies must disclose on these points even when the disclosure is negative.

Sample size	Sample sizes for individual experiments were maximized based on physical constraints (amount of sample or timing). Number of individual experiments performed was based on the reproducibility of each result (i.e. smaller, variable effects were attempted 3+ times to ensure reproducibility, while larger effects with less variability were performed 3-4 times).
Data exclusions	When controllable parameters (e.g. receptor expression and cell count) failed to match desired conditions in second messenger assays, as indicated in the Methods, data were excluded from analysis. Excluded data are highlighted in the source data file.
Replication	Experiments were replicated independently at least three times to ensure reproducibility. With the exception of the data exclusions indicated above, all experiments were reproduced successfully.
Randomization	Conditions for biological samples [membranes, cells, vesicles] were plated and/or assayed in random order between experimental replicates for all datasets.
Blinding	Investigators were not blinded to group allocation during data collection or analysis, as all data presented are quantitative and no subjective metrics were assessed.

Reporting for specific materials, systems and methods

We require information from authors about some types of materials, experimental systems and methods used in many studies. Here, indicate whether each material, system or method listed is relevant to your study. If you are not sure if a list item applies to your research, read the appropriate section before selecting a response.

Materials & experimental systems

n/a	Involved in the study	
☐	☒	Antibodies
☐	☒	Eukaryotic cell lines
☒	☐	Palaeontology and archaeology
☒	☐	Animals and other organisms
☒	☐	Clinical data
☒	☐	Dual use research of concern

Methods

n/a	Involved in the study	
☒	☐	ChIP-seq
☒	☐	Flow cytometry
☒	☐	MRI-based neuroimaging

Antibodies

Antibodies used	Nanobody 6B9 (lama glama)- expressed in Escherichia coli and purified using affinity and size exclusion chromatography in house.<br>Terbium cryptate labeled anti-IP1 antibody - reagent in IP1 assay kit (CisBio catalog #62IPAPEB).
Validation	Nanobody 6B9 binding to the active state beta 2 adrenergic receptor was validated through a competition binding experiment (Supplementary Figure 20A).<br>Terbium cryptate labeled anti-IP1 antibody was validated according to the manufacturer's protocol.

Eukaryotic cell lines

Policy information about cell lines and Sex and Gender in Research

Cell line source(s)	HEK 293T Flp-In T-Rex- Thermo Fisher cat#R78007
Authentication	Cell line was not authenticated.
Mycoplasma contamination	Cell line was not tested for Mycoplasma contamination.
Commonly misidentified lines<br>(See ICLAC register)	No commonly misidentified cell lines were used in this study.
